# Supplementary material for: The health effects associated with physical, sexual and psychological gender-based violence against men and women: a Burden of Proof study
Source: Nat Hum Behav. 2025 Apr 10;9(6):1201–16. doi: 10.1038/s41562-025-02144-2 (PMC12185316; doi:10.1038/s41562-025-02144-2)
Supplement: Supplementary file 1 — Supplementary Methods and Results. [file 41562_2025_2144_MOESM1_ESM.pdf]

nature human behaviour

Supplementary information https://doi.org/10.1038/s41562-025-02144-2

The health effects associated with physical, sexual and psychological gender-based violence against men and women: a Burden of Proof study

In the format provided by the authors and unedited

Supplementary Information: Data Sources and Supplementary Results for “The health effects associated with physical, sexual and psychological gender-based violence against men and women: a Burden of Proof study "

This appendix provides detailed information on input data sources and supplementary results for the publication titled “The health effects associated with physical, sexual and psychological gender-based violence against men and women: a Burden of Proof study.”

Table of Contents

Section 1: Summary characteristics of included studies ... 6

Table S1. Effect size data details for physical, psychological, and sexual GBV ... 6

Section 2: Sensitivity Analyses ... 15

Section 2.1: Sensitivity analyses results for GBV exposures and outcomes ... 15

Table S2. Sensitivity analysis results for GBV exposure and alcohol use disorder ... 15

Table S3. Sensitivity analysis results for GBV exposure and drug use disorders ... 15

Table S4. Sensitivity analysis results for GBV exposure and HIV/AIDS ... 16

Table S5. Sensitivity analysis results for GBV exposure and major depressive disorder ... 17

Table S6. Sensitivity analysis results for GBV exposure and anxiety disorders ... 18

Table S7. Sensitivity analysis results for GBV exposure and maternal abortion and miscarriage ... 18

Table S8. Sensitivity analysis results for GBV exposure and self-harm ... 20

Section 2.2: Sensitivity analyses forest plots for physical GBV and the corresponding outcomes ... 21

Figure S1. Sensitivity analysis results for physical GBV exposure (males only) and outcomes ... 21

Figure S2. Sensitivity analysis results for physical GBV exposure (females only) and outcomes ... 21

Figure S3. Sensitivity analysis results for physical GBV exposure (non-pregnancy recall) and outcomes ... 22

Figure S4. Sensitivity analysis results for physical GBV exposure (pregnancy-specific recall) and outcomes ... 22

Figure S5. Sensitivity analysis results for perpetrator-specific physical GBV exposure and outcomes ... 23

Figure S6. Sensitivity analysis results for physical GBV exposure and alternative outcome definitions ... 23

Figure S7. Sensitivity analysis results for physical GBV exposure (no adjustment) and outcomes ... 24

Figure S8. Sensitivity analysis results for physical GBV exposure (no trimming) and outcomes ... 25

Section 2.3: Sensitivity analyses forest plots for sexual violence exposures and the corresponding outcomes ... 26

Figure S9. Sensitivity analysis results for perpetrator-specific sexual violence exposure and outcomes ... 26

Figure S10. Sensitivity analysis results for sexual violence exposure and alternative outcome definitions ... 26
Figure S11. Sensitivity analysis results for sexual violence exposure (females only) and outcomes ... 27
Figure S12. Sensitivity analysis results for sexual violence exposure (non-pregnancy recall) and outcomes ... 27
Figure S13. Sensitivity analysis results for sexual violence exposure by any perpetrator and outcomes ... 28
Figure S14. Sensitivity analysis results for sexual violence exposure (no adjustment) and outcomes ... 28
Figure S15. Sensitivity analysis results for sexual violence exposure (no trimming) and outcomes ... 29
Section 2.4: Sensitivity analyses forest plots for psychological GBV and the corresponding outcomes ... 30
Figure S16. Sensitivity analysis results for psychological GBV exposure and alternative outcome definitions ... 30
Figure S17. Sensitivity analysis results for psychological GBV exposure (females only) and outcomes ... 30
Figure S18. Sensitivity analysis results for psychological GBV exposure (non-pregnancy recall) and outcomes ... 31
Figure S19. Sensitivity analysis results for perpetrator-specific psychological GBV exposure and outcomes ... 31
Figure S20. Sensitivity analysis results for psychological GBV exposure (no adjustments) and definitions ... 32
Figure S21. Sensitivity analysis results for psychological GBV exposure (no trimming) and definitions ... 32
Section 3: PRISMA and GATHER Checklists ... 33
Section 3.1: GATHER ... 33
Table S9. GATHER checklist ... 33
Section 3.2: PRISMA ... 33
Table S10. PRISMA 2020 abstract checklist ... 33
Table S11. PRISMA 2020 checklist ... 35
Section 4: Data source identification and assessment ... 39
Section 4.1: Literature searches ... 39
PubMed Search String ... 39
Embase Search String ... 40

Cumulative Index to Nursing and Allied Health Literature (CINAHL) Search String............. 41
PsycINFO Search String.. 42
Global Index Medicus Search String.. 44
Cochrane Search String... 45
Web of Science Core Collection Search String 46
Section 4.2: Inclusion and exclusion criteria 47
Table S12. Inclusion and exclusion criteria applied during screening.............................. 47
Table S13. Inclusion and exclusion criteria applied during data cleaning......................... 48
Section 4.3: Systematic review and meta-analysis citation searching 48
Section 4.4: Data Extraction.. 49
Table S14. Data extraction template.. 49
Section 5: Differences between Spencer et al. (2023) and current study.............................. 52
Table S15. Differences between Spencer et al. (2023) and current study.......................... 53
Figure S22. Comparison between mean relative risk and strength of the evidence for the association of multiple forms of GBV and IPV and 8 health outcomes 55
Section 6: Included Exposure and Outcome Definitions 55
Section 6.1: Exposure and outcome definitions 55
Table S16. Definitions of included risk factors 55
Table S17. Summarized exposure definitions for GBV used in included studies................. 56
Table S18. Definitions of included outcomes... 57
Section 6.1.1: Additional description of accepted definitions and measurement tools for depressive and anxiety disorders 58
Table S19. Depressive and anxiety disorder accepted diagnostic interview and symptom scale measurement tools 58
Section 6.1.2: Additional description of accepted definitions for substance use disorders. 59
Section 7: Study Quality and Bias Assessment 60
Section 7.1: Definition of bias covariates.. 60
Table S20. Standard bias covariates created across all input datasets.............................. 61
Table S21. Adjustment bias covariates created across all input datasets 61
Table S22. Exposure definition bias covariates 61
Table S23. Outcome definition bias covariates.. 62
Section 7.2: Bias covariates for each risk-outcome pair 63
Table S24. Bias covariates for physical GBV 63

Table S25. Bias covariates for sexual GBV 64
Table S26. Bias covariates for psychological GBV 66
Section 8: Primary analysis funnel plots for GBV exposures and outcomes 67
Section 8.1: Primary analysis funnel plots for sexual violence and outcomes 67
Figure S23. Primary analysis funnel plot for sexual violence and maternal abortion and miscarriage 67
Figure S24. Primary analysis funnel plot for sexual violence and anxiety disorders 67
Figure S25. Primary analysis funnel plot for sexual violence and major depressive disorder 68
Figure S26. Primary analysis funnel plot for sexual violence and drug use disorders 68
Figure S27. Primary analysis funnel plot for sexual violence and HIV/AIDS 68
Figure S28. Primary analysis funnel plot for sexual violence and sexually transmitted infections (excluding HIV) 69
Section 8.2: Primary analysis funnel plots for physical GBV and outcomes 70
Figure S29. Primary analysis funnel plot for physical GBV and maternal abortion and miscarriage 70
Figure S30. Primary analysis funnel plot for physical GBV and alcohol use disorders 70
Figure S31. Primary analysis funnel plot for physical GBV and anxiety disorders 71
Figure S32. Primary analysis funnel plot for physical GBV and major depressive disorders 71
Figure S33. Primary analysis funnel plot for physical GBV and drug use disorders 72
Figure S34. Primary analysis funnel plot for physical GBV and HIV/AIDS 72
Figure S35. Primary analysis funnel plot for physical GBV and self-harm 73
Figure S36. Primary analysis funnel plot for physical GBV and sexually transmitted infections (excluding HIV) 73
Section 8.3: Primary analysis funnel plots for psychological GBV and outcomes 74
Figure S37. Primary analysis funnel plot for psychological GBV and maternal abortion and miscarriage 74
Figure S38. Primary analysis funnel plot for psychological GBV and major depressive disorder 74
Figure S39. Primary analysis funnel plot for psychological GBV and drug use disorders 75
Figure S40. Primary analysis funnel plot for psychological GBV and self-harm 75
Section 9: Model Characteristics 75
Table S27. Model characteristics of GBV and corresponding health outcomes 76
Section 10: Data for the Primary Analyses 76
Table S28. Data inputs included in the primary analyses 76

Section 1: Summary characteristics of included studies

Table S1. Effect size data details for physical, psychological, and sexual GBV

Author	Year	Location	Study Design	Type of Violence	Exposure Assessment Method	Exposure Recall	Type of Perpetrator	Follow-up Years	Outcome	Type of Outcome	Outcome Assessment Method	Age Summary	Gender	Sample Size	Number of Cases (Exposed Group)	Number of Cases (Unexposed Group)
Leung	2002	Hong Kong Special Administrative Region Of China	Case-Control	Physical	Self-Report or Self-Administered Survey	Past Year	Partner; Former Partner		Termination Of Pregnancy	Mortality	administrative medical records or disease registries	27 (8)	Women	501	15	
Ibrahim	2015	Egypt	Prospective Cohort	Physical	Self-Report or Self-Administered Survey; Clinical Examination	During Pregnancy	Partner; Former Partner		Complete Abortion	Incidence	administrative medical records or disease registries	18–43	Women	1857	25	12
Hailu	2023	Tigray	Case-Control	Physical	Structured Interviewer-Administered Questionnaire	During Pregnancy	Partner		Spontaneous Abortion	Mortality	administrative medical records or disease registries	26	Women	371	48	76
Abdollahi	2015	Mazandaran	Prospective Cohort	Physical	Self-Report or Self-Administered Survey	During Pregnancy	Partner	0.5	Abortion	Incidence	administrative medical records or disease registries	18–45	Women	1461	42	9
Catak	2016	Turkey	Case-Control	Physical	Self-Report or Self-Administered Survey	Undefined	Partner		Spontaneous Abortion	Mortality	physician diagnosis	15–49	Women	752	38	
Johri	2011	Guatemala	Case-Control	Physical	Self-Report or Self-Administered Survey	Past Year	Partner		Miscarriage	Mortality	physician diagnosis	15–49	Women	1897		
Bourassa	2007	Canada	Case-Control	Physical	Self-Report or Self-Administered Survey	Past Year	Anyone or Not-Specified		Elective Abortion	Mortality	administrative medical records or disease registries	25.3	Women	1003	27	
Nelson	2003	Pennsylvania	Case-Control	Physical	Self-Report or Self-Administered Survey	During Pregnancy	Family Member or Caregiver; Stranger; Acquaintance		Spontaneous Abortion	Mortality	self-report; administrative medical records or disease registries	14–40	Women	1199	20	372

Author	Year	Location	Study Design	Type of Violence	Exposure Assessment Method	Exposure Recall	Type of Perpetrator	Follow-up Years	Outcome	Type of Outcome	Outcome Assessment Method	Age Summary	Gender	Sample Size	Number of Cases (Exposed Group)	Number of Cases (Unexposed Group)
Ehrensaft	2006	New Zealand	Prospective Cohort	Physical	Self-Report or Self-Administered Survey	Last 3 Years	Partner; Former Partner	8	Alcohol Dependence	Incidence	self-report	26	Women	449	6	41
Ehrensaft	2006	New Zealand	Prospective Cohort	Physical	Self-Report or Self-Administered Survey	Last 3 Years	Partner; Former Partner	8	Alcohol Dependence	Incidence	self-report	26	Men	456	15	88
Danielson	2009	USA	Prospective Cohort	Physical	Self-Report or Self-Administered Survey	Lifetime	Anyone or Not-Specified	8	Alcohol Abuse	Incidence	self-report	20–26	Women	872		
Ahmadabadi	2019	Australia	Prospective Cohort	Physical	Self-Report or Self-Administered Survey	Lifetime	Partner; Former Partner	30	Alcohol Disorder	Incidence	self-report	30–30	Men	531		
Ahmadabadi	2019	Australia	Prospective Cohort	Physical	Self-Report or Self-Administered Survey	Lifetime	Partner; Former Partner	30	Alcohol Disorder	Incidence	self-report	30–30	Women	822		
Hedtke	2008	USA	Prospective Cohort	Physical	Self-Report or Self-Administered Survey	Lifetime	Anyone or Not-Specified	2	Past-Year Posttraumatic Stress Disorder (Ptsd) Wave 3	Incidence	self-report	18–91	Women	1302		
Ehrensaft	2006	New Zealand	Prospective Cohort	Physical	Self-Report or Self-Administered Survey	Last 3 Years	Partner; Former Partner	8	Generalized Anxiety Disorder	Incidence	self-report	26	Women	449	7	16
Ehrensaft	2006	New Zealand	Prospective Cohort	Physical	Self-Report or Self-Administered Survey	Last 3 Years	Partner; Former Partner	8	Generalized Anxiety Disorder	Incidence	self-report	26	Men	456	5	12
Ehrensaft	2006	New Zealand	Prospective Cohort	Physical	Self-Report or Self-Administered Survey	Past 3 Years	Partner	8	Posttraumatic Stress Disorder	Incidence	self-report	26	Men	456		
Ehrensaft	2006	New Zealand	Prospective Cohort	Physical	Self-Report or Self-Administered Survey	Past 3 Years	Partner	8	Posttraumatic Stress Disorder	Incidence	self-report	26	Women	449		
Ahmadabadi	2020	Australia	Prospective Cohort	Physical	Self-Report or Self-Administered Survey	Lifetime	Partner; Former Partner	30	Anxiety Disorders	Incidence	self-report	30–30	Men	638		
Ahmadabadi	2020	Australia	Prospective Cohort	Physical	Self-Report or Self-Administered Survey	Lifetime	Partner; Former Partner	30	Anxiety Disorders	Incidence	self-report	30–30	Women	891		
Hedtke	2008	USA	Prospective Cohort	Physical	Self-Report or Self-Administered Survey	Lifetime	Anyone or Not Specified	2	Past-Year Major Depressive Episode (Mde) Wave 3	Incidence	self-report	18–91	Women	1302		
Ehrensaft	2006	New Zealand	Prospective Cohort	Physical	Self-Report or Self-Administered Survey	Last 3 Years	Partner; Former Partner	8	Depression	Incidence	self-report	26	Women	449	15	78

Author	Year	Location	Study Design	Type of Violence	Exposure Assessment Method	Exposure Recall	Type of Perpetrator	Follow-up Years	Outcome	Type of Outcome	Outcome Assessment Method	Age Summary	Gender	Sample Size	Number of Cases (Exposed Group)	Number of Cases (Unexposed Group)
Ehrensaft	2006	New Zealand	Prospective Cohort	Physical	Self-Report or Self-Administered Survey	Last 3 Years	Partner; Former Partner	8	Depression	Incidence	self-report	26	Men	456	4	46
Han	2019	Republic Of Korea	Prospective Cohort	Physical	Self-Report or Self-Administered Survey	Past Year	Partner	1	Mild, Moderate, or Severe, Depression	Incidence	self-report	19–99	Women	3052	37	517
Han	2019	Republic Of Korea	Prospective Cohort	Physical	Self-Report or Self-Administered Survey	Past Year	Partner	1	Mild, Moderate, or Severe, Depression	Incidence	self-report	19–99	Men	3053	4	409
Ahmadabadi	2020	Australia	Prospective Cohort	Physical	Self-Report or Self-Administered Survey	Lifetime	Partner; Former Partner	30	Major Depression Disorder	Incidence	self-report	30–30	Women	891		
Ahmadabadi	2020	Australia	Prospective Cohort	Physical	Self-Report or Self-Administered Survey	Lifetime	Partner; Former Partner	30	Major Depression Disorder	Incidence	self-report	30–30	Men	638		
Martino	2005	Multiple	Prospective Cohort	Physical	Self-Report or Self-Administered Survey	Past 3 Years At Wave 8	Partner; Former Partner	6	Past Year Use Of Other Illicit Substances	Incidence	self-report	29–29	Women	509		
Gilbert	2012	New York	Prospective Cohort	Physical	Self-Report or Self-Administered Survey	Past 6 Months At Wave 1	Partner; Former Partner	1	Use Of Hard Drugs (Crack/Cocaine And/Or Heroin)	Incidence	self-report	32.8 (10.08)	Women	241		
Nowotny	2013	USA	Prospective Cohort	Physical	Self-Report or Self-Administered Survey	Reported At T1 Wave Iii (2001 - 2002)	Partner	7	Drug Use	Incidence	self-report	29	Women	2959		
Ehrensaft	2006	New Zealand	Prospective Cohort	Physical	Self-Report or Self-Administered Survey	Last 3 Years	Partner; Former Partner	8	Marijuana Dependence	Incidence	self-report	26	Women	449	11	8
Ehrensaft	2006	New Zealand	Prospective Cohort	Physical	Self-Report or Self-Administered Survey	Last 3 Years	Partner; Former Partner	8	Marijuana Dependence	Incidence	self-report	26	Men	456	8	46
Danielson	2009	USA	Prospective Cohort	Physical	Self-Report or Self-Administered Survey	Lifetime	Anyone or Not-Specified	8	Drug Abuse	Incidence	self-report	20–26	Women	872		
Ahmadabadi	2019	Australia	Prospective Cohort	Physical	Self-Report or Self-Administered Survey	Lifetime	Partner; Former Partner	30	Drug Disorder	Incidence	self-report	30–30	Men	531		
Ahmadabadi	2019	Australia	Prospective Cohort	Physical	Self-Report or Self-Administered Survey	Lifetime	Partner; Former Partner	30	Drug Disorder	Incidence	self-report	30–30	Women	822		
Fonck	2005	Nairobi	Other (Cross-Sectional)	Physical	Self-Report or Self-Administered Survey	Lifetime	Partner; Former Partner		HIV Positive Status	Incidence	biomarker	27	Women	520	39	

Author	Year	Location	Study Design	Type of Violence	Exposure Assessment Method	Exposure Recall	Type of Perpetrator	Follow-up Years	Outcome	Type of Outcome	Outcome Assessment Method	Age Summary	Gender	Sample Size	Number of Cases (Exposed Group)	Number of Cases (Unexposed Group)
Deyessa	2018	Addis Ababa	Case-Control	Physical	Self-Report or Self-Administered Survey	Lifetime	Partner; Former Partner		HIV	Mortality	biomarker	15–49	Women	510	46	
Maman	2002	United Republic Of Tanzania	Prospective Cohort	Physical	Self-Report or Self-Administered Survey	Lifetime	Partner	0.25	HIV Positive	Incidence	biomarker	32	Women	245	38	
Kouyoumdjian	2013	Uganda	Prospective Cohort	Physical	Self-Report or Self-Administered Survey	Lifetime	Partner; Former Partner	5.5	HIV/Aids	Incidence	biomarker	15–49	Women		194	152
Kaslow	2000	USA	Case-Control	Physical	Self-Report or Self-Administered Survey	Lifetime	Partner		Suicide Attempt	Mortality	presenting to a hospital	30.8 (8.96)	Women	285	41	
Bella	2012	Argentina	Case-Control	Physical	Self-Report or Self-Administered Survey	Lifetime	Anyone or Not-Specified		Suicide Attempt	Mortality	administrative medical records or disease registries	13	Combined Men/Women	66	9	
Chowdhary	2008	Goa	Prospective Cohort	Physical	Self-Report or Self-Administered Survey	Lifetime	Partner	1	Attempted Suicide	Incidence	self-report; biomarker	18–50	Women	1537		
Allsworth	2009	Rhode Island	Prospective Cohort	Physical	Self-Report or Self-Administered Survey	Past Year	Anyone or Not-Specified	1.6	Incident STI	Incidence	administrative medical records or disease registries; physician diagnosis; biomarker	13–35	Women		19	56
Weiss	2008	Goa, Rural	Prospective Cohort	Physical	Self-Report or Self-Administered Survey	Lifetime	Partner	1	Sexually Transmitted Infection (STI)	Incidence	physician diagnosis; biomarker	18–45	Women	1552	9	
Chowdhary	2008	Goa	Prospective Cohort	Physical	Self-Report or Self-Administered Survey	Lifetime	Partner	1	Sexually Transmitted Infection (STI)	Incidence	self-report; biomarker	18–50	Women	1498		
Leung	2002	Hong Kong Special Administrative Region Of China	Case-Control	Psychological	Self-Report or Self-Administered Survey	Lifetime	Partner		Termination Of Pregnancy	Mortality	administrative medical records or disease registries	27 (8)	Women	501	18	
Hailu	2023	Tigray	Case-Control	Psychological	Structured Interviewer-Administered Questionnaire	During Pregnancy	Partner		Spontaneous Abortion	Mortality	administrative medical records or disease registries	26	Women	371	54	70

Author	Year	Location	Study Design	Type of Violence	Exposure Assessment Method	Exposure Recall	Type of Perpetrator	Follow-up Years	Outcome	Type of Outcome	Outcome Assessment Method	Age Summary	Gender	Sample Size	Number of Cases (Exposed Group)	Number of Cases (Unexposed Group)
Johri	2011	Guatemala	Case-Control	Psychological	Self-Report or Self-Administered Survey	Past Year	Partner		Miscarriage	Mortality	physician diagnosis	15–49	Women	1897		
Bourassa	2007	Canada	Case-Control	Psychological	Self-Report or Self-Administered Survey	Lifetime	Anyone or Not-Specified		Elective Abortion	Mortality	administrative medical records or disease registries	25.3	Women	1003	42	
Nelson	2003	Pennsylvania	Case-Control	Psychological	Self-Report or Self-Administered Survey	Lifetime	Partner; Former Partner		Spontaneous Abortion	Mortality	self-report; administrative medical records or disease registries	14–40	Women	1199		
Romito	2009	Friuli-Venezia Giulia	Case-Control	Psychological	Self-Report or Self-Administered Survey	Past Year	Partner		Elective Abortion (Ea)	Mortality	administrative medical records or disease registries	13–29	Women	301	16	154
Romito	2009	Friuli-Venezia Giulia	Case-Control	Psychological	Self-Report or Self-Administered Survey	Past Year	Partner		Elective Abortion (Ea)	Mortality	administrative medical records or disease registries	30–99	Women	582	12	229
Fitzpatrick	2023	Australia	Prospective Cohort	Psychological	Self-Report or Self-Administered Survey	First And/Or Fourth Year Postpartum	Partner; Former Partner	10	Major Depression	Incidence	self-report	28–60	Women	1143		
Fitzpatrick	2023	Australia	Prospective Cohort	Psychological	Self-Report or Self-Administered Survey	Past Year	Partner; Former Partner	10	Major Depression	Incidence	self-report	28–60	Women	1143		
Han	2019	Republic Of Korea	Prospective Cohort	Psychological	Self-Report or Self-Administered Survey	Past Year	Partner	1	Mild, Moderate, or Severe, Depression	Incidence	self-report	19–99	Women	2429	40	376
Han	2019	Republic Of Korea	Prospective Cohort	Psychological	Self-Report or Self-Administered Survey	Past Year	Partner	1	Mild, Moderate, or Severe, Depression	Incidence	self-report	19–99	Men	2488	15	312
Ahmadabadi	2020	Australia	Prospective Cohort	Psychological	Self-Report or Self-Administered Survey	Lifetime	Partner; Former Partner	30	Major Depression Disorder	Incidence	self-report	30–30	Women	891		
Gilbert	2012	New York	Prospective Cohort	Psychological	Self-Report or Self-Administered Survey	Past 6 Months At Wave 1	Anyone or Not-Specified	1	Use Of Hard Drugs (Crack/Cocaine And/Or Heroin)	Incidence	self-report	32.8 (10.08)	Women	241		

Author	Year	Location	Study Design	Type of Violence	Exposure Assessment Method	Exposure Recall	Type of Perpetrator	Follow-up Years	Outcome	Type of Outcome	Outcome Assessment Method	Age Summary	Gender	Sample Size	Number of Cases (Exposed Group)	Number of Cases (Unexposed Group)
Exner-Cortens	2013	USA	Retrospective Cohort	Psychological	Self-Report or Self-Administered Survey	Past Year	Partner	5	Drug Use	Incidence	self-report	16 (0.1)	Women			
Exner-Cortens	2013	USA	Retrospective Cohort	Psychological	Self-Report or Self-Administered Survey	Past Year	Partner	5	Drug Use	Incidence	self-report	16 (0.1)	Men			
Ahmadabadi	2019	Australia	Prospective Cohort	Psychological	Self-Report or Self-Administered Survey	Lifetime	Partner; Former Partner	30	Drug Disorder	Incidence	self-report	30–30	Women	822		
Ahmadabadi	2019	Australia	Prospective Cohort	Psychological	Self-Report or Self-Administered Survey	Lifetime	Partner; Former Partner	30	Drug Disorder	Incidence	self-report	30–30	Men	531		
Kaslow	2000	USA	Case-Control	Psychological	Self-Report or Self-Administered Survey	Lifetime	Partner		Suicide Attempt	Mortality	presenting to a hospital	30.8 (8.96)	Women	285		
Chowdhary	2008	Goa	Prospective Cohort	Psychological	Self-Report or Self-Administered Survey	Lifetime	Partner	1	Attempted Suicide	Incidence	self-report; biomarker	18–50	Women	1537		
Exner-Cortens	2013	USA	Retrospective Cohort	Psychological	Self-Report or Self-Administered Survey	Past Year	Partner	5	Suicide Attempt	Incidence	self-report	16 (0.1)	Men			
Exner-Cortens	2013	USA	Retrospective Cohort	Psychological	Self-Report or Self-Administered Survey	Past Year	Partner	5	Suicide Attempt	Incidence	self-report	16 (0.1)	Women			
Leung	2002	Hong Kong Special Administrative Region Of China	Case-Control	Sexual	Self-Report or Self-Administered Survey	Past Year	Partner; Former Partner		Termination Of Pregnancy	Mortality	administrative medical records or disease registries	27 (8)	Women	501	24	
Hailu	2023	Tigray	Case-Control	Sexual	Structured Interviewer-Administered Questionnaire	During Pregnancy	Partner		Spontaneous Abortion	Mortality	administrative medical records or disease registries	26	Women	371	21	103
Johri	2011	Guatemala	Case-Control	Sexual	Self-Report or Self-Administered Survey	Past Year	Partner		Miscarriage	Mortality	physician diagnosis	15–49	Women	1897		
Bourassa	2007	Canada	Case-Control	Sexual	Self-Report or Self-Administered Survey	Past Year	Anyone or Not Specified		Elective Abortion	Mortality	administrative medical records or disease registries	25.3	Women	1003	15	
Larsen	2016	Denmark	Prospective Cohort	Sexual	Routinely Collected/Administrative Data ; Attending Center For Victims Of Sexual Assault	Lifetime	Anyone or Not-Specified	9.7	Induced Abortion At Any Stage Of Pregnancy	Incidence	administrative medical records or disease registries	12–93	Women	12505	493	730
Larsen	2016	Denmark	Prospective Cohort	Sexual	Routinely Collected/Administrative Data ; Attending Center	Lifetime	Anyone or Not-Specified	9.7	Spontaneous Abortion	Incidence	administrative medical records or	12–93	Women	12505	82	207

Author	Year	Location	Study Design	Type of Violence	Exposure Assessment Method	Exposure Recall	Type of Perpetrator	Follow-up Years	Outcome	Type of Outcome	Outcome Assessment Method	Age Summary	Gender	Sample Size	Number of Cases (Exposed Group)	Number of Cases (Unexposed Group)
					For Victims Of Sexual Assault						disease registries					
Hedtke	2008	USA	Prospective Cohort	Sexual	Self-Report or Self-Administered Survey	Lifetime	Anyone or Not-Specified	2	Past-Year Posttraumatic Stress Disorder (Ptsd) Wave 3	Incidence	self-report	18–91	Women	1302		
Elklit	2013	Denmark	Case-Control	Sexual	Routinely Collected/Administrative Data	Lifetime	Anyone or Not-Specified		Anxiety Disorder	Mortality	administrative medical records or disease registries	26 (13.41)	Women	2140	16	
Austin	2020	USA	Prospective Cohort	Sexual	Self-Report or Self-Administered Survey	Lifetime	Anyone or Not-Specified	9	Anxiety Diagnosis	Incidence	self-report	24–42	Women			
Austin	2020	USA	Prospective Cohort	Sexual	Self-Report or Self-Administered Survey	Lifetime	Anyone or Not-Specified	9	Anxiety Diagnosis	Incidence	self-report	24–42	Men			
Ali	2009	Sindh	Case-Control	Sexual	Self-Report or Self-Administered Survey	Lifetime	Partner		Depression	Mortality	self-report; physician diagnosis	15–48	Women	304	50	
Hedtke	2008	USA	Prospective Cohort	Sexual	Self-Report or Self-Administered Survey	Lifetime	Anyone or Not-Specified	2	Past-Year Major Depressive Episode (Mde) Wave 3	Incidence	self-report	18–91	Women	1302		
Benjet	2020	Mexico City	Prospective Cohort	Sexual	Self-Report or Self-Administered Survey	Lifetime	Anyone or Not-Specified	8	Major Depressive Disorder	Incidence	self-report	19–26	Combined Men/Women	964		
Austin	2020	USA	Prospective Cohort	Sexual	Self-Report or Self-Administered Survey	Lifetime	Anyone or Not-Specified	9	Depression Diagnosis	Incidence	self-report	24–42	Women			
Austin	2020	USA	Prospective Cohort	Sexual	Self-Report or Self-Administered Survey	Lifetime	Anyone or Not-Specified	9	Depression Diagnosis	Incidence	self-report	24–42	Men			
Gilbert	2012	New York	Prospective Cohort	Sexual	Self-Report or Self-Administered Survey	Past 6 Months At Wave 1	Partner; Former Partner	1	Use Of Hard Drugs (Crack/Cocaine And/Or Heroin)	Incidence	self-report	32.8 (10.08)	Women	241		
Nowotny	2013	USA	Prospective Cohort	Sexual	Self-Report or Self-Administered Survey	Reported At T1 Wave Iii (2001 - 2002)	Partner	7	Drug Use	Incidence	self-report	29	Women	2959		
Danielson	2009	USA	Prospective Cohort	Sexual	Self-Report or Self-Administered Survey	Lifetime	Anyone or Not-Specified	8	Drug Abuse	Incidence	self-report	20–26	Women	872		
Danielson	2009	USA	Prospective Cohort	Sexual	Self-Report or Self-Administered Survey	Lifetime	Anyone or Not-Specified	8	Drug Abuse	Incidence	self-report	20–26	Men	881		
Austin	2020	USA	Prospective Cohort	Sexual	Self-Report or Self-Administered Survey	Lifetime	Anyone or Not-Specified	9	Prescription Opioid Misuse	Incidence	self-report	24–42	Women			
Austin	2020	USA	Prospective Cohort	Sexual	Self-Report or Self-Administered Survey	Lifetime	Anyone or Not-Specified	9	Prescription Opioid Misuse	Incidence	self-report	24–42	Men			

Author	Year	Location	Study Design	Type of Violence	Exposure Assessment Method	Exposure Recall	Type of Perpetrator	Follow-up Years	Outcome	Type of Outcome	Outcome Assessment Method	Age Summary	Gender	Sample Size	Number of Cases (Exposed Group)	Number of Cases (Unexposed Group)
Maman	2002	United Republic Of Tanzania	Prospective Cohort	Sexual	Self-Report or Self-Administered Survey	Lifetime	Partner	0.25	HIV Positive	Incidence	biomarker	32	Women	245	44	
Quigley	2000	Uganda	Case-Control	Sexual	Self-Report or Self-Administered Survey	Past Year	Anyone or Not-Specified		HIV Infection	Mortality	biomarker	0–99	Women	122	13	30
Wyatt	2002	California	Case-Control	Sexual	Self-Report or Self-Administered Survey	Lifetime	Anyone or Not-Specified		HIV/AIDs	Mortality	biomarker	36.1	Women	457	155	144
Jewkes	2010	Eastern Cape	Prospective Cohort	Sexual	Self-Report or Self-Administered Survey	Lifetime	Non-Partner	2	HIV	Incidence	biomarker	16–23	Women		7	121
Abrahams	2021	Kwazulu-Natal	Case-Cohort	Sexual	Self-Report or Self-Administered Survey ; Attended Post-Rape Service Center	Lifetime	Anyone or Not-Specified	3	HIV	Incidence	biomarker	16–40	Women	845	37	49
Larsen	2016	Denmark	Prospective Cohort	Sexual	Routinely Collected/Administrative Data ; Attending Center For Victims Of Sexual Assault	Lifetime	Anyone or Not-Specified	9.7	Human Immunodeficiency Virus (HIV)	Incidence	administrative medical records or disease registries	12–93	Women	12505	19	2
Kouyoumdjian	2013	Uganda	Prospective Cohort	Sexual	Self-Report or Self-Administered Survey	Lifetime	Partner; Former Partner	5.5	HIV/Aids	Incidence	biomarker	15–49	Women		138	152
Birdthistle	2013	Uganda	Case-Control	Sexual	Self-Report or Self-Administered Survey	In The Last 12 Months or Since The Last Visit To The Clinic	Anyone or Not-Specified		HIV Incidence	Mortality	biomarker	14–81	Women	319	41	133
Burgueño	2017	Democratic Republic Of The Congo	Case-Control	Sexual	Self-Report or Self-Administered Survey	Lifetime	Anyone or Not-Specified		HIV Positivity	Mortality	biomarker	15–49	Combined Men/Women	1614	159	115
Deyessa	2018	Addis Ababa	Case-Control	Sexual	Self-Report or Self-Administered Survey	Lifetime	Partner; Former Partner		HIV	Mortality	biomarker	15–49	Women	510	52	
Deyessa	2018	Addis Ababa	Case-Control	Sexual	Self-Report or Self-Administered Survey	Lifetime	Stranger		HIV	Mortality	biomarker	15–49	Women	510		
Chowdhary	2008	Goa	Prospective Cohort	Sexual	Self-Report or Self-Administered Survey	Past 3 Months	Partner	1	Sexually Transmitted Infection (STI)	Incidence	self-report; biomarker	18–50	Women	1498		
Allsworth	2009	Rhode Island	Prospective Cohort	Sexual	Self-Report or Self-Administered Survey	Past Year	Anyone or Not-Specified	1.6	Incident STI	Incidence	administrative medical records or disease registries;	13–35	Women		5	56

Author	Year	Location	Study Design	Type of Violence	Exposure Assessment Method	Exposure Recall	Type of Perpetrator	Follow-up Years	Outcome	Type of Outcome	Outcome Assessment Method	Age Summary	Gender	Sample Size	Number of Cases (Exposed Group)	Number of Cases (Unexposed Group)
Weiss	2008	Goa, Rural	Prospective Cohort	Sexual	Self-Report or Self-Administered Survey	Lifetime	Partner	1	Sexually Transmitted Infection (STI)	Incidence	physician diagnosis; biomarker physician diagnosis; biomarker	18–45	Women	1552	6	
Larsen	2016	Denmark	Prospective Cohort	Sexual	Routinely Collected/Administrative Data ; Attending Center For Victims Of Sexual Assault	Lifetime	Anyone or Not-Specified	9.7	Sexually Transmitted Diseases	Incidence	administrative medical records or disease registries	12–93	Women	12505	136	179

Section 2: Sensitivity Analyses

Section 2.1: Sensitivity analyses results for GBV exposures and outcomes

Table S2. Sensitivity analysis results for GBV exposure and alcohol use disorder

Sensitivity analysis	% Trimming	Mean RR	95% UI for the mean RR without gamma	95% UI for the mean RR with gamma	BPRF	ROS	Star rating	Pub. bias	No. of studies (Obs.)	Selected bias covariates
Physical GBV										
Primary analysis	0%	1.51	1.21-1.89	0.92-2.48	1.00	-0.00	1	None	3 (5)	None
Women only	0%	1.46	0.95–2.25	0.48–4.42	0.58	N/A	0	None	3 (3)	None

Table S3. Sensitivity analysis results for GBV exposure and drug use disorders

Sensitivity analysis	% Trimming	Mean RR	95% UI for the mean RR without gamma	95% UI for the mean RR with gamma	BPRF	ROS	Star rating	Pub. bias	No. of studies (Obs.)	Selected bias covariates
Sexual violence										
Primary analysis	0%	1.69	1.22–2.36	0.66–4.33	0.77	-0.13	1	None	4 (6)	None
Women only	0%	1.75	1.17–2.61	0.53–5.72	0.64	-0.22	1	None	4 (4)	None
Psychological GBV										

Primary analysis	0%	1.49	0.96–2.30	0.43–5.09	0.53	NaN	0	None	3 (6)	None
Women only	0%	1.53	0.89–2.60	0.33–6.97	0.43	NaN	0	None	3 (4)	None
Physical GBV										
Primary analysis	0%	1.84	1.50–2.25	1.11–3.06	1.20	0.09	2	None	6 (8)	None
Women only	0%	1.93	1.25–2.97	0.45–8.26	0.57	-0.28	1	None	6 (6)	Exposure recall is not lifetime
Perpetrator-specific analysis	0%	1.89	1.53–2.33	1.13–3.15	1.23	0.1	2	None	5 (7)	None
Outcome definition limited to studies that focus on the use of non-marijuana illicit drugs	0%	1.74	1.34–2.25	0.94–3.21	1.04	0.02	2	None	3 (3)	None

Table S4. Sensitivity analysis results for GBV exposure and HIV/AIDS

**Sensitivity analysis**	**% Trimming**	**Mean RR**	**95% UI for the mean RR without gamma**	**95% UI for the mean RR with gamma**	**BPRF**	**ROS**	**Star rating**	**Pub. bias**	**No. of studies (Obs.)**	**Selected bias covariates**
Sexual violence										
Primary analysis	10%	1.85	1.42–2.39	0.73–4.68	0.85	-0.08	1	None	10 (11)	Unadjusted for age
	0%	2.28	1.52–3.41	0.44–11.83	0.57	-0.28	1	None	10 (11)	Unadjusted for age
Women only	10%	1.83	1.40–2.40	0.70–4.79	0.82	-0.1	1	None	10 (11)	Unadjusted for age
Perpetrator-specific analysis	0%	1.63	1.31–2.02	1.02–2.60	1.1	0.05	2	None	3 (3)	None
Any perpetrator-specific analysis	0%	3.16	1.39–7.15	0.17–57.44	0.28	-0.64	1	None	6 (6)	Unadjusted for age

Physical GBV										
Primary analysis	0%	1.65	1.37–1.98	1.08–2.52	1.15	0.072	2	None	4 (4)	None

Table S5. Sensitivity analysis results for GBV exposure and major depressive disorder

Sensitivity analysis	% Trimming	Mean RR	95% UI for the mean RR without gamma	95% UI for the mean RR with gamma	BPRF	ROS	Star rating	Pub. bias	No. of studies (Obs.)	Selected bias covariates
Sexual violence										
Primary analysis	0%	1.99	1.71–2.31	1.43–2.77	1.5	0.2	3	None	4 (5)	None
Women only	0%	2.06	1.61–2.65	1.13–3.78	1.24	0.11	2	None	3 (3)	None
Any perpetrator-specific analysis	0%	1.95	1.67–2.27	1.39–2.72	1.47	0.19	3	None	3 (4)	None
Psychological GBV										
Primary analysis	0%	1.66	1.06–2.60	0.46–5.95	0.57	-0.28	1	None	3 (5)	None
No SE adjustment applied	0%	1.74	1.06–2.84	0.42–7.24	0.52	-0.32	1	None	3 (5)	None
Women only	0%	1.71	1.13–2.59	0.53–5.52	0.64	-0.22	1	None	3 (4)	None
Physical GBV										
Primary analysis	0%	1.53	1.21–1.94	0.83–2.82	0.92	-0.04	1	None	4 (7)	Males included in the effect size
Women only	0%	1.70	1.33–2.17	0.92–3.14	1.01	0.01	2	None	4 (4)	None
Men only	0%	1.04	0.68–1.59	0.39–2.76	0.46	N/A	0	None	3 (3)	None

Perpetrator-specific analysis	0%	1.48	1.16–1.89	0.81–2.72	0.89	-0.06	1	None	3 (6)	None
Outcome sensitivity analysis – definition limited to 'major depressive disorder'	0%	1.43	1.09–1.86	0.77–2.64	0.85	-0.08	1	None	3 (5)	None

Table S6. Sensitivity analysis results for GBV exposure and anxiety disorders

Sensitivity analysis	% Trimming	Mean RR	95% UI for the mean RR without gamma	95% UI for the mean RR with gamma	BPRF	ROS	Star rating	Pub. bias	No. of studies (Obs.)	Selected bias covariates
Sexual violence										
Primary analysis	0%	2.86	1.66–4.92	0.61–13.34	0.78	-0.12	1	None	3 (4)	None
Women only	0%	2.89	1.72–4.86	0.67–12.46	0.85	-0.08	1	None	3 (3)	None
Physical GBV										
Primary analysis	0%	1.82	1.19–2.77	0.60–5.51	0.72	-0.2	1	None	3 (7)	Outcome defined as PTSD
No adjustment applied	0%	1.97	1.25–3.10	0.56–6.92	0.68	-0.19	1	No	3 (7)	Outcome defined as PTSD
Women only	0%	1.78	1.32–2.38	0.94–3.37	1.04	0.02	2	No	3 (4)	None

Table S7. Sensitivity analysis results for GBV exposure and maternal abortion and miscarriage

Sensitivity analysis	% Trimming	Mean RR	95% UI for the mean RR	95% UI for the mean RR	BPRF	ROS	Star rating	Pub. bias	No. of studies (Obs.)	Selected bias covariates

			without gamma	with gamma						
Sexual violence										
Primary analysis	0%	2.53	2.20–2.91	1.91–3.35	2	0.35	3	None	5 (6)	None
No SE adjustment applied	0%	2.51	2.27–2.77	2.06–3.06	2.12	0.38	3	None	5 (6)	None
No pregnancy recall	0%	2.54	2.21–2.92	1.92–3.36	2.01	0.35	3	None	4 (5)	None
Perpetrator-specific analysis	0%	2.99	1.80–4.97	0.82–10.91	1.01	0	2	None	4 (4)	None
Outcome sensitivity analysis (limited to cases of induced abortion)	0%	2.75	2.46–3.07	2.21–3.41	2.29	0.41	4	None	3 (3)	None
Outcome sensitivity analysis (limited to cases of miscarriage)	0%	1.64	1.30–2.08	1.01–2.68	1.09	0.04	2	None	3 (3)	None
Psychological GBV										
Primary analysis	0%	1.34	0.91–1.96	0.38–4.70	0.47	N/A	0	None	6 (7)	Unadjusted for age, Unadjusted for age, sex, and at least one other confounding variable
Perpetrator-specific analysis	0%	1.34	0.91–1.98	0.37–4.82	0.46	N/A	0	None	6 (7)	Unadjusted for age, Unadjusted for age, sex, and at least one other confounding variable, Violence exposure is defined as a component of reference definition
Outcome sensitivity analysis (limited to cases of induced abortion)	0%	1.81	1.25–2.62	0.80–4.08	0.92	-0.04	1	None	3 (4)	None
Outcome sensitivity analysis (limited to cases of miscarriage)	0%	1.07	0.65–1.77	0.27–4.31	0.33	N/A	0	None	3 (3)	None
Physical GBV										
Primary analysis	0%	2.76	1.74–4.38	0.48–15.99	0.63	-0.23	1	None	8 (8)	Unadjusted for age, sex, and at least one other confounding variable, Outcome is defined as induced abortion
No pregnancy recall	0%	1.95	1.28–2.97	0.51–7.46	0.63	-0.23	1	None	5 (5)	Exposure recall is not lifetime, Outcome is defined as induced abortion
Perpetrator-specific analysis	0%	2.55	1.49–4.38	0.31–20.81	0.44	-0.41	1	None	8 (8)	Unadjusted for age, sex, and at least one other confounding variable, Outcome is defined as induced abortion

Outcome sensitivity analysis (limited to cases of induced abortion)	0%	2.71	1.47–4.98	0.55–13.24	0.72	-0.17	1	None	3 (3)	None
Outcome sensitivity analysis (limited to cases of miscarriage)	0%	2.76	1.49–5.11	0.35–21.88	0.49	-0.36	1	None	5 (5)	Exposure during pregnancy, Unadjusted for age

Table S8. Sensitivity analysis results for GBV exposure and self-harm

**Sensitivity analysis**	**% Trimming**	**Mean RR**	**95% UI for the mean RR without gamma**	**95% UI for the mean RR with gamma**	**BPRF**	**ROS**	**Star rating**	**Pub. bias**	**No. of studies (Obs.)**	**Selected bias covariates**
Physical GBV										
Primary analysis	0%	3.17	0.99–10.12	0.13–79.57	0.21	NaN	0	None	3 (3)	None
Psychological GBV										
Primary analysis	0%	1.56	0.94–2.61	0.48–5.07	0.58	NaN	0	None	3 (4)	None
Women only	0%	1.64	0.87–3.10	0.34–7.93	0.44	NaN	0	None	3 (3)	None

Section 2.2: Sensitivity analyses forest plots for physical GBV and the corresponding outcomes

Figure S1. Sensitivity analysis results for physical GBV exposure (males only) and outcomes

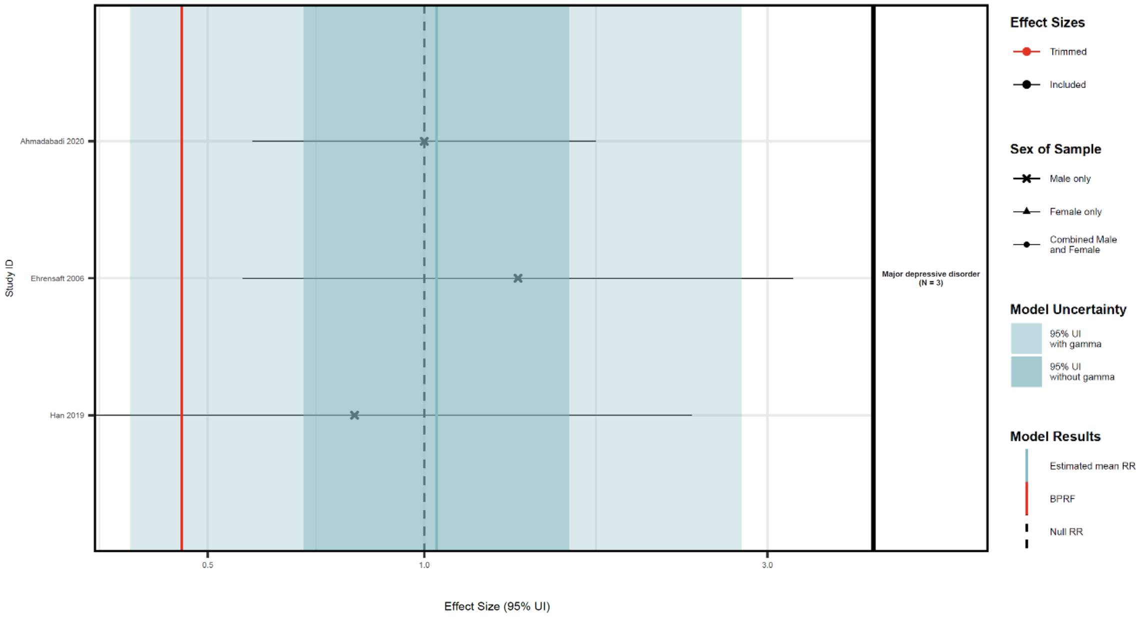

Figure S2. Sensitivity analysis results for physical GBV exposure (females only) and outcomes

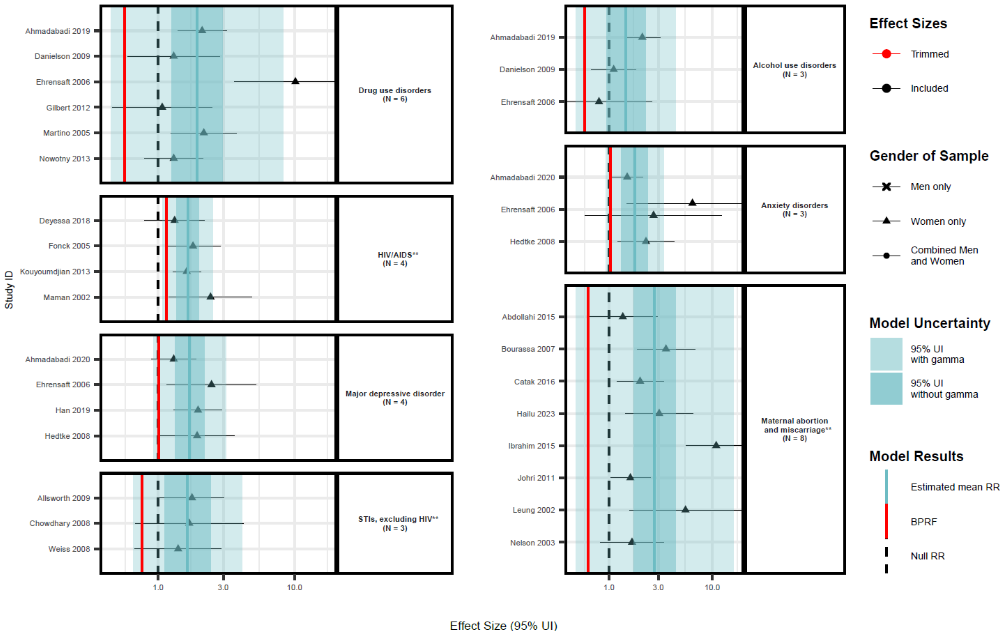

Figure S3. Sensitivity analysis results for physical GBV exposure (non-pregnancy recall) and outcomes

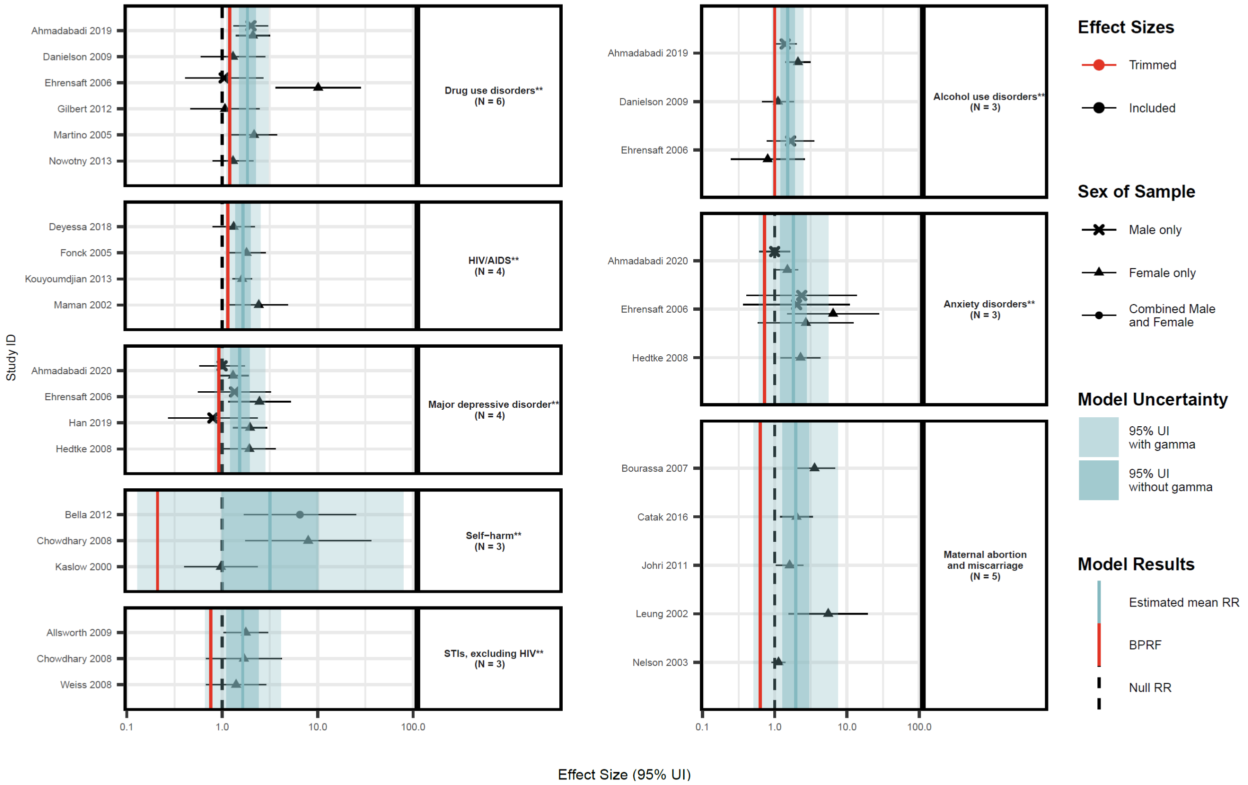

Figure S4. Sensitivity analysis results for physical GBV exposure (pregnancy-specific recall) and outcomes

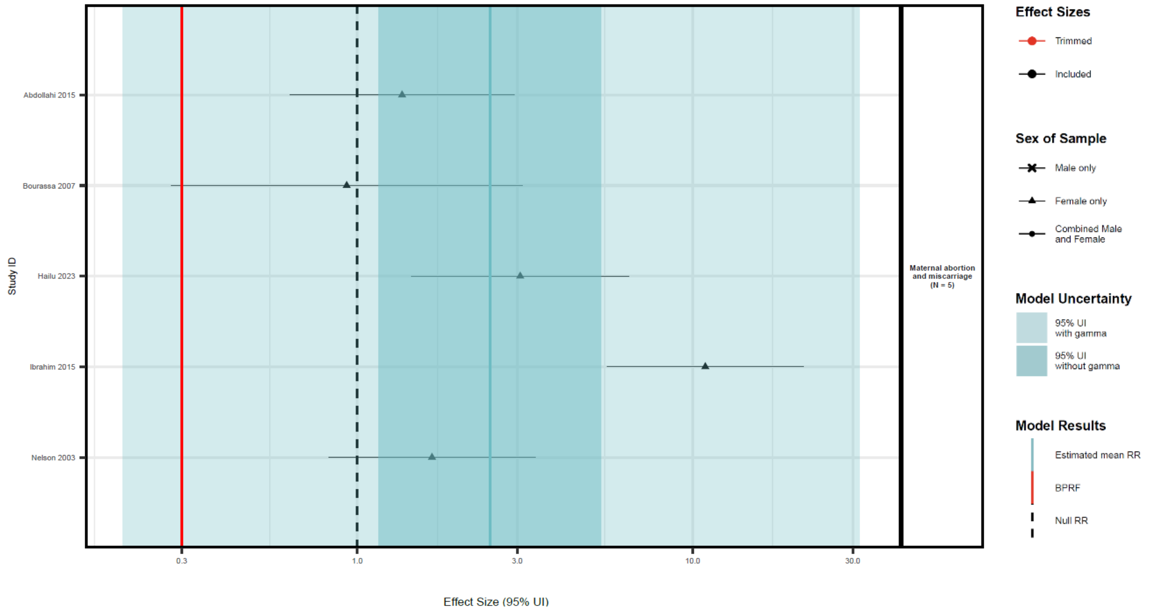

Figure S5. Sensitivity analysis results for perpetrator-specific physical GBV exposure and outcomes

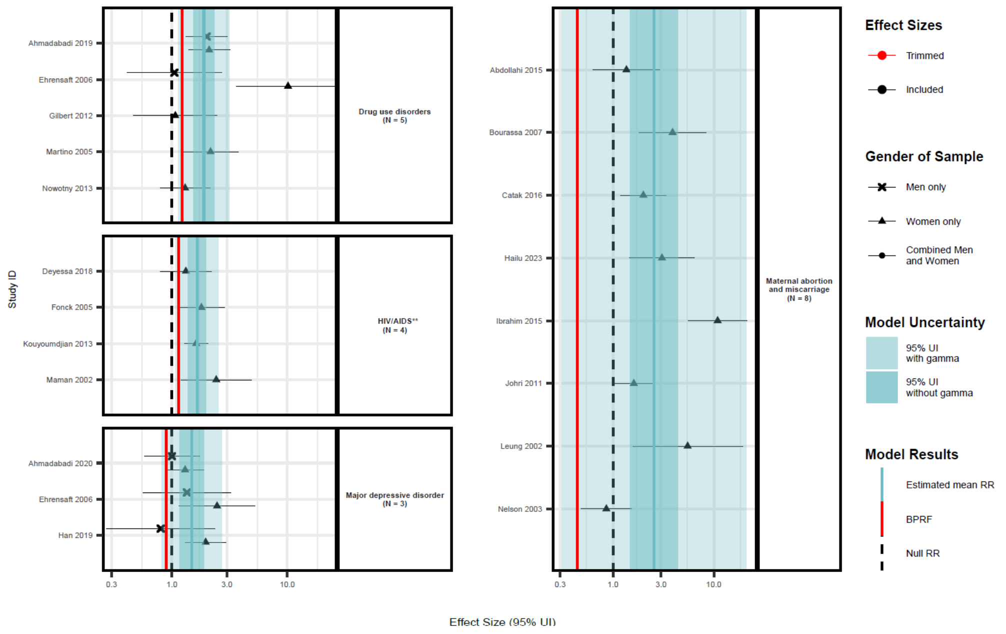

Figure S6. Sensitivity analysis results for physical GBV exposure and alternative outcome definitions

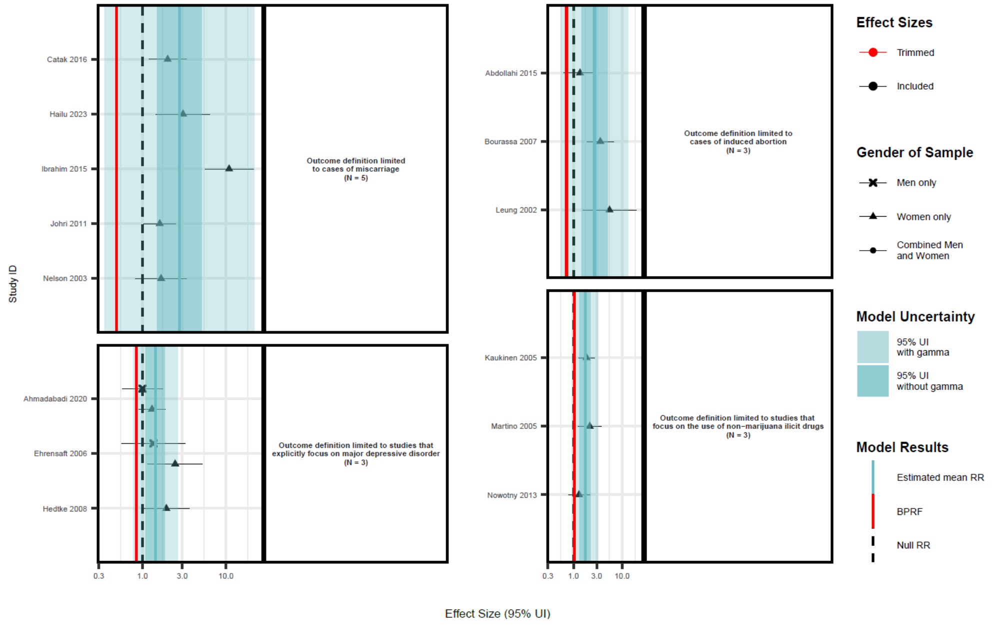

Figure S7. Sensitivity analysis results for physical GBV exposure (no adjustment) and outcomes

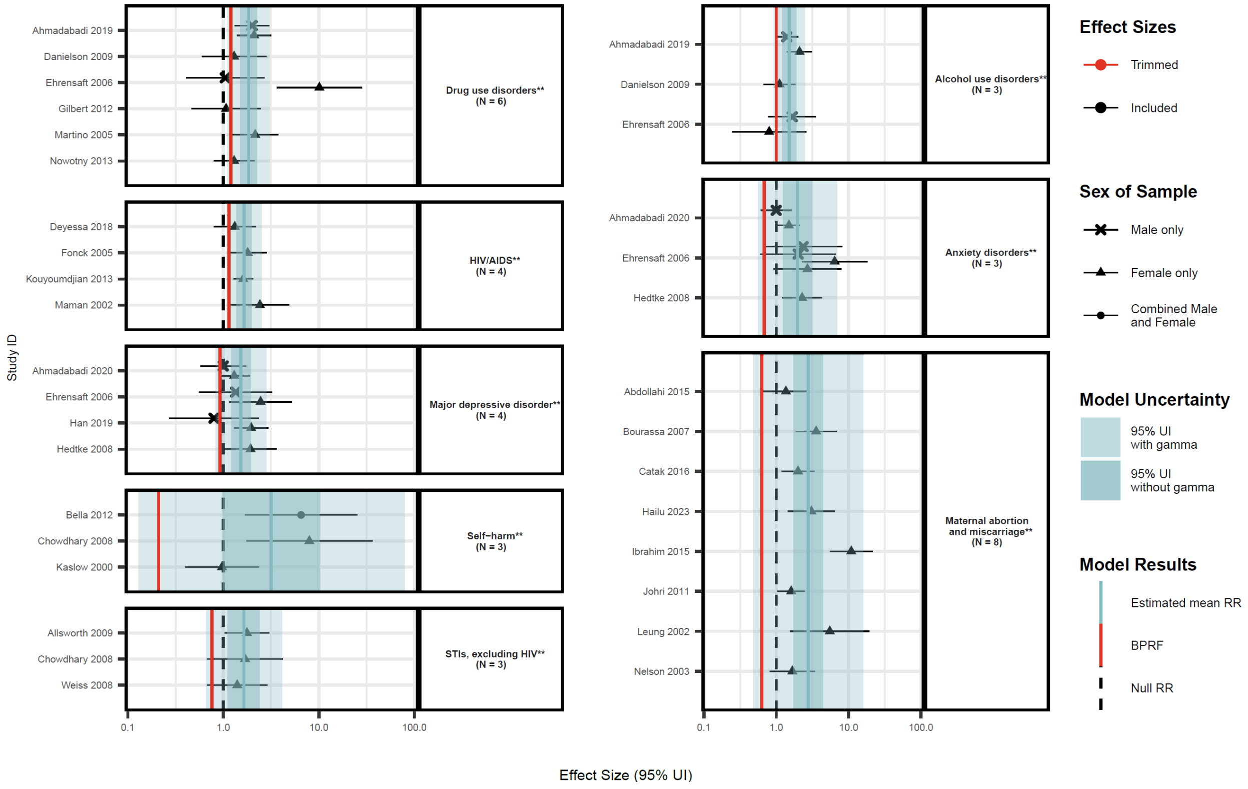

Figure S8. Sensitivity analysis results for physical GBV exposure (no trimming) and outcomes

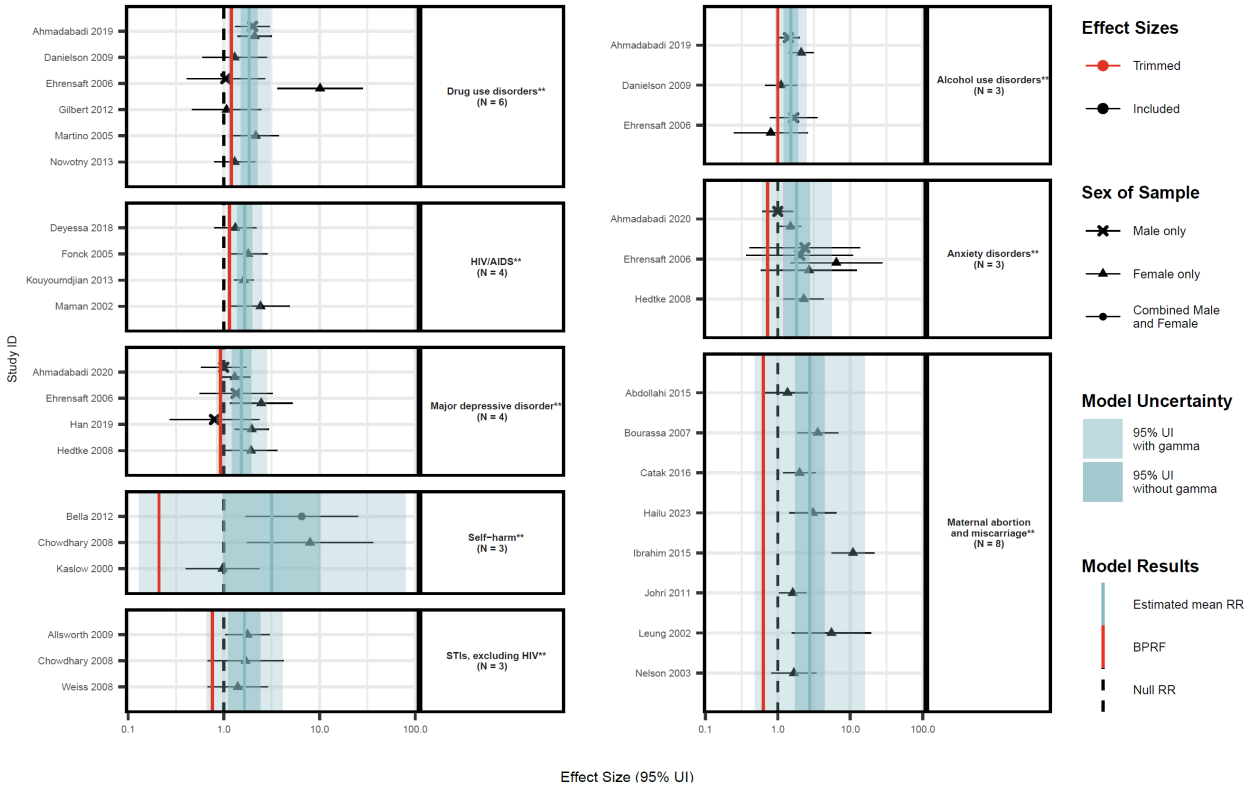

Section 2.3: Sensitivity analyses forest plots for sexual violence exposures and the corresponding outcomes

Figure S9. Sensitivity analysis results for perpetrator-specific sexual violence exposure and outcomes

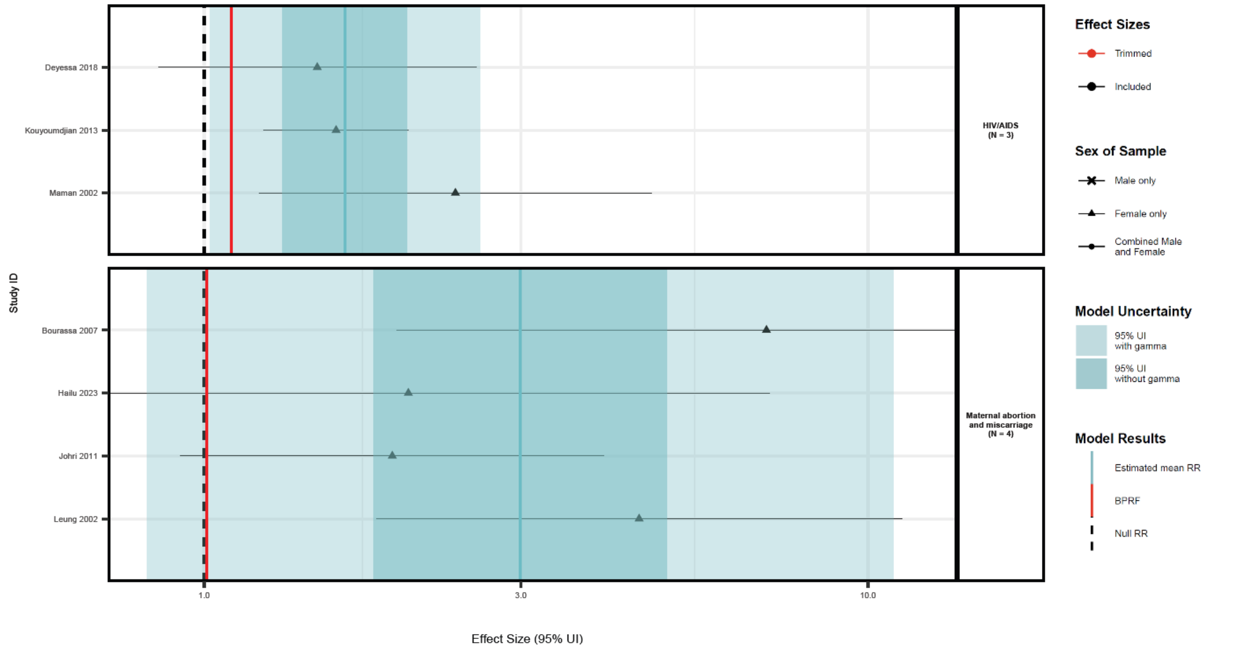

Figure S10. Sensitivity analysis results for sexual violence exposure and alternative outcome definitions

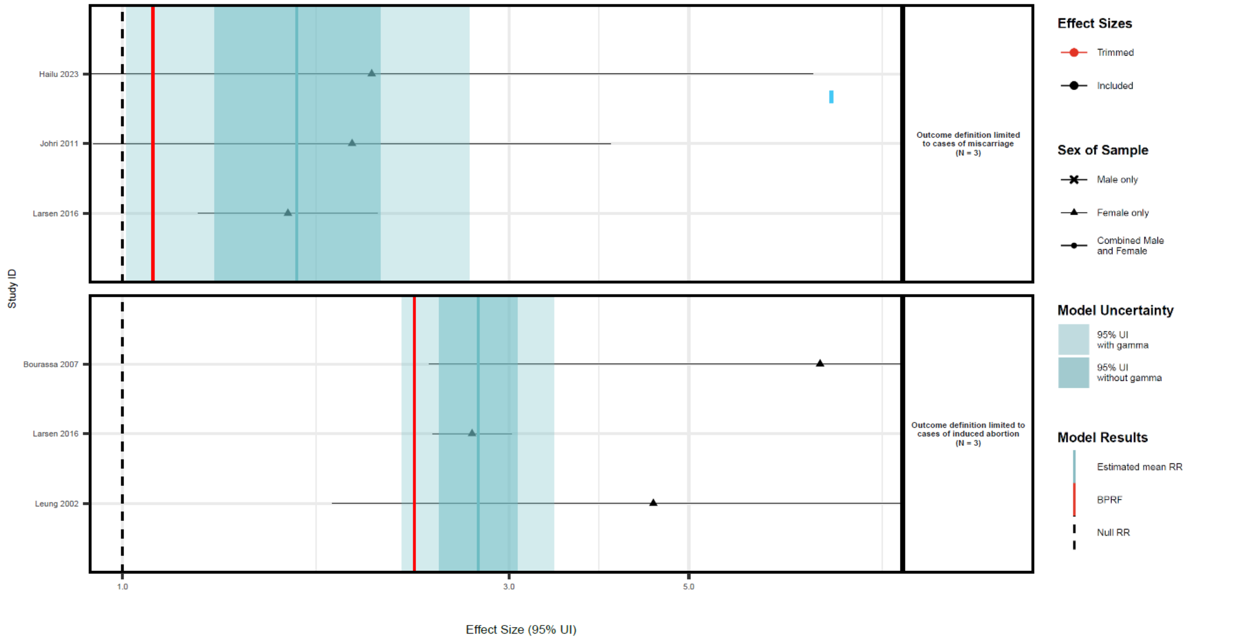

Figure S11. Sensitivity analysis results for sexual violence exposure (females only) and outcomes

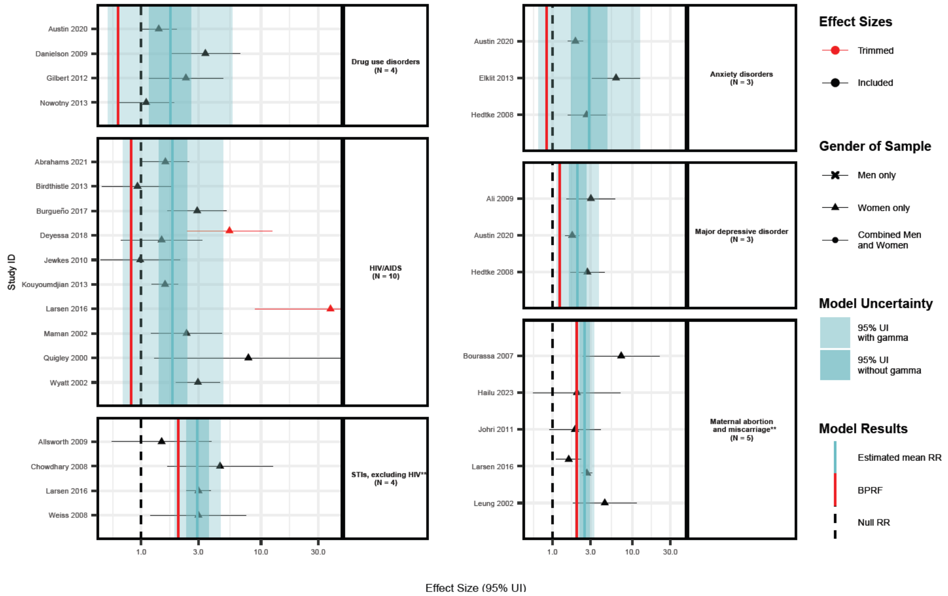

Figure S12. Sensitivity analysis results for sexual violence exposure (non-pregnancy recall) and outcomes

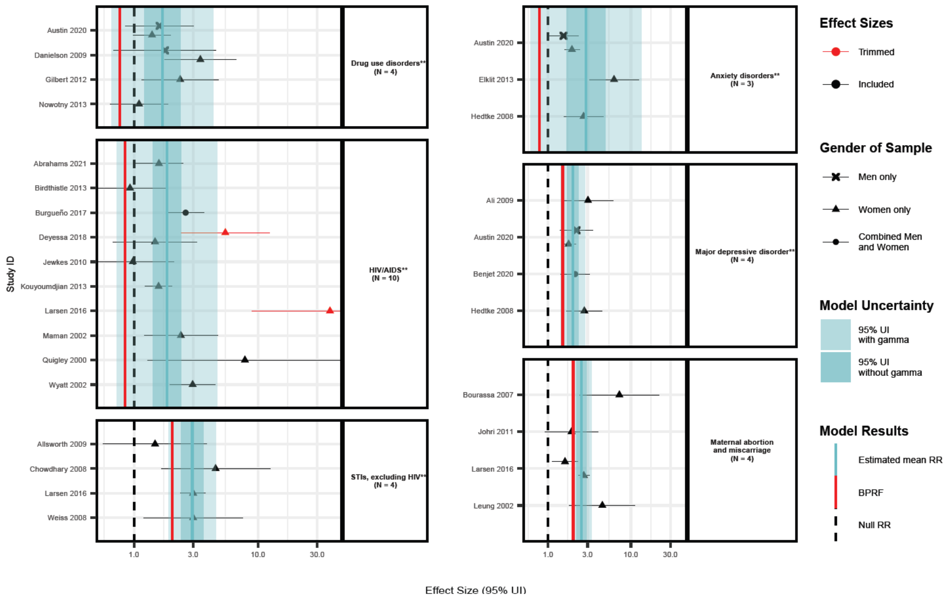

Figure S13. Sensitivity analysis results for sexual violence exposure by any perpetrator and outcomes

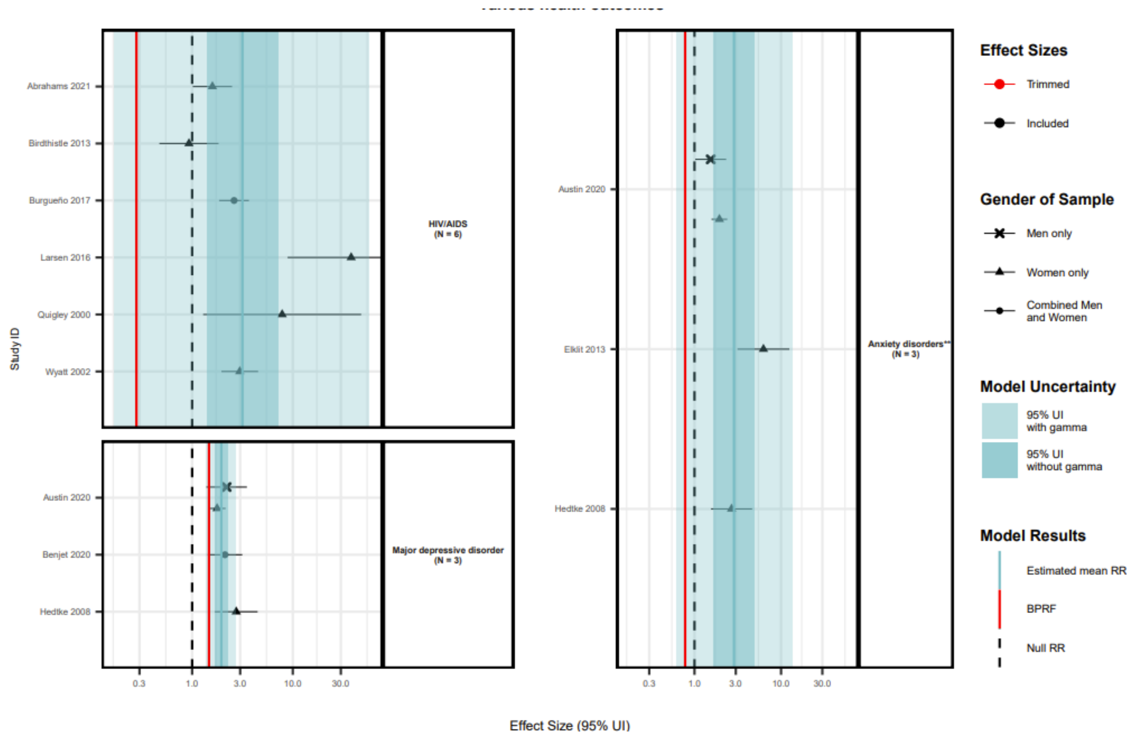

Figure S14. Sensitivity analysis results for sexual violence exposure (no adjustment) and outcomes

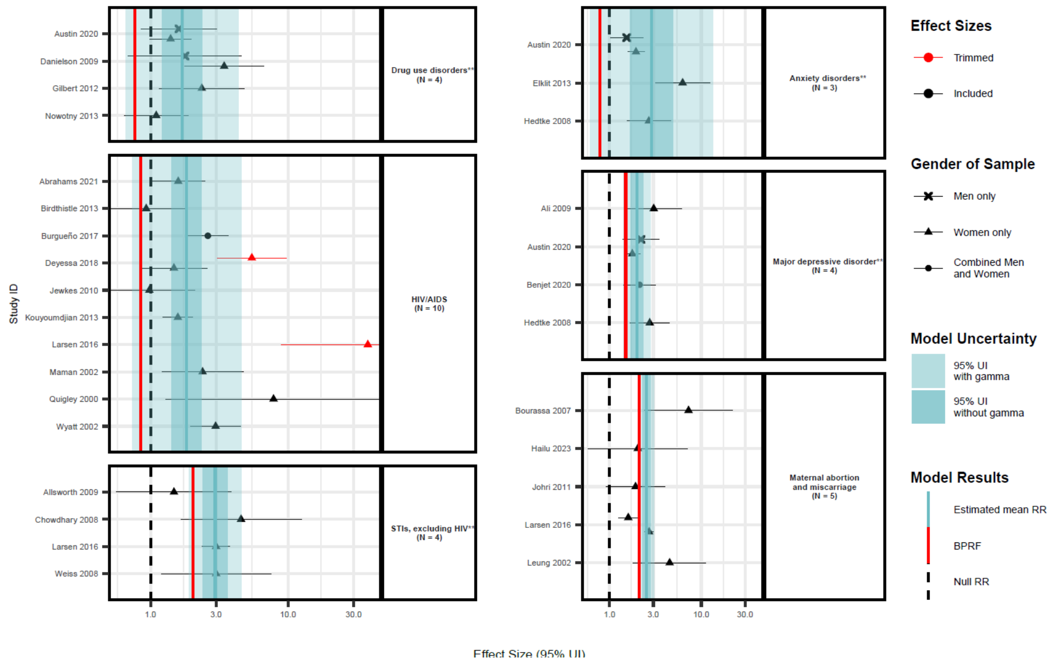

Figure S15. Sensitivity analysis results for sexual violence exposure (no trimming) and outcomes

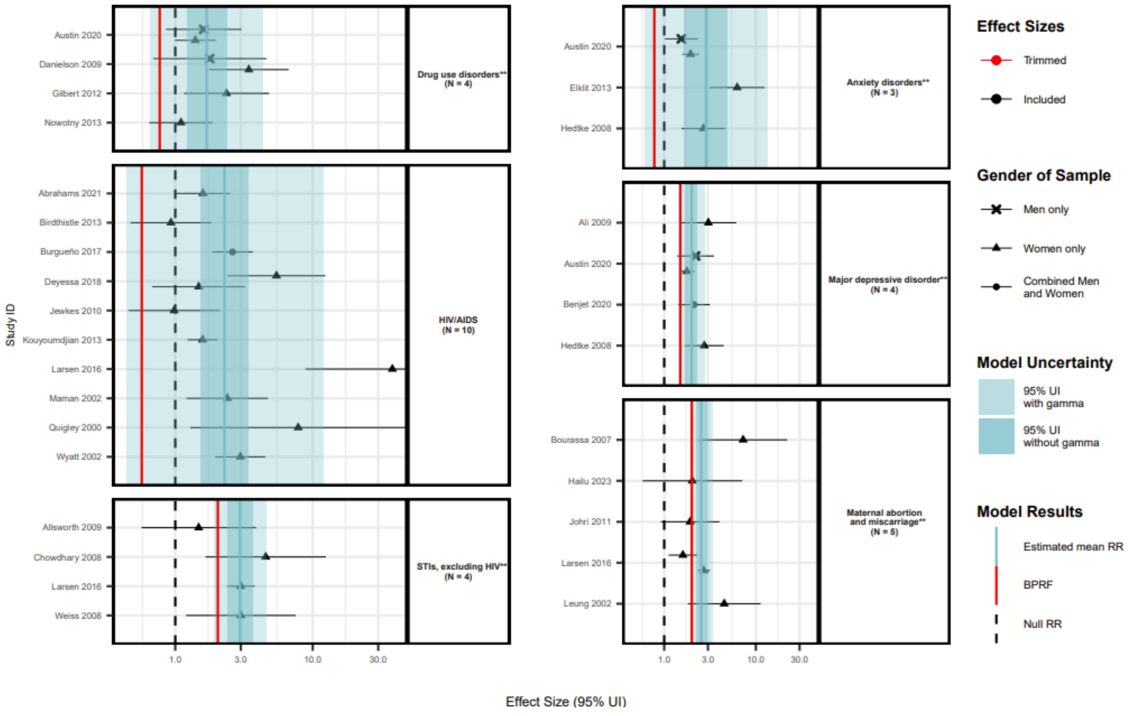

Section 2.4: Sensitivity analyses forest plots for psychological GBV and the corresponding outcomes

Figure S16. Sensitivity analysis results for psychological GBV exposure and alternative outcome definitions

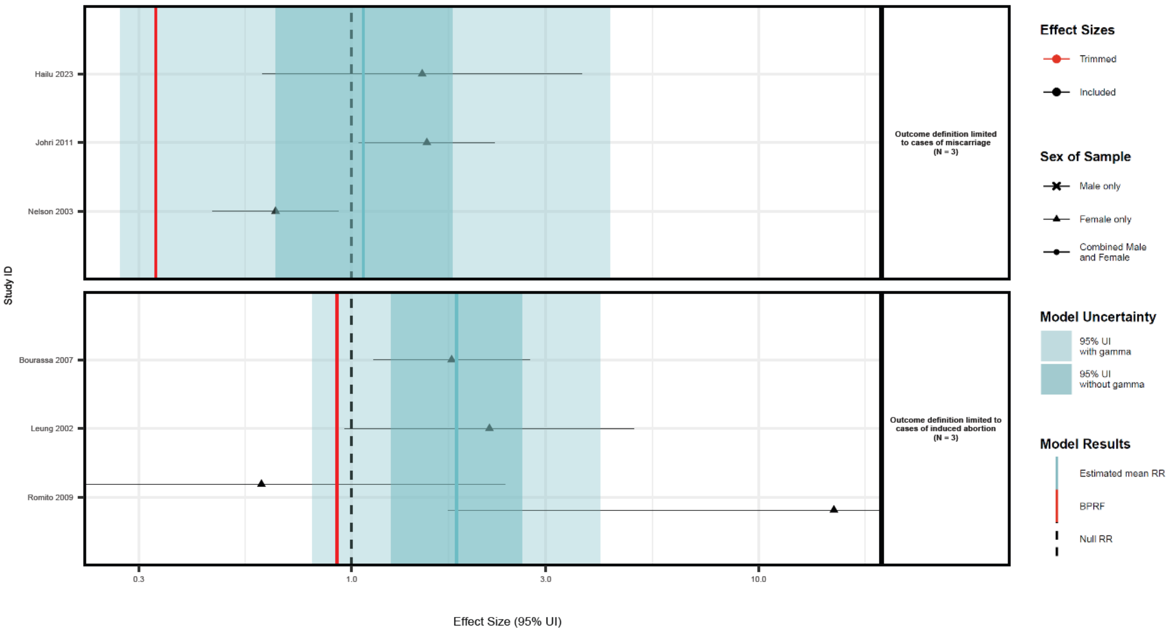

Figure S17. Sensitivity analysis results for psychological GBV exposure (females only) and outcomes

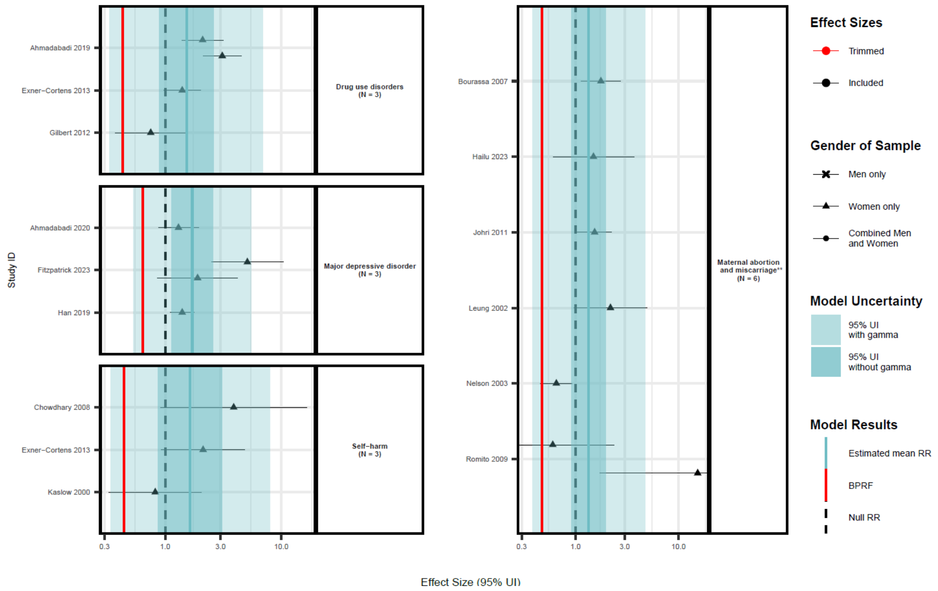

Figure S18. Sensitivity analysis results for psychological GBV exposure (non-pregnancy recall) and outcomes

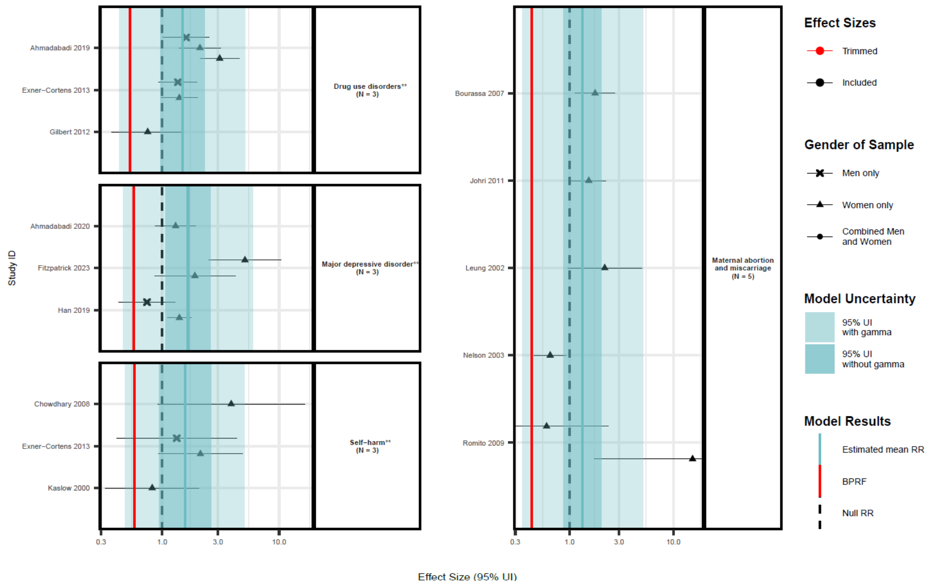

Figure S19. Sensitivity analysis results for perpetrator-specific psychological GBV exposure and outcomes

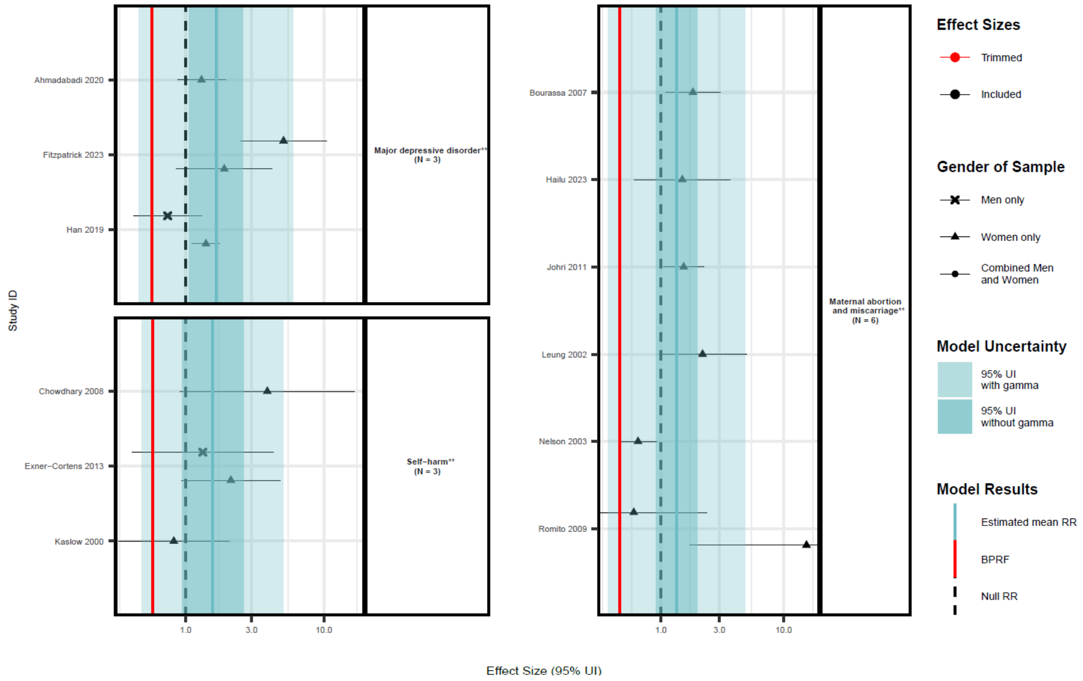

Figure S20. Sensitivity analysis results for psychological GBV exposure (no adjustments) and definitions

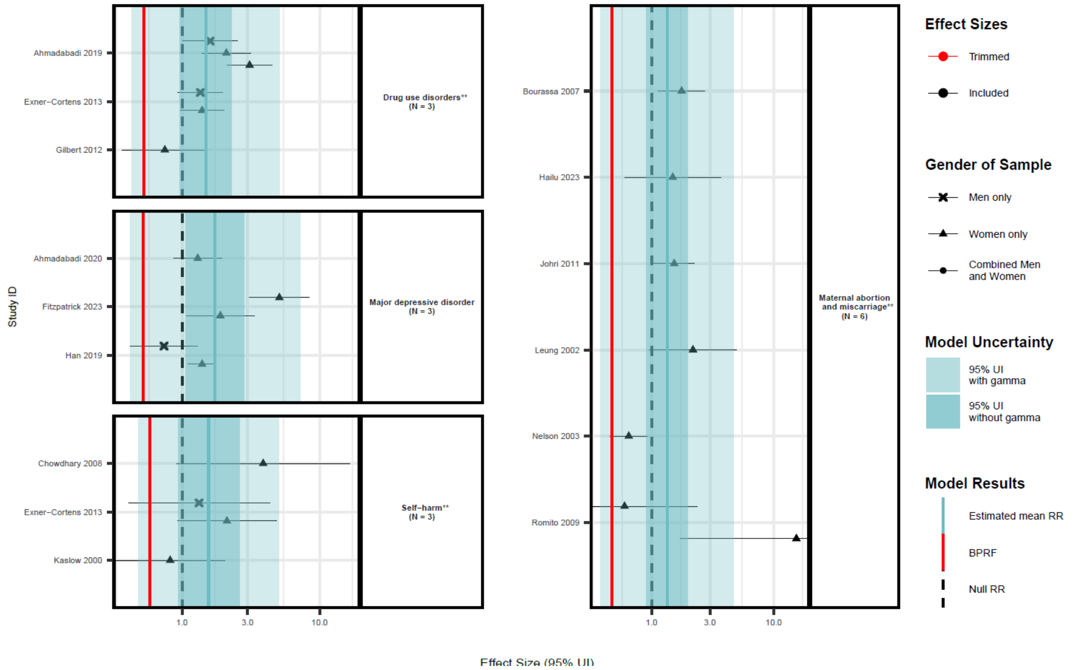

Figure S21. Sensitivity analysis results for psychological GBV exposure (no trimming) and definitions

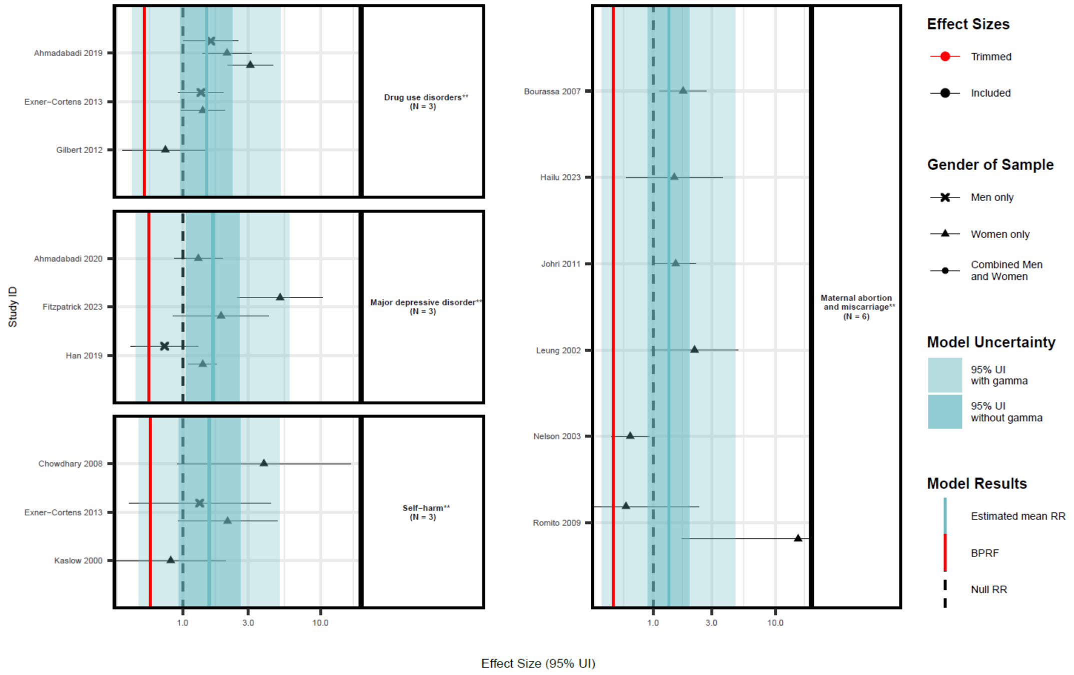

Section 3: PRISMA and GATHER Checklists

Section 3.1: GATHER

Table S9. GATHER checklist

Item #	Checklist item	Reported on page #
Objectives and funding		
1	Define the indicator(s), populations (including age, sex, and geographic entities), and time period(s) for which estimates were made.	Main text Methods overview
2	List the funding sources for the work.	Main text Acknowledgements
Data Inputs		
For all data inputs from multiple sources that are synthesized as part of the study:		
3	Describe how the data were identified and how the data were accessed.	Main text Methods overview
4	Specify the inclusion and exclusion criteria. Identify all ad-hoc exclusions.	Methods; Supplementary Information Section 4.2
5	Provide information on all included data sources and their main characteristics. For each data source used, report reference information or contact name/institution, population represented, data collection method, year(s) of data collection, sex and age range, diagnostic criteria or measurement method, and sample size, as relevant.	Supplementary Information Table S1
6	Identify and describe any categories of input data that have potentially important biases (e.g., based on characteristics listed in item 5).	Supplementary Information Section 5
For data inputs that contribute to the analysis but were not synthesized as part of the study:		
7	Describe and give sources for any other data inputs.	N/A
For all data inputs:		
8	Provide all data inputs in a file format from which data can be efficiently extracted (e.g., a spreadsheet rather than a PDF), including all relevant meta-data listed in item 5. For any data inputs that cannot be shared because of ethical or legal reasons, such as third-party ownership, provide a contact name or the name of the institution that retains the right to the data.	Supplementary Information Section 1.2; Supplementary Information Section 8
Data analysis		
9	Provide a conceptual overview of the data analysis method. A diagram may be helpful.	Main text Methods
10	Provide a detailed description of all steps of the analysis, including mathematical formulae. This description should cover, as relevant, data cleaning, data pre-processing, data adjustments and weighting of data sources, and mathematical or statistical model(s).	Main text Methods
11	Describe how candidate models were evaluated and how the final model(s) were selected.	Main text Methods “Model validation” section
12	Provide the results of an evaluation of model performance, if done, as well as the results of any relevant sensitivity analysis.	Main text Methods “Model validation” section; Supplementary Information section 2
13	Describe methods for calculating uncertainty of the estimates. State which sources of uncertainty were, and were not, accounted for in the uncertainty analysis.	Main text Methods “Quantifying between-study heterogeneity” section
14	State how analytic or statistical source code used to generate estimates can be accessed.	Code availability
Results and Discussion		
15	Provide published estimates in a file format from which data can be efficiently extracted.	Table 2
16	Report a quantitative measure of the uncertainty of the estimates (e.g. uncertainty intervals).	Main text Results; Table 2
17	Interpret results in light of existing evidence. If updating a previous set of estimates, describe the reasons for changes in estimates.	Main text Discussion
18	Discuss limitations of the estimates. Include a discussion of any modelling assumptions or data limitations that affect interpretation of the estimates.	Main text Discussion

Section 3.2: PRISMA

Table S10. PRISMA 2020 abstract checklist

Section and Topic	Item #	Checklist item	Reported (Yes/No)

**TITLE**			
Title	1	Identify the report as a systematic review.	Yes.
**BACKGROUND**			
Objectives	2	Provide an explicit statement of the main objective(s) or question(s) the review addresses.	Yes.
**METHODS**			
Eligibility criteria	3	Specify the inclusion and exclusion criteria for the review.	Not in abstract. Covered in detail in the main text and supplementary information.
Information sources	4	Specify the information sources (e.g. databases, registers) used to identify studies and the date when each was last searched.	Not in abstract. Covered in detail in the main text and supplementary information.
Risk of bias	5	Specify the methods used to assess risk of bias in the included studies.	Not in abstract. Covered in detail in the main text and supplementary information.
Synthesis of results	6	Specify the methods used to present and synthesise results.	Yes, briefly. More detail provided in the main text and supplementary information.
**RESULTS**			
Included studies	7	Give the total number of included studies and participants and summarise relevant characteristics of studies.	Not in abstract. Covered in detail in the main text and supplementary information.
Synthesis of results	8	Present results for main outcomes, preferably indicating the number of included studies and participants for each. If meta-analysis was done, report the summary estimate and confidence/credible interval. If comparing groups, indicate the direction of the effect (i.e. which group is favoured).	Yes, briefly. More detail provided in the main text and supplementary information.
**DISCUSSION**			
Limitations of evidence	9	Provide a brief summary of the limitations of the evidence included in the review (e.g. study risk of bias, inconsistency and imprecision).	Not in abstract. Covered in detail in the main text and supplementary information.
Interpretation	10	Provide a general interpretation of the results and important implications.	Yes.
**OTHER**			
Funding	11	Specify the primary source of funding for the review.	Not in abstract but described in the main text.
Registration	12	Provide the register name and registration number.	Not in abstract but reported in main text methods section.

Table S11. PRISMA 2020 checklist

Section and Topic	Item #	Checklist item	Location where item is reported
**TITLE**			
Title	1	Identify the report as a systematic review.	Manuscript Title and Methods Section Headings
**ABSTRACT**			
Abstract	2	See the PRISMA 2020 for Abstracts checklist.	Abstract section in main text
**INTRODUCTION**			
Rationale	3	Describe the rationale for the review in the context of existing knowledge.	Main section in-text
Objectives	4	Provide an explicit statement of the objective(s) or question(s) the review addresses.	Main section in-text
**METHODS**			
Eligibility criteria	5	Specify the inclusion and exclusion criteria for the review and how studies were grouped for the syntheses.	"Systematic Review and meta-analysis" section in Methods & Section 4.2: Inclusion and Exclusion Criteria in Supplementary Information
Information sources	6	Specify all databases, registers, websites, organisations, reference lists and other sources searched or consulted to identify studies. Specify the date when each source was last searched or consulted.	"Systematic Review and meta-analysis" section in Methods & Section 4.1: Literature searches in Supplementary Information
Search strategy	7	Present the full search strategies for all databases, registers and websites, including any filters and limits used.	"Systematic Review and meta-analysis" section in Methods & Section 4.1: Literature searches in Supplementary Information
Selection process	8	Specify the methods used to decide whether a study met the inclusion criteria of the review, including how many reviewers screened each record and each report retrieved, whether they worked independently, and if applicable, details of automation tools used in the process.	"Systematic Review and meta-analysis" section in Methods main text
Data collection process	9	Specify the methods used to collect data from reports, including how many reviewers collected data from each report, whether they worked independently, any processes for obtaining or confirming data from study investigators, and if applicable, details of automation tools used in the process.	"Systematic Review and meta-analysis" section in Methods main text
Data items	10a	List and define all outcomes for which data were sought. Specify whether all results that were compatible with each outcome domain in each study were sought (e.g. for all measures, time points, analyses), and if not, the methods used to decide which results to collect.	"Data selection of gender-based sexual, physical, and psychological violence and its health outcomes" section in Methods main text; Supplementary Information Section 4.5: Exposure and outcome definitions; Table S16: Definitions of included risk factors; Table S17: Summarized exposure definitions for gender-

			based violence used in included studies & Table S18: Definitions of included outcomes
	10b	List and define all other variables for which data were sought (e.g. participant and intervention characteristics, funding sources). Describe any assumptions made about any missing or unclear information.	Section 4.4; Data Extraction in Supplementary Information
Study risk of bias assessment	11	Specify the methods used to assess risk of bias in the included studies, including details of the tool(s) used, how many reviewers assessed each study and whether they worked independently, and if applicable, details of automation tools used in the process.	"Testing and adjusting for biases across study designs and characteristics" in Methods section; Supplementary Information Section 5.1: Definitions of bias covariates
Effect measures	12	Specify for each outcome the effect measure(s) (e.g. risk ratio, mean difference) used in the synthesis or presentation of results.	Section 1.2: Summary characteristics of included studies in Supplementary Information
Synthesis methods	13a	Describe the processes used to decide which studies were eligible for each synthesis (e.g. tabulating the study intervention characteristics and comparing against the planned groups for each synthesis (item #5)).	"Data selection of sexual, physical, and psychological violence and its health outcomes" in Methods main text
	13b	Describe any methods required to prepare the data for presentation or synthesis, such as handling of missing summary statistics, or data conversions.	Methods main text; Section 1.2 in Supplementary Information
	13c	Describe any methods used to tabulate or visually display results of individual studies and syntheses.	Methods main text; Sections 1.2 & 5 in Supplementary Information
	13d	Describe any methods used to synthesize results and provide a rationale for the choice(s). If meta-analysis was performed, describe the model(s), method(s) to identify the presence and extent of statistical heterogeneity, and software package(s) used.	"Estimating the burden of proof risk function" methods main text; Sections 5 & 8 in Supplementary Information
	13e	Describe any methods used to explore possible causes of heterogeneity among study results (e.g. subgroup analysis, meta-regression).	"Quantifying between-study heterogeneity" methods main text; Supplementary Information Section 6: Sensitivity Analyses and Supplementary Results
	13f	Describe any sensitivity analyses conducted to assess robustness of the synthesized results.	Results section in main text; Supplementary Information Section 2: Sensitivity Analyses
Reporting bias assessment	14	Describe any methods used to assess risk of bias due to missing results in a synthesis (arising from reporting biases).	"Testing and adjusting for biases across study designs and characteristics" in Methods main text; Section 5: Study Quality and Bias Assessment
Certainty	15	Describe any methods used to assess certainty (or confidence) in the body of evidence	"Estimating the burden

assessment		for an outcome.	of proof risk function" in Methods main text
**RESULTS**			
Study selection	16a	Describe the results of the search and selection process, from the number of records identified in the search to the number of studies included in the review, ideally using a flow diagram.	PRISMA flow diagram (Section 1 in Supplementary Information); "Data selection of gender-based sexual, physical, and psychological violence and its health outcomes" in methods main text
	16b	Cite studies that might appear to meet the inclusion criteria, but which were excluded, and explain why they were excluded.	PRISMA flow diagram (Section 1 in Supplementary Information)
Study characteristics	17	Cite each included study and present its characteristics.	Table S1: Effect size data details for sexual, physical, and psychological GBV (Section 1 in Supplementary Information)
Risk of bias in studies	18	Present assessments of risk of bias for each included study.	Section 5.2: Bias covariates for each risk-outcome pair in Supplementary Information
Results of individual studies	19	For all outcomes, present, for each study: (a) summary statistics for each group (where appropriate) and (b) an effect estimate and its precision (e.g. confidence/credible interval), ideally using structured tables or plots.	Results section in main text; Table 2; Figures 2–4
Results of syntheses	20a	For each synthesis, briefly summarise the characteristics and risk of bias among contributing studies.	Section 1.2: Summary characteristics of included studies in Supplementary Information; Section 5: Study Quality and Bias Assessment in Supplementary Information
	20b	Present results of all statistical syntheses conducted. If meta-analysis was done, present for each the summary estimate and its precision (e.g. confidence/credible interval) and measures of statistical heterogeneity. If comparing groups, describe the direction of the effect.	Results section in main text; Table 2; Figures 2–4
	20c	Present results of all investigations of possible causes of heterogeneity among study results.	Section 2: Sensitivity Analyses in Supplementary Information
	20d	Present results of all sensitivity analyses conducted to assess the robustness of the synthesized results.	Section 2: Sensitivity Analyses in Supplementary Information
Reporting biases	21	Present assessments of risk of bias due to missing results (arising from reporting biases) for each synthesis assessed.	Results section main text; Section 5 in Supplementary Information
Certainty of evidence	22	Present assessments of certainty (or confidence) in the body of evidence for each outcome assessed.	Results section main text

**DISCUSSION**			
Discussion	23a	Provide a general interpretation of the results in the context of other evidence.	Discussion section main text
	23b	Discuss any limitations of the evidence included in the review.	Discussion section main text
	23c	Discuss any limitations of the review processes used.	Discussion section main text
	23d	Discuss implications of the results for practice, policy, and future research.	Discussion section main text
**OTHER INFORMATION**			
Registration and protocol	24a	Provide registration information for the review, including register name and registration number, or state that the review was not registered.	The systematic review was registered in PROSPERO (CRD42022299831); The entirety of the Global Burden of Diseases, Injuries, and Risk Factors Study has been registered and approved through the UW IRB, as detailed in Methods section main text.
	24b	Indicate where the review protocol can be accessed, or state that a protocol was not prepared.	Review protocol publication cited in Methods section main text; review was registered in PROSPERO (CRD42022299831)
	24c	Describe and explain any amendments to information provided at registration or in the protocol.	NA
Support	25	Describe sources of financial or non-financial support for the review, and the role of the funders or sponsors in the review.	Main text "Acknowledgments"
Competing interests	26	Declare any competing interests of review authors.	Main text "Competing interests"
Availability of data, code and other materials	27	Report which of the following are publicly available and where they can be found; template data collection forms; data extracted from included studies; data used for all analyses; analytic code; any other materials used in the review.	Main text "Data availability" and "Code availability" sections; data collection form template in Table S15: Data extraction template in Supplementary Information

Section 4: Data source identification and assessment

The following systematic review methodology was adapted from the "Supplementary Information" of the publication titled "Health effects associated with exposure to intimate partner violence against women and childhood abuse: a burden of proof study". We conducted a systematic search of 7 databases (PubMed; Embase/Elsevier; Cumulative Index to Nursing and Allied Health Literature (CINAHL), PsychInfo; Global Index Medicus; Cochrane; Web of Science) to identify relevant literature published from January 1, 1970 (aligning with systematic review standards) to September 30, 2021. As of February 15, 2023, our searches were updated to incorporate articles published and/or added to databases between September 30, 2021 and January 31, 2023. Our search was then further updated on February 26, 2024 to capture literature published through January 31, 2024. The search strings applied to each database are reported in Section 4.1 and have been previously published in our review protocol. Across the seven databases searched, we found 386,331 results, of which 310,999 were duplicative, leaving 75,331 unique studies. Systematic reviews that were captured through our search were flagged for separate citation searching process (Supplementary Information Section 4.3). All sources were title/abstract and full text screened for acceptance based on the inclusion and exclusion criteria detailed in section 4.2.

Section 4.1: Literature searches

PubMed Search String

("Sex Offenses"[mh] OR "Violence"[mh:noexp] OR "Domestic Violence"[mh] OR "Gender-Based Violence"[mh] OR "Intimate Partner Violence"[mh] OR "Physical Abuse"[mh] OR "Rape"[mh] OR "Torture"[mh] OR "Workplace Violence"[mh] OR "Gun violence"[mh] OR "Battered Women"[mh] OR "Adult Survivors of Child abuse"[mh] OR "Exposure to Violence"[mh] OR "Emotional Abuse"[mh] OR "Sexual Harassment"[mh] OR "Harassment, Non-Sexual"[mh:noexp] OR "Aggression"[mh:noexp] OR "Coercion"[Mesh] OR "Dehumanization"[mh] OR "stalking"[mh] OR "adverse childhood experiences"[mh] OR violence[tiab] OR "sexual assault"[tiab] OR "sexual harassment"[tiab] OR "sexual abuse"[tiab] OR "sex abuse"[tiab] OR rape[tiab] OR "forced sex"[tiab] OR "sexual coercion"[tiab] OR "reproductive coercion"[tiab] OR "sex trafficking"[tiab] OR "sexual exploitation"[tiab] OR "forced marriage"[tiab] OR "child marriage*"[tiab] OR "early marriage*"[tiab] OR "child bride*"[tiab] OR CEFM[tiab] OR "female genital mutilation"[tiab] OR "female genital cutting"[tiab] OR "female circumcision"[tiab] OR "female genital circumcision"[tiab] OR infibulation*[tiab] OR clitoridectom*[tiab] OR clitorectom*[tiab] OR "ritual female genital surger*"[tiab] OR FGM[tiab] OR "physical abuse"[tiab] OR "psychological abuse"[tiab] OR "emotional abuse"[tiab] OR "economic abuse"[tiab] OR "financial abuse"[tiab] OR "verbal abuse"[tiab] OR maltreatment[tiab] OR "violent discipline"[tiab] OR "corporal punishment"[tiab] OR "adverse childhood experience*"[tiab] OR molestation[tiab] OR "child abuse"[tiab] OR "partner abuse"[tiab] OR "dating abuse"[tiab] OR "wife abuse"[tiab] OR "spouse abuse"[tiab] OR "domestic abuse"[tiab] OR "elder abuse"[tiab] OR "senior abuse"[tiab] OR "aged abuse"[tiab] OR victimization[tiab] OR dehumanization[tiab] OR victimisation[tiab] OR dehumanisation[tiab] OR stalking[tiab] OR cyberviolence[tiab] OR cybervictimization[tiab] OR cyberstalking[tiab])

AND
(Case-Control Studies[mh] OR Cross-Over Studies[mh] OR Cohort Studies[mh] OR Systematic Review[pt] OR Meta-Analysis[pt] OR "Twin Study"[pt] OR “systematic review”[tiab] OR “meta-analysis”[tiab] OR “cohort”[tiab] OR “cross-over”[tiab] OR “case-control”[tiab] OR “prospective”[tiab] OR “retrospective”[tiab] OR “longitudinal”[tiab] OR “follow-up”[tiab] OR “followup”[tiab])
AND
("Statistics as Topic"[mh] OR Risk[mh] OR Odds Ratio[mh] OR “risk*”[tiab] OR “odds”[tiab] OR “cross-product ratio*”[tiab] OR “hazards ratio*”[tiab] OR “hazard ratio*”[tiab] OR statistic*[tiab] OR “HR”[tiab] OR “RR”[tiab] OR “aOR”[tiab] OR relation*[tiab] OR correlat*[tiab] OR associat*[tiab] OR likel*[tiab])
AND
("1970/01/01"[PDat] : "2024/01/31"[PDat])

Embase Search String

('sexual violence'/exp OR 'forced sex'/exp OR 'violence'/de OR 'domestic violence'/exp OR 'gender based violence'/exp OR 'partner violence'/exp OR 'dating violence'/exp OR 'physical abuse'/exp OR 'physical violence'/exp OR 'torture'/exp OR 'workplace violence'/exp OR 'gun violence'/exp OR 'battered woman'/exp OR 'child abuse survivor'/exp OR 'exposure to violence'/exp OR 'emotional abuse'/exp OR 'elderly abuse'/exp OR 'sexual harassment'/exp OR 'non-sexual harassment'/de OR 'aggression'/de OR 'verbal hostility'/exp OR 'coercion'/exp OR 'intimidation'/exp OR 'dehumanization'/exp OR 'stalking'/de OR 'childhood adversity'/exp OR 'maltreatment'/exp OR 'corporal punishment'/exp OR 'victimization'/exp OR (violence OR 'sexual assault' OR 'sexual harassment' OR 'sexual abuse' OR 'sex abuse' OR rape OR 'forced sex' OR 'sexual coercion' OR 'reproductive coercion' OR 'sex trafficking' OR 'sexual exploitation' OR 'forced marriage*' OR ‘child marriage*’ OR ‘early marriage*’ OR ‘child bride*’ OR CEFM OR 'female genital mutilation' OR 'female genital cutting' OR 'female circumcision' OR 'female genital circumcision' OR infibulation* OR clitoridectom* OR clitorectom* OR ‘ritual female genital surger*’ OR FGM OR 'physical abuse' OR 'psychological abuse' OR 'emotional abuse' OR 'economic abuse' OR ‘financial abuse’ OR 'verbal abuse' OR maltreatment OR 'violent discipline' OR 'corporal punishment' OR 'adverse childhood experience*' OR molestation OR 'child abuse' OR 'partner abuse' OR 'dating abuse' OR 'wife abuse' OR 'spouse abuse' OR 'domestic abuse' OR 'elder abuse' OR 'senior abuse' OR 'aged abuse' OR victimization OR dehumanisation OR victimisation OR dehumanization OR stalking OR cyberviolence OR cybervictimization OR cyberstalking):ti,ab,kw)
AND
('case control study'/exp OR 'crossover procedure'/exp OR 'cohort analysis'/exp OR 'systematic review'/exp OR 'systematic review (topic)'/exp OR 'meta analysis'/exp OR 'meta analysis (topic)'/exp OR 'twin study'/exp OR ('systematic review' OR meta-analysis OR cohort OR cross-over OR case-control OR prospective OR retrospective OR longitudinal OR follow-up OR followup):ti,ab,kw)
AND

('statistics'/exp OR 'statistical parameters'/exp OR 'risk'/exp OR 'risk ratio'/exp OR 'correlation'/exp OR 'statistical analysis'/exp OR ('risk*' OR 'odds' OR 'cross-product ratio*' OR 'hazards ratio*' OR 'hazard ratio*' OR statistic* OR 'HR' OR 'RR' OR 'aOR' OR relation* OR correlat* OR associat* OR likel*):ti,ab,kw)
AND
[1970–2024]/py
AND
[01-01-1970]/sd NOT [31-01-2024]/sd

Cumulative Index to Nursing and Allied Health Literature (CINAHL) Search String

((MH ("Sexual Abuse+" OR "Violence" OR "Domestic Violence+" OR "Gender-Based Violence" OR "Circumcision, Female" OR "Dating Violence" OR "Torture" OR "Workplace Violence" OR "Gun Violence" OR "Battered Women" OR "Child Abuse Survivors" OR "Exposure to Violence" OR "Emotional Abuse" OR "Sexual Harassment" OR "Aggression" OR "Verbal Abuse" OR "Coercion" OR "Dehumanization" OR "Stalking" OR "Adverse Childhood Experiences" OR "Sibling Violence" OR "School Violence" OR "Student Abuse")) OR TI(violence OR "sexual assault" OR "sexual harassment" OR "sexual abuse" OR "sex abuse" OR rape OR "forced sex" OR "sexual coercion" OR "reproductive coercion" OR "sex trafficking" OR "sexual exploitation" OR "forced marriage" OR “child marriage*” OR “early marriage*” OR “child bride*” OR CEFM OR "female genital mutilation" OR "female genital cutting" OR "female circumcision" OR "female genital circumcision" OR infibulation* OR clitoridectom* OR clitorectom* OR “ritual female genital surger*” OR FGM OR "physical abuse" OR "psychological abuse" OR "emotional abuse" OR "economic abuse" OR “financial abuse” OR "verbal abuse" OR maltreatment OR "violent discipline" OR "corporal punishment" OR "adverse childhood experience*" OR molestation OR "child abuse" OR "partner abuse" OR "dating abuse" OR "wife abuse" OR "spouse abuse" OR "domestic abuse" OR "elder abuse" OR "senior abuse" OR "aged abuse" OR victimization OR dehumanization OR victimisation OR dehumanisation OR stalking OR cyberviolence OR cybervictimization OR cyberstalking) OR AB(violence OR "sexual assault" OR "sexual harassment" OR "sexual abuse" OR "sex abuse" OR rape OR "forced sex" OR "sexual coercion" OR "reproductive coercion" OR "sex trafficking" OR "sexual exploitation" OR "forced marriage" OR “child marriage*” OR “early marriage*” OR “child bride*” OR CEFM OR "female genital mutilation" OR "female genital cutting" OR "female circumcision" OR "female genital circumcision" OR infibulation* OR clitoridectom* OR clitorectom* OR “ritual female genital surger*” OR FGM OR "physical abuse" OR "psychological abuse" OR "emotional abuse" OR "economic abuse" OR “financial abuse” OR "verbal abuse" OR maltreatment OR "violent discipline" OR "corporal punishment" OR "adverse childhood experience*" OR molestation OR "child abuse" OR "partner abuse" OR "dating abuse" OR "wife abuse" OR "spouse abuse" OR "domestic abuse" OR "elder abuse" OR "senior abuse" OR "aged abuse" OR victimization OR dehumanization OR victimisation OR dehumanisation OR stalking OR cyberviolence OR cybervictimization OR cyberstalking))
AND

((MH ("Case Control Studies+" OR "Crossover Design" OR "Systematic Review" OR "Meta Analysis" OR "Prospective Studies+" OR "Retrospective Design")) OR TI("systematic review" OR meta-analysis OR cohort OR cross-over OR case-control OR prospective OR retrospective OR longitudinal OR follow-up OR followup) OR AB("systematic review" OR meta-analysis OR cohort OR cross-over OR case-control OR prospective OR retrospective OR longitudinal OR follow-up OR followup))
AND
((MH ("Statistics+" OR "Data Analysis, Statistical+")) OR TI(risk* OR odds OR "cross-product ratio*" OR "hazards ratio*" OR "hazard ratio*" OR statistic* OR "HR" OR "RR" OR "aOR" OR relation* OR correlat* OR associat* OR likel*) OR AB(risk* OR odds OR "cross-product ratio*" OR "hazards ratio*" OR "hazard ratio*" OR statistic* OR "HR" OR "RR" OR "aOR" OR relation* OR correlat* OR associat* OR likel*)))
Limits: Publication Date: January 1970 – January 2024

PsycINFO Search String

(DE("Sexual Violence" OR "Sex Offenses" OR "Sexual Abuse" OR "Sexual Coercion" OR "Sex Trafficking" OR "Violence" OR "Domestic Violence" OR "Child Abuse" OR "Elder Abuse" OR "Circumcision" OR "Intimate Partner Violence" OR "Dating Violence" OR "Physical Abuse" OR "Physical Discipline" OR "Punishment" OR "Rape" OR "Acquaintance Rape" OR "Torture" OR "Workplace Violence" OR "Gun Violence" OR "Battered Females" OR "Exposure to Violence" OR "Emotional Abuse" OR "Sexual Harassment" OR "Aggressive Behavior" OR "Verbal Abuse" OR "Coercion" OR "Stalking" OR "Childhood Adversity" OR "School Violence" OR "Police Violence" OR "Victimization")
OR
MA("Sex Offenses" OR "Domestic Violence" OR "Gender-Based Violence" OR "Intimate Partner Violence" OR "Physical Abuse" OR "Rape" OR "Torture" OR "Workplace Violence" OR "Gun violence" OR "Battered Women" OR "Adult Survivors of Child abuse" OR "Exposure to Violence" OR "Emotional Abuse" OR "Sexual Harassment" OR "Coercion" OR "Dehumanization" OR "stalking" OR “adverse childhood experiences”)
OR
TI(violence OR "sexual assault" OR "sexual harassment" OR “sexual abuse” OR “sex abuse” OR rape OR “forced sex” OR “sexual coercion” OR “reproductive coercion” OR “sex trafficking” OR “sexual exploitation” OR “forced marriage*” OR “child marriage*” OR “early marriage*” OR “child bride*” OR CEFM OR “female genital mutilation” OR “female genital cutting” OR “female circumcision” OR “female genital circumcision” OR infibulation* OR clitoridectom* OR clitorectom* OR “ritual female genital surger*” OR FGM OR “physical abuse” OR “psychological abuse” OR “emotional abuse” OR “economic abuse” OR “financial abuse” OR "verbal abuse" OR maltreatment OR “violent discipline” OR “corporal punishment” OR “adverse childhood experience*” OR molestation OR “child abuse” OR “partner abuse” OR "dating abuse" OR “wife abuse” OR “spouse abuse” OR “domestic abuse” OR “elder abuse” OR “senior abuse” OR “aged

abuse” OR victimization OR dehumanization OR victimisation OR dehumanisation OR stalking OR cyberviolence OR cybervictimization OR cyberstalking)

OR

AB(violence OR "sexual assault" OR "sexual harassment" OR “sexual abuse” OR “sex abuse” OR rape OR “forced sex” OR “sexual coercion” OR “reproductive coercion” OR “sex trafficking” OR “sexual exploitation” OR “forced marriage*” OR “child marriage*” OR “early marriage*” OR “child bride*” OR CEFM OR “female genital mutilation” OR “female genital cutting” OR “female circumcision” OR “female genital circumcision” OR infibulation* OR clitoridectom* OR clitorectom* OR “ritual female genital surger*” OR FGM OR “physical abuse” OR “psychological abuse” OR “emotional abuse” OR “economic abuse” OR “financial abuse” OR "verbal abuse" OR maltreatment OR “violent discipline” OR “corporal punishment” OR “adverse childhood experience*” OR molestation OR “child abuse” OR “partner abuse” OR "dating abuse" OR “wife abuse” OR “spouse abuse” OR “domestic abuse” OR “elder abuse” OR “senior abuse” OR “aged abuse” OR victimization OR dehumanization OR victimisation OR dehumanisation OR stalking OR cyberviolence OR cybervictimization OR cyberstalking)

OR

KW(violence OR "sexual assault" OR "sexual harassment" OR “sexual abuse” OR “sex abuse” OR rape OR “forced sex” OR “sexual coercion” OR “reproductive coercion” OR “sex trafficking” OR “sexual exploitation” OR “forced marriage*” OR “child marriage*” OR “early marriage*” OR “child bride*” OR CEFM OR “female genital mutilation” OR “female genital cutting” OR “female circumcision” OR “female genital circumcision” OR infibulation* OR clitoridectom* OR clitorectom* OR “ritual female genital surger*” OR FGM OR “physical abuse” OR “psychological abuse” OR “emotional abuse” OR “economic abuse” OR “financial abuse” OR "verbal abuse" OR maltreatment OR “violent discipline” OR “corporal punishment” OR “adverse childhood experience*” OR molestation OR “child abuse” OR “partner abuse” OR "dating abuse" OR “wife abuse” OR “spouse abuse” OR “domestic abuse” OR “elder abuse” OR “senior abuse” OR “aged abuse” OR victimization OR dehumanization OR victimisation OR dehumanisation OR stalking OR cyberviolence OR cybervictimization OR cyberstalking))

AND

(DE("Cohort Analysis" OR "Followup Studies" OR "Longitudinal Studies" OR "Retrospective Studies" OR "Prospective Studies" OR "Systematic Review" OR "Meta Analysis")

OR

MA("Case-Control Studies" OR "Cross-Over Studies" OR "Cohort Studies")

OR

TI("systematic review" OR meta-analysis OR cohort OR cross-over OR case-control OR prospective OR retrospective OR longitudinal OR follow-up OR followup)

OR

AB("systematic review" OR meta-analysis OR cohort OR cross-over OR case-control OR prospective OR retrospective OR longitudinal OR follow-up OR followup)

OR

KW("systematic review" OR meta-analysis OR cohort OR cross-over OR case-control OR prospective OR retrospective OR longitudinal OR follow-up OR followup))
AND
(DE("Statistics" OR "Statistical Analysis" OR "Risk Assessment" OR "Statistical Correlation")
OR
MA("Statistics as Topic" OR Risk OR Odds Ratio)
OR
TI(risk* OR odds OR "cross-product ratio*" OR "hazards ratio*" OR "hazard ratio*" OR statistic* OR "HR" OR "RR" OR "aOR" OR relation* OR correlat* OR associat* OR likel*)
OR
AB(risk* OR odds OR "cross-product ratio*" OR "hazards ratio*" OR "hazard ratio*" OR statistic* OR "HR" OR "RR" OR "aOR" OR relation* OR correlat* OR associat* OR likel*)
OR
KW(risk* OR odds OR "cross-product ratio*" OR "hazards ratio*" OR "hazard ratio*" OR statistic* OR "HR" OR "RR" OR "aOR" OR relation* OR correlat* OR associat* OR likel*))
Limits: Publication Date: January 1970 – January 2024

Global Index Medicus Search String

(((mh:(I01.198.240.748* OR Violence OR I01.198.240.856.350* OR I01.198.240.856.463 OR I01.198.240.856.575* OR I01.198.240.856.688 OR I01.198.240.748.640 OR I01.198.240.856.825 OR I01.198.240.856.912 OR I01.198.240.856.519 OR M01.975.155 OR M01.135.500 OR I01.880.735.900.869 OR I01.880.735.305 OR SP9.020.800.010 OR "Harassment, Non-Sexual" OR "Aggression" OR I01.880.604.316 OR Dehumanization OR F01.145.813.191* OR I01.880.735.223.500 OR I01.880.735.035)) OR (ti:(violence OR "sexual assault" OR "sexual harassment" OR "sexual abuse" OR "sex abuse" OR rape OR "forced sex" OR "sexual coercion" OR "reproductive coercion" OR "sex trafficking" OR "sexual exploitation" OR "forced marriage" OR "forced marriages" OR "child marriage" OR "child marriages" OR "early marriage" OR "early marriages" OR "child bride" OR "child brides" OR CEFM OR "female genital mutilation" OR "female genital cutting" OR "female circumcision" OR "female genital circumcision" OR infibulation* OR clitoridectom* OR clitorectom* OR "ritual female genital surgery" OR "ritual female genital surgeries" OR FGM OR "physical abuse" OR "psychological abuse" OR "emotional abuse" OR "economic abuse" OR "financial abuse" OR "verbal abuse" OR maltreatment OR "violent discipline" OR "corporal punishment" OR "adverse childhood experience" OR "adverse childhood experiences" OR molestation OR "child abuse" OR "partner abuse" OR "dating abuse" OR "wife abuse" OR "spouse abuse" OR "domestic abuse" OR "elder abuse" OR "senior abuse" OR "aged abuse" OR victimization OR dehumanization OR victimisation OR dehumanisation OR stalking)) OR (ab:(violence OR "sexual assault" OR "sexual harassment" OR "sexual abuse" OR "sex abuse" OR rape OR "forced sex" OR "sexual coercion" OR "reproductive coercion" OR "sex trafficking" OR "sexual exploitation" OR "forced marriage" OR "forced marriages" OR "child marriage" OR "child marriages" OR "early marriage" OR "early marriages" OR "child bride" OR "child brides" OR CEFM OR "female genital mutilation" OR "female genital cutting" OR "female

circumcision" OR "female genital circumcision" OR infibulation* OR clitoridectom* OR clitorectom* OR "ritual female genital surgery" OR "ritual female genital surgeries" OR FGM OR "physical abuse" OR "psychological abuse" OR "emotional abuse" OR "economic abuse" OR "financial abuse" OR "verbal abuse" OR maltreatment OR "violent discipline" OR "corporal punishment" OR "adverse childhood experience" OR "adverse childhood experiences" OR molestation OR "child abuse" OR "partner abuse" OR "dating abuse" OR "wife abuse" OR "spouse abuse" OR "domestic abuse" OR "elder abuse" OR "senior abuse" OR "aged abuse" OR victimization OR dehumanization OR victimisation OR dehumanisation OR stalking OR cyberviolence OR cybervictimization OR cyberstalking)))

AND

((mh:(E05.318.372.500.500* OR E05.318.370.150 OR E05.318.372.500.750* OR V03.850 OR L01.178.682.759.575 OR V03.600 OR E05.318.370.500* OR V03.900)) OR (ti:("systematic review" OR "meta-analysis" OR cohort OR "cross-over" OR "case-control" OR Prospective OR retrospective OR longitudinal OR "follow-up" OR followup)) OR (ab:("systematic review" OR "meta-analysis" OR cohort OR "cross-over" OR "case-control" OR Prospective OR retrospective OR longitudinal OR "follow-up" OR followup)))

AND

((mh:9E05.318.740* OR E05.318.740.600.800* OR E05.318.740.600.600)) OR (ti:(risk* OR odds OR "cross-product ratio" OR "cross-product ratios" OR "hazards ratio" OR "hazards ratios" OR "hazard ratio" OR "hazard ratios" OR statistic* OR "HR" OR "RR" OR "aOR" OR relation* OR correlat* OR associat* OR likel*) OR (ab:(risk* OR odds OR "cross-product ratio" OR "cross-product ratios" OR "hazards ratio" OR "hazards ratios" OR "hazard ratio" OR "hazard ratios" OR statistic* OR "HR" OR "RR" OR "aOR" OR relation* OR correlat* OR associat* OR likel*))))

AND

(year_cluster:[1970 TO 2024])

Cochrane Search String

([mh "Sex Offenses"] OR [mh ^"Violence"] OR [mh "Domestic Violence"] OR [mh "Gender-Based Violence"] OR [mh "Intimate Partner Violence"] OR [mh "Physical Abuse"] OR [mh "Rape"] OR [mh "Torture"] OR [mh "Workplace Violence"] OR [mh "Gun violence"] OR [mh "Battered Women"] OR [mh "Adult Survivors of Child abuse"] OR [mh "Exposure to Violence"] OR [mh "Emotional Abuse"] OR [mh "Sexual Harassment"] OR [mh ^"Harassment, Non-Sexual"] OR [mh ^"Aggression"] OR [mh "Coercion"] OR [mh "Dehumanization"] OR [mh "stalking"] OR [mh “adverse childhood experiences”] OR (violence OR "sexual assault" OR "sexual harassment" OR "sexual abuse" OR "sex abuse" OR rape OR "forced sex" OR "sexual coercion" OR "reproductive coercion" OR "sex trafficking" OR "sexual exploitation" OR ((forced OR child OR early) NEXT marriage*) OR (child NEXT bride*) OR CEFM OR "female genital mutilation" OR "female genital cutting" OR "female circumcision" OR "female genital circumcision" OR infibulation* OR clitoridectom* OR clitorectom* OR "ritual female genital surgery" OR "ritual female genital surgeries" OR FGM OR ((physical OR psychological OR emotional OR economic OR financial OR verbal) NEXT abuse) OR maltreatment OR "violent discipline" OR "corporal punishment" OR

"adverse childhood experience" OR "adverse childhood experiences" OR molestation OR "child abuse" OR "partner abuse" OR "dating abuse" OR "wife abuse" OR "spouse abuse" OR "domestic abuse" OR "elder abuse" OR "senior abuse" OR "aged abuse" OR victimization OR dehumanization OR victimisation OR dehumanisation OR stalking OR cyberviolence OR cybervictimization OR cyberstalking):ti,ab,kw)
AND
([mh "Case-Control Studies"] OR [mh "Cross-Over Studies"] OR [mh "Cohort Studies"] OR [mh "Systematic Review"] OR [mh "Meta-Analysis"] OR [mh "Twin Study"] OR ("systematic review" OR "meta-analysis" OR "cohort" OR "cross-over" OR "case-control" OR "prospective" OR "retrospective" OR "longitudinal" OR "follow-up" OR "followup"):ti,ab,kw)
AND
([mh "Statistics as Topic"] OR [mh Risk] OR [mh "Odds Ratio"] OR ("risk" OR "odds" OR "cross-product ratio" OR "cross-product ratios" OR "hazards ratio" OR "hazards ratios" OR "hazard ratio" OR "hazard ratios" OR statistic* OR "HR" OR "RR" OR "aOR" OR relation* OR correlat* OR associat* OR likel*):ti,ab,kw)
Limits: January 1970 – January 2024

Web of Science Core Collection Search String

(TS=(
(violence OR "sexual assault" OR "sexual harassment" OR "sexual abuse" OR "sex abuse" OR rape OR "forced sex" OR "sexual coercion" OR "reproductive coercion" OR "sex trafficking" OR "sexual exploitation" OR "forced marriage*" OR “forced marriage*” OR “child marriage*” OR “early marriage*” OR “child bride*” OR CEFM OR "female genital mutilation" OR "female genital cutting" OR "female circumcision" OR "female genital circumcision" OR infibulation* OR clitoridectom* OR clitorectom* OR “ritual female genital surger*” OR FGM OR "physical abuse" OR "psychological abuse" OR "emotional abuse" OR "economic abuse" OR “financial abuse” OR "verbal abuse" OR Maltreatment OR torture OR "violent discipline" OR "corporal punishment" OR "adverse childhood experience*" OR molestation OR "child abuse" OR "partner abuse" OR "dating abuse" OR "wife abuse" OR "battered wom*n" OR "spouse abuse" OR "domestic abuse" OR "elder abuse" OR "senior abuse" OR "aged abuse" OR victimization OR dehumanization OR victimisation OR dehumanisation OR stalking OR cyberviolence OR cybervictimization OR cyberstalking)
AND
("systematic review" OR "meta-analysis" OR cohort OR cross-over OR case-control OR prospective OR retrospective OR longitudinal OR follow-up OR followup)
AND
("risk*" OR "odds" OR "cross-product ratio*" OR "hazards ratio*" OR "hazard ratio*" OR statistic* OR "HR" OR "RR" OR "aOR" OR relation* OR correlat* OR associat* OR likel*)))
AND
DOP=(1970-01-01/2024-01-31)

Section 4.2: Inclusion and exclusion criteria

Each review step (title/abstract screening, full-text screening, and data extraction) began with consensus building exercises across the review team. After training and consensus-building, the first two-thirds of titles/abstracts were reviewed by two independent reviewers, with conflicts resolved by project leaders. Upon confirmation of a low rate of total conflicts (<5% of total screened), the remainder of titles/abstracts were single screened. Non-English articles were screened by reviewers with proficiency in the language. Studies which met inclusion criteria during title/abstract screening (n = 4620) were full text screened and excluded if found to meet any exclusion criteria. Two independent reviewers full text screened 10% of articles, with conflicts resolved by project leads. Upon confirming a low conflict rate (<5%), the remaining 90% of articles were single screened.

Table S12. Inclusion and exclusion criteria applied during screening

Inclusion Criteria	Exclusion Criteria
**Study design:** case-control, cohort, or case-crossover studies.<br><br>**Participants:** Studies conducted in participant groups likely to be generalizable to the population of interest. Exposed groups are defined as any individual who has experienced a form of sexual, physical, and/or psychological GBV throughout the lifetime. Comparators will be non-exposed control groups, or study groups without reported exposure to a form of sexual, physical, and/or psychological GBV.<br><br>**Outcomes:** Studies reporting an estimate of association (either RR, risk ratio, odds ratio, hazard ratio or similar) or reporting cases and non-cases among those exposed and unexposed. If not provided directly, studies providing enough information to allow an estimate of RR to be calculated will meet inclusion criteria.	**Study design**: Cross-sectional, ecological, case series or case studies.<br><br>**Participants**: Studies conducted in subgroups identified only by convenience sampling or subgroups identified via a shared characteristic that is likely related to risk of exposure to violence or the reported health outcome (e.g., domestic violence shelter residents).<br><br>**Exposure measurement**: Studies that report only an aggregate measure of exposure combining exposure to a form of violence with other, non-eligible exposures (e.g., reports a composite score only) will be excluded. For these studies, we are unable to disentangle the effect of violence exposure from the effects of other hardships or exposure types, preventing their inclusion in our review.<br><br>**Does not meet minimum reporting criteria**: Studies missing essential data, that is, those that do not report effect sizes and uncertainty information (confidence intervals, sample sizes) or the data needed to impute an effect size with uncertainty information.

Table S13. Inclusion and exclusion criteria applied during data cleaning

After the screening and extraction of articles identified, we introduced another layer of inclusion and exclusion criteria applied to data cleaning that pertain to our definitions of included risk factors. Studies that met this set of criteria during data cleaning were applied into the models for final results.

Inclusion Criteria	Exclusion Criteria
**Exposure period:** Observations that do not focus exclusively on violence against children (exposure under 18 years old).<br><br>**Studies with multiple forms of observations:** Violence exposure observation definitions that precisely match to the exact definitions of individual risk factors of gender-based sexual violence, physical GBV, or psychological GBV (i.e. non-combination forms of exposure).<br><br>**Outcome definitions:** Exact outcome definitions to GBD outcome of interest and/or deviations to outcome definition without including other outcomes.	**Exposure period:** Studies that focus solely on violence against children and data points relating to exposure during childhood, when reported separately.<br><br>**Studies with multiple forms of observations:** Violence exposure observation definitions that combine more than one form of violence exposure (i.e. exposure to sexual and/or physical violence).<br><br>**Outcome definitions:** Aggregate outcome definitions that are non-GBD outcomes and/or include other health conditions.

Section 4.3: Systematic review and meta-analysis citation searching

We title and abstract and full text screened systematic reviews and/or meta-analyses yielded by our searches according to the criteria outlined for all other articles. We then grouped accepted systematic reviews/meta-analyses by unique risk-outcome pair combination in order to extract the citations identified by these reviews. If more than one systematic review was identified for a given risk-outcome pair, one systematic review was selected for citation searching on the basis of publication recency, number of included underlying studies and study quality. Study quality was determined via adherence to PRISMA and GATHER guidelines and by the impact factor of the journal the study was published within.

Once a single systematic review was selected for citation searching per unique risk-outcome pair, we extracted the references identified within each review and de-duplicated them against our primary search records. All new articles from this search were then screened for inclusion with the same criteria as in our larger review (see supplementary section 4.2).

Across all risk outcome pairs, we accepted over 200 articles from the citations of other meta-analyses in title/abstract screening and 57 articles in full-text screening. Thus, this citation-searching step of our review yielded 57 new studies for extraction.

Section 4.4: Data Extraction

All studies were extracted using a modified data extraction template in Covidence data extraction 2.0.

Table S14. Data extraction template

Name		Definition
Study locations	Location Name	The country or IHME sub-national location where the study took place
	Location ID	The location ID corresponding to the country or IHME sub-national location of the study
Specific Location		Required if the study took place in a location smaller than the corresponding location name and ID
Study Name		Required if cohort is named, do not use the article title. This field is used to screen out duplicative data, so please ensure spelling is correct.
Year Start		Year the study started
Year End		Year the study ended (NOT publication year)
Study Design	Prospective cohort	Must select one
	Retrospective cohort	
	Case-control	
	Case-cohort	
	Case-crossover	
Pooled cohort		Cohort studies only: Yes if the reported effect size is from a pooled analysis and only pooled effect size has been reported, otherwise no
Study Selection Criteria		Please specify the selection criteria of the study that is used in the analysis
Location Representative		Specify if the participants were representative of the study's geography or not
Cohort Study: Drop-out rate		Study dropout rate (%) at the end of the study entered as a decimal
Cohort Study: Drop-out rate assessment		Specify how dropout rate was defined in the study.
Cohort Study: Follow-up measure		Cohort studies only: Type of follow up measure reported (eg, 'average participant follow-up was 126 days' then select 'mean').
Cohort Study: Follow-up units		Cohort studies only: enter units of follow-up duration reported (eg, 'average participant follow-up was 126 days' then enter 'days').

**Cohort Study: Follow-up value**	Cohort studies only: Enter the length of participant follow-up if reported (eg, 'average participant follow-up was 126 days' then enter 126).
**Case-control Study: Percent of participants for which data ascertained**	Percent of participants (%) from total, for which the study has included data entered as a decimal
**Case-control Study: Controls selected from community**	Were the controls selected from the community? Yes or No
**Exposure assessment method**	Self-report, routinely collected/ administrative data, clinical examination
**Exposure assessment instrument**	Specify the name of the exposure assessment instrument. For self-reported exposures, please specify the name of the questionnaire. If more than one instrument, specify all. If the instrument is not names/designed specifically for the study, write "study-specific instrument"
**Exposure assessment period**	How many times information on exposure to type of violence
**Exposure assessment value**	If "exposure assessment period" is multiple, specify the number of times that exposure was assessed (excluding baseline)
**Outcome assessment method**	Select how the study ascertained which participants experienced the outcome
**Outcome assessment instrument**	Specify the name of the outcome assessment instrument. For self-reported outcomes, please specify the name of the questionnaire. If more than one instrument, specify all. If the instrument is not names/designed specifically for the study, write "study-specific instrument"
**Effect size measure**	Select the form of effect size used in the study
**Uncertainty type**	
**Confidence interval level**	If uncertainty is reported as a confidence interval, this column represents the confidence level which is reported at (Eg. 95, 90, 99).
**Extractor notes**	Please use this field to include any notes about the study or your extraction not covered elsewhere.
**Outcome name**	Outcome that is measured in this model
**Outcome type**	Please specify if the outcome definition included incidence of or mortality from a disease endpoint.
**Outcome definition**	Please specify the definition for the outcome as reported in the study.
**Exposure definition**	Please specify the definition for the exposed participants exactly as reported in the study.
**Exposed level**	Enter level of exposure defined for exposed group. If study's exposure is binary (any vs. none), select 'any exposure' for exposed. For all other exposure levels, select other and enter level as reported (eg, 'exposed 3–5 times').

**Unexposed definition**		Provide a brief description of the unexposed group (i.e., the comparison group) as used in estimation of the relative risk
**Unexposed level**		Enter level of exposure defined for unexposed (comparator group). If the study's exposure is binary (any vs. none), select 'no exposure' for unexposed. For all other exposure levels, select other and enter level as reported (eg, 'exposed 1–2 times').
**Violence type**		Select all violence types included for this model
**Violence type combination**		Select HOW the violence types are combined in the model.<br>(1) AND: the model must specify that the exposure group experienced BOTH/ALL types of violence selected in the previous question<br>(2) AND/OR: the exposure group consists of people who experience EITHER type of violence<br>(3) ONLY: model specifies that the exposure group experienced selected violence type, but not other types of violence included in the study.<br><br>*NOTE: Use 'only' option when it has been confirmed that is the ONLY type of violence a participant experienced (eg, experienced sexual but NOT physical violence). Otherwise, if one type of violence assessed and exposure to others are unknown, use 'unknown'.*
**Perpetrator type**		Select all perpetrator types included in this model
**Temporality of Exposure**	**Lower (Age)**	Lower bound of age range provided of when participants experienced violence. If no lower age is provided, fill in this box with '0' and provide more information in the "other information" question for this model (e.g. "temporality of exposure defined as "Childhood and adolescence")
	**Upper (Age)**	Upper bound of age range provided of when participants experienced violence. If no upper age is provided, fill in this box with "99" and provide more information in the "other information" question for this model (e.g. "temporality of exposure defined as "Childhood and adolescence")
**Exposure Recall Type**		Specify whether the exposure occurred throughout lifetime, past year, or indicate other recall type.
**Percent Female**		For the sample of this model, what percent are female (0–1) on a per 1 basis (eg, 43% female should be recorded as 0.43). Enter 1 if sample is only female. Enter 0 if sample is only male. If sample includes both sexes and percent female not reported, enter 99.
**Ages**	**Lower**	Lower bound of age of participants included in this model at time of study. If the model includes participants of all ages from the study, this box will match the lower age box in section 2.
	**Upper**	Upper bound of age of participants included in this model at time of study. If the model includes participants of all ages from the study, this box will match the upper age box in section 2.
	**Mean**	Mean of age of participants included in this model.

	**SD**	SD of age of participants included in this model.
**Subgroup analysis**		**PER UNIQUE RISK-OUTCOME PAIR IN A STUDY:** Yes if this effect size is a sub-analysis reported IN ADDITION TO a main analysis from all participants (e.g., study reports effect size for combined sexes and also effect sizes separately for males and females). If study only reports effect sizes from specific subgroups (e.g., reports effect sizes from males and females separately, without reporting a combined effect size), select no.
**Subgroup analysis free text**		If a sub-analysis, describe stratifier (i.e. age, sex, etc.)
**Effect size**	**Mean**	Mean effect size of model
	**Lower (UI)**	Lower bound of effect size
	**Upper (UI)**	Upper bound of effect size
	**Other uncertainty value**	
**Effect size table #**		Table number where you found effect size from literature
**Sample size**	**Number of cases**	Enter number of participants with measured outcome for each group reported: exposed, unexposed, total
	**Number of participants**	Enter number of participants included in analysis for each group reported: exposed, unexposed, total
	**Person-time (cohort studies)**	Enter person-time for each group reported: exposed, unexposed, total
**Person-time units (cohort studies)**		If cohort study and person-time entered into sample size table, specify the units of person-time reported
**Confounders**		Select all confounders for the most adjusted model, write in any confounders (separated by comma) not included in pre-specified list
**Other information**		Any additional information

Section 5: Differences between Spencer et al. (2023) and current study

This article is an updated version of the systematic review, so it includes more recent data. This builds off the 2023 study to quantify and evaluate the health effects of GBV by taking a broader lens on GBV exposure as a health risk among men and women. While we continue to include all forms of IPV against women in this research, we also extended our analysis of GBV exposure to include physical, sexual, and psychological GBV against women by non-partner perpetrators,

and include data that characterized men's exposure to physical and psychological IPV and sexual violence. The 2023 estimates of the health risks associated with physical and/or sexual IPV against women, specifically, have been reported separately only including data up to January 31, 2023, while the current estimates present an expanded view of health risks for physical GBV, sexual violence, and psychological GBV, distinctly, against any individual, perpetrated by partner or former partner and unspecified or unrestricted perpetrators with one additional year of data. Ultimately, these two papers complement each other to provide a comprehensive view of the health effects of gender-based violence across its various dimensions.

Table S15. Differences between Spencer et al. (2023) and current study

	**Spencer et al. (2023)**	**Current study**
Search dates	January 1, 1970, through January 31, 2023	January 1, 1970, through January 31, 2024
Types of exposure	Focused solely on IPV	Physical, psychological, or sexual GBV
Exposure definitions	The paper examines the health effects of physical and/or sexual intimate partner violence against women as a composite exposure category.<br>Psychological IPV was not explicitly included in the reported meta-analysis. The only way psychological IPV was considered was as a component of accepted alternate case definitions that also included physical and/or sexual IPV. No data points only focused on psychological IPV were included in that meta-analysis nor was psychological IPV discussed in the paper.	Explored the distinct health consequences of physical, psychological, and sexual GBV (including, but not limited to, IPV) as separate exposure categories.<br><br>The present manuscript does include a handful of data points that focus solely on psychological IPV in our psychological violence models, but it does not include combined exposure definitions that span multiple forms of GBV (or IPV) unlike many of those included in Spencer et al. (2023).<br><br>Our analyses include some of the same data points where a study reported either an effect size for physical IPV or an effect size for sexual IPV, but it does not include composite exposure categories.
Participants	Only women	Our analysis parses out the distinct health effects of physical, sexual, and psychological GBV without distinguishing the gender of the survivors.

		Men included, so the selection of relative risks is different compared to the IPV paper
Perpetrator	Physical and/or sexual violence perpetrated by an intimate partner	Physical GBV, sexual violence, and psychological GBV perpetrated by partner or former partner and unspecified or unrestricted perpetrators
Data point selection	When some studies may have reported effect sizes for composite physical and/or sexual IPV as well as exposure-stratified effect sizes, Spencer et al. (2023) analysis have selected the composite one.<br><br>Spencer et al. (2023) would have only used the women-specific effect size.	While the current study has selected the exposure-specific ones.<br><br>Similarly, some studies may have reported an effect size for violence among men and women as well as sex-stratified ones. We would have selected the combined effect size.
Number of included studies	IPV (n = 57 studies)	Physical Violence (n = 26)<br>Sexual Violence (n = 25)<br>Psychological Violence (n = 14)
Health outcomes	5 health outcomes<br>Major depressive disorder<br>Maternal abortion and miscarriage<br>HIV/AIDS<br>Anxiety disorders<br>Self-harm	8 health outcomes<br>Major depressive disorder<br>Maternal abortion and miscarriage<br>HIV/AIDS<br>Anxiety disorders<br>Self-harm<br>Sexually transmitted infections excluding HIV<br>Drug use disorders<br>Alcohol use disorders

Figure S22. Comparison between mean relative risk and strength of the evidence for the association of multiple forms of GBV and IPV and 8 health outcomes

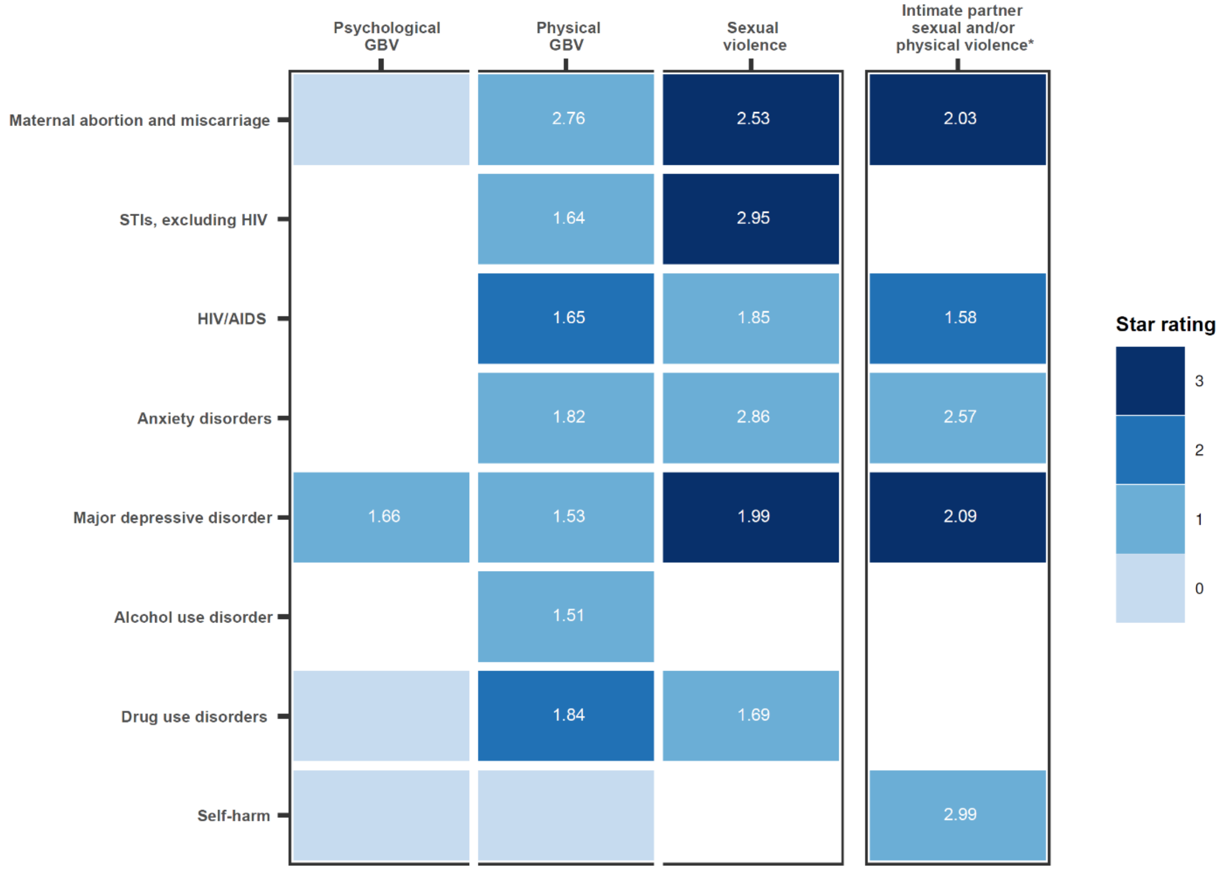

*Only women included

Section 6: Included Exposure and Outcome Definitions

Section 6.1: Exposure and outcome definitions

The definitions listed in Table S16 of sexual violence, physical GBV, and psychological GBV were adapted using the International Classification of Violence against Children (ICVAC), which broke down forms of violence against children into categories, providing a useful framework for thinking and operationalizing different kinds of GBV exposures as well.

Table S16. Definitions of included risk factors

Risk factor name	Definition
**Sexual Violence**	Any deliberate, unwanted, and non-essential sexual act, either completed or attempted, that is perpetrated against an individual, including for exploitative purposes, and that results in or has a high likelihood of resulting in injury, pain, or psychological suffering. It

	includes, but is not limited to, rape, sexual assault, and non-contact sexual acts.
**Physical GBV**	Any deliberate, unwanted, and non-essential act that uses physical force against the body of an individual due to gender-related reasons and that results in or has a high likelihood of resulting in injury, pain, or psychological suffering. It includes, but is not limited to, severe and minor assault.
**Psychological GBV**	Any deliberate, unwanted, and non-essential act, verbal and non-verbal, due to gender-related reasons that has long-term physiological harm and mental health consequences on an individual. It includes, but is not limited to, terrorizing, harassing, spurning, humiliating, and controlling an individual.

Table S17. Summarized exposure definitions for GBV used in included studies

**Physical GBV definitions**
Experienced physical abuse (undefined)
Experienced physical violence during pregnancy, including having a weapon used against them, being slapped, punched, shoved, kicked, bruised, or burned
Experienced having a weapon used against them, being stabbed, shot at, punched, kicked, slapped, whipped, or burned by a partner
Experienced physical abuse from a partner, including being hit, kicked, or slapped
Experienced physical abuse from a spouse
Experienced physical abuse from a partner that resulted in injury, receiving medical treatment, or external involvement
Experienced physical abuse including being hit, thrown, pushed, or shaken

**Psychological GBV definitions**
Experienced psychological violence (unspecified)
Experienced fear
Experienced fear of partner
Partner verbally abused, insulted, or humiliated them
Experienced psychological violence from partner
Experienced highly controlling behavior from partner
Experienced verbal abuse
Experienced harassment, including stalking or phone-based harassment

**Sexual violence definitions**
Experienced sexual abuse (undefined)
Experienced sexual intimate partner violence
Experienced sexual assault or attempted sexual assault
Experienced rape
Experienced marital rape

Experienced rape or sexual assault
Experienced sexual coercion
Experienced rape by a non-partner
Experienced rape or attempted rape

The outcomes examined in this review were defined in accordance with the cause definitions used in the GBD study. We followed cause-specific research team guidance to accept GBD reference and alternate case definitions. For accepted alternate case definitions, we incorporated study-level bias covariates to detect if the use of an alternate definitions significantly biased final model results.

Table S18. Definitions of included outcomes

Cause Grouping	Cause Name	Definition
**Mental health disorders**	Major depressive disorder	Major depressive disorder assessed according to DSM-4 (296.21–24, 296.31–34) and ICD-10 criteria (F32.0–9, F33.0–9). Diagnostic interviews and symptom scales were accepted according to the GBD criteria (Supplemental Table 4).
	Anxiety disorders	Anxiety disorders involving experiences of intense fear and distress in combination with other physiological symptoms.
**Substance use disorders**	Alcohol use disorder	A maladaptive pattern of substance use, leading to clinically significant impairment or distress, as manifested by three (or more) of the DSM-4 criteria for substance dependence occurring any time in a 12-month period.
	Drug use disorders	This aggregate cause incorporates death and disability resulting from opioid use disorder, amphetamine use disorder, cocaine use disorder, cannabis use disorder, and a residual category of other drug use disorders including deaths and disability due to dependence on hallucinogens, inhalants, solvents, and sedatives.
**Maternal disorders**	Maternal abortion and miscarriage	Abortion is defined as elective or medically indicated termination of pregnancy at any gestational age and miscarriage is defined as spontaneous loss of pregnancy before 24 weeks of gestation with complications requiring medical care.
**HIV/AIDS and Sexually Transmitted Infections**	HIV/AIDS	HIV/AIDS as assessed by biomarker tests.
	Sexually transmitted infections excluding HIV	Sexually transmitted infections are viral, bacterial, or parasitic infections that are transmitted through sexual contact. This aggregate group includes syphilis, chlamydia, gonorrhea, genital herpes, trichomoniasis, and a residual category of other sexually transmitted

		infections including chancroid, granuloma inguinale, and unspecified sexually transmitted diseases.
**Injuries**	Self-harm	Self-harm is deliberate bodily damage inflicted on oneself resulting in death or injury (ICD-9: E950–E959, ICD-10: X60–X64.9, X66–X84.9, Y87.0).

Section 6.1.1: Additional description of accepted definitions and measurement tools for depressive and anxiety disorders

For depressive and anxiety disorders specifically, we received an inventory of acceptable diagnostic interview and symptom scales from the mental health research team at IHME. These tools have been reviewed in terms of their validity and specificity to measuring the symptoms of these specific disorders. We included studies using accepted diagnostic interviews (reference) or symptom scales (accepted alternate). A list of acceptable tools is included in Table S19. For other mental disorders, we followed GBD case definitions and accepted studies measuring outcomes by use of International Disease Classification and Diagnostic and Statistical Manual of Mental Disorder criteria.

Table S19. Depressive and anxiety disorder accepted diagnostic interview and symptom scale measurement tools

Measurement tool type	List of accepted tools
**Diagnostic interviews (reference method of measurement)**	- Structured Clinical Interview for DSM-IV Axis Disorders (SCID-I) (1 month, lifetime)<br>- Structured Clinical Interview for DSM-IV-TR Research Version, Non-patient edition (SCID-I/NP).<br>- Schedules for Clinical Assessment in Neuropsychiatry (SCAN)<br>- Mini International Neuropsychiatric Interview (MINI) (2 weeks, 2Y dysthymia)<br>- Mini International neuro-psychiatric Interview for children and Adolescents (MINI-KID)<br>- Diagnostic Interview Schedule (DIS) or Diagnostic Interview Schedule-IV (DIS-IV)<br>- Chinese modified Diagnostic Interview Schedule (DIS-CM)<br>- Composite International Diagnostic Interview (CIDI) (1 year, lifetime)<br>- University of Michigan Composite International Diagnostic Interview (UM-CIDI)<br>- Korean version of the Composite International Diagnostic Interview (K-CIDI)<br>- Munich-Composite International Diagnostic Interview (M-CIDI)<br>- Geriatric Mental State Schedule (GMS) (1 month) - AGECAT (Automated Geriatric Examination for Computer Assisted Taxonomy)<br>- Primary Care Evaluation of Mental Disorders (PRIME-MD)<br>- Development and Well-Being Assessment (DAWBA)<br>- Schedule for Affective Disorders and Schizophrenia (SADS)<br>- Kiddie Schedule for Affective Disorders and Schizophrenia (K-SADS-PL)<br>- Comprehensive Psychopathological Rating Scale (CPRS)

	- The Alcohol Use Disorder and Associated Disabilities Interview Schedule-IV (AUDADIS-IV) (1 year, lifetime)<br>- Diagnostic Interview for Children and Adolescents (DICA)<br>- SPIKE interview<br>- Clinical Interview Schedule-Revised (CIS-R) (1 week)<br>- Diagnostic Interview Schedule for Children Version IV (DISC)<br>- Diagnostic interview schedule for children-young child version (DISC-YC)<br>- Diagnostic Interview Schedule for Children, Parent Report (DISC-P)<br>- Present state examination (PSE)<br>- Child and adolescent psychiatric assessment (CAPA)<br>- Preschool age psychiatric assessment (PAPA)<br>- Children's Depression Rating Scale–Revised (CDRS-R)
**Symptom scales (alternate method of measurement)**	- Patient Health Questionnaire (PHQ)<br>- Revised Brief Patient Health Questionnaire (Brief PHQ-R)<br>- Beck Depression Inventory (BDI)<br>- Center for Epidemiologic Studies Depression Scale (CES-D)<br>- Center for Epidemiologic Studies Depression Scale for Children (CES-DC)<br>- Hospital Anxiety and Depression Scale (HADS)<br>- Depression Anxiety Stress Scale (DASS)<br>- Reynolds Adolescent Depression Scale (RADS)<br>- Child Depression Inventory (CDI)<br>- Duke Anxiety-Depression scale (DUKE-AD)<br>- Emotional State Questionnaire (EST-Q)<br>- Hopkins Symptom Checklist (HSCL) -DMI (25 items)<br>- Health & Daily Living Form (HDL)<br>- Child behavior checklist (CBCL)<br>- Hamilton depression rating scale (HAM-D)<br>- Harvard Department of Psychiatry National Depression Screening Day Scale (HANDS)<br>- Children’s Depression Scale (CDS)<br>- Major Depression Inventory (MDI)<br>- Quick Inventory of Depressive Symptomology (QIDS)

Section 6.1.2: Additional description of accepted definitions for substance use disorders

For all the substance use disorders modeled in the GBD, the base reference case definitions are the DSM-4 criteria for substance dependence. Dependence is defined as a maladaptive pattern of substance use, leading to clinically significant impairment or distress, as manifested by three (or more) of the following, occurring any time in a 12-month period:

- Tolerance, as defined by either of the following: (a) a need for markedly increased amounts of the substance to achieve intoxication or desired effect, or (b) markedly diminished effect with continued use of the same amount of the substance.

- Withdrawal, as manifested by either of the following: (a) the characteristic withdrawal syndrome for the substance, or (b) the same (or closely related) substance is taken to relieve or avoid withdrawal symptoms.
- The substance is often taken in larger amounts or over a longer period than intended.
- There is a persistent desire or unsuccessful efforts to cut down or control substance use.
- A great deal of time is spent in activities necessary to obtain the substance, use the substance, or recover from its effects.
- Important social, occupational, or recreational activities are given up or reduced because of substance use.
- The substance use is continued despite knowledge of having a persistent physical or psychological problem that is likely to have been caused or exacerbated by the substance (e.g., current cocaine use despite recognition of cocaine-induced depression, continued drinking despite recognition that an ulcer was made worse by alcohol consumption)

DSM-4 also provides the diagnostic criteria for substance abuse. The criteria for substance abuse focus on social and situational consequences of use and does not mention withdrawal or tolerance. For drug use disorders and alcohol use disorders, we mapped the outcome as either dependence (uses clinical diagnoses), abuse (more than frequent use but not limited to clinical case definitions), or binge drinking (heavy episodic drinking for alcohol use), or use. For cannabis and alcohol, dependence met the GBD reference case definition for these outcomes, while abuse is an acceptable alternative definition. For alcohol use, binge drinking or use fall more closely with the alcohol use risk factor, which is outside the scope of this analysis. For other drugs, dependence is the GBD reference case definition, but all other case definitions are acceptable alternatives.

To summarize, we accepted studies that consider alcohol dependence (as the gold standard outcome definition) or alcohol abuse (as an accepted alternate definition) for the alcohol use disorder model. Similarly, we accepted studies that consider drug dependence (as the gold standard outcome definition) or drug abuse (as an accepted alternate definition) for the drug use disorder models.

Section 7: Study Quality and Bias Assessment

Section 7.1: Definition of bias covariates

Following GRADE criteria, the risk of bias criteria for individual studies included in our analyses captured representativeness of the study population, exposure and outcome measurement quality, control for confounding, selection bias, and risk of reverse causation. Because our analyses covered three distinct risk factors and many different health outcomes, we created a core set of bias covariates across all risk-outcomes pairs (Table S20) as well as additional bias covariates specific to effect size adjustment, exposure definition, and outcome definitions (Table S21; Table S22; Table S23). For all covariates, the reference value is zero while indication

of the specific bias type was coded as a one. All covariates meeting eligibility requirements (i.e., at least two studies represented for each value of the covariate) were tested for significance using the selection algorithm in the MR-BRT tool. Minimum availability of two observations for each value of the covariate meant that a reduced set of covariates were able to be tested for risk-outcome pairs with low total study counts.

Table S20. Standard bias covariates created across all input datasets

Bias covariate name	Definition	Operationalized definition
Representativeness	Study sample is not geographically representative of underlying location	0 = is representative;<br>1 = not representative
Risk of selection bias	Study is at risk for selection bias with loss to follow-up (cohorts) or percent without ascertained data (case-controls) > 20%	0 = no selection bias;<br>1 = selection bias risk
Risk of reverse causation	Study is at risk of reverse causation, as in the case with case-control studies	0 = no risk of reverse causation;<br>1 = risk present
Odds ratio	Study reports an odds ratio	0 = study reports relative risk<br>1 = study reports odds ratio

Table S21. Adjustment bias covariates created across all input datasets

Bias covariate name	Definition	Operationalized definition
Unadjusted effect size	Study reports only a complete unadjusted effect size	0 = controls for any confounding;<br>1 = raw/crude effect size
Unadjusted for sex	Effect size is controlled for sex or is sex-specific	0 = accounts for sex;<br>1 = does not account for sex
Unadjusted for age	Effect size is controlled for age	0 = accounts for age;<br>1 = does not account for age
Unadjusted for age, sex, and at least one other confounding variable	Effect size controls for age, sex, and at least one other confounder	0 = controls for age, sex, and at least one other confounder;<br>1 = does not control for age or sex or at least one other confounder
Males included in the effect size	Does the effect size include males in its analytical sample?	0 = does not include males;<br>1 = includes males
Females only included in the effect size	Does the effect size only include females in its analytical sample?	0 = does not include females;<br>1 = includes females only
Effect size for both sexes combined	Does the effect size use an analytical sample that includes both sexes?	0 = not sex-specific effect size;<br>1 = sex-specific effect size

Table S22. Exposure definition bias covariates

Bias covariate name	Definition	Operationalized definition
Exposure perpetrator is specific	Is the perpetrator group limited to specifically partners or specifically non-partners?	0 = broader perpetrator group;

		1 = restricted perpetrator group
Exposure recall is not lifetime	Is the exposed definition lifetime exposure to the violence type or a shorter time period?	0 = recall type is not time-limited;<br>1 = recall type is time-limited
Violence exposure is defined as a component of reference definition	[Psychological violence]: Does the exposure definition focus on a sub-type of psychological violence based on the ICVAC framework?	0 = defined in alignment with psychological violence;<br>1 = defined as fear or controlling behavior
Exposure during pregnancy	Is the exposed group specifically exposed during pregnancy or does the study use a different recall period (lifetime or other)?	0 = recall type is beyond pregnancy;<br>1 = recall type is pregnancy
Exposure is ascertained from administrative source	Captures if exposure was ascertained from administrative databases (health systems, agency records, etc.)	0 = self-reported data;<br>1 = used administrative source for exposure ascertainment

Table S23. Outcome definition bias covariates

**Bias covariate name**	**Outcome**	**Definition**	**Operationalized definition**
Outcome defined including other disorders	Major depressive disorder	What outcome definition does the study use?	0 = definition does not include these other disorders;<br>1 = definition includes bipolar, anxiety, and other mood disorders
	HIV/AIDS and STIs		0 = definition is not only HIV;<br>1 = definition is only HIV
Outcome is defined as a specific disorder	Diabetes Mellitus Type 2		0 = outcome is not a defined type of diabetes;<br>1 = outcome is explicitly type 2 diabetes
	Substance use disorder; Drug use disorder; Alcohol use disorder; Drug use disorder sub-types		0 = outcome is dependence<br>1 = outcome is not limited to dependence
Outcome is defined as PTSD	Anxiety disorders		0 = outcome is not specifically PTSD<br>1 = outcome is PTSD
Outcome is defined as induced abortion	Maternal abortion and/or miscarriage		0 = outcome is miscarriage or unspecified abortion<br>1 = outcome is induced abortion

Section 7.2: Bias covariates for each risk-outcome pair

We report the bias covariates marked and tested for each risk-outcome pair. The value 1 indicates that the bias covariate applies to the study for that risk-outcome pair; 0 indicates that the bias covariate does not apply to the study for that risk-outcome pair; NA indicates that the bias covariate was not tested for that study in the risk-outcome pair. Bias covariates that are NA were not tested because either there were not enough differences in the bias covariates between the included studies to be testable or because they were the same as a different included bias covariate.

Table S24. Bias covariates for physical GBV

Study	Covariates						
Physical GBV & Drug use disorders							
	Representative	Risk of selection bias	Unadjusted	Effect size includes men	Uncontrolled for age, gender, and at least one other confounder	Women-only study	Restricted period of exposure recall
Martino 2005	1	1	1	0	1	1	1
Gilbert 2012	1	1	0	0	0	1	0
Nowotny 2013	0	0	0	0	0	1	0
Ehrensaft 2006	0	0	0	0	1	0	1
Ehrensaft 2006	0	0	0	1	1	0	1
Danielson 2009	0	1	1	0	1	1	0
Ahmadabadi 2019	1	1	0	1	0	0	0
Ahmadabadi 2019	1	1	0	0	0	0	0
Physical GBV & Maternal abortion and miscarriage							
	Risk of reverse causation	Unadjusted	Unadjusted for age	Partner/ former partner perpetrated violence	Induced abortion outcome	Violence experienced during pregnancy	
Leung 2002	1	1	1	1	1	0	
Ibrahim 2015	0	1	1	1	0	1	
Hailu 2023	1	0	0	1	0	1	
Abdollahi 2015	0	0	0	1	1	1	
Catak 2016	1	0	1	1	0	0	
Johri 2011	1	0	0	1	0	0	
Bourassa 2007	1	1	1	0	1	0	
Nelson 2003	1	0	0	0	0	1	
Physical GBV & Major depressive disorder							

	Risk of selection bias	Effect size includes men	Restricted period of exposure recall
Hedtke 2008	1	0	0
Ehrensaft 2006	0	0	1
Ehrensaft 2006	0	1	1
Han 2019	0	0	1
Han 2019	0	1	1
Ahmadabadi 2020	1	0	0
Ahmadabadi 2020	1	1	0
Physical GBV & Anxiety disorders			
	Effect size includes men	PTSD outcome	
Hedtke 2008	0	1	
Ehrensaft 2006	0	0	
Ehrensaft 2006	1	0	
Ehrensaft 2006	1	1	
Ehrensaft 2006	0	1	
Ahmadabadi 2020	1	0	
Ahmadabadi 2020	0	0	
Physical GBV & HIV/AIDS			
	Risk of selection bias	Unadjusted for age	
Fonck 2005	0	1	
Deyessa 2018	0	0	
Maman 2002	1	0	
Kouyoumdjian 2013	1	1	
Physical GBV & Alcohol use disorder			
	Effect size includes men		
Ehrensaft 2006	0		
Ehrensaft 2006	1		
Ahmadabadi 2019	1		
Ahmadabadi 2019	0		

Table S25. Bias covariates for sexual GBV

Study	Covariates						
Sexual violence & HIV/AIDS							
	Risk of reverse causation	Risk of selection bias	Unadjusted	Unadjusted for age	Partner/ former partner	Uncontrolled for age, gender, and	Odds ratio

					perpetrated violence	at least one other confounder	
Maman 2002	0	1	0	0	1	0	1
Quigley 2000	1	1	0	1	0	1	1
Wyatt 2002	1	0	1	1	0	1	1
Jewkes 2010	0	0	0	0	1	0	0
Abrahams 2021	0	1	0	0	0	0	0
Larsen 2016	0	0	1	1	0	1	0
Kouyoumdjian 2013	0	1	0	1	1	1	0
Birdthistle 2013	1	1	0	0	0	1	1
Burgueño 2017	1	0	0	0	0	0	1
Deyessa 2018	1	0	0	0	1	0	1
Deyessa 2018	1	0	0	0	1	0	1

Sexual violence & Major depressive disorder

	Unadjusted for age	Effect size includes men	Aggregate outcome definition	Women-only study
Ali 2009	0	0	1	1
Hedtke 2008	0	0	0	1
Benjet 2020	1	1	0	0
Austin 2020	1	0	1	0
Austin 2020	0	1	1	0

Sexual violence & Maternal abortion and miscarriage

	Unadjusted	Partner/former partner perpetrated violence	Induced abortion outcome
Leung 2002	1	1	1
Hailu 2023	0	1	0
Johri 2011	0	1	0
Bourassa 2007	1	0	1
Larsen 2016	1	0	1
Larsen 2016	1	0	0

Sexual violence & Drug use disorders

	Effect size includes men	Uncontrolled for age, gender, and at least one other confounder	Women-only study
Gilbert 2012	0	0	1
Nowotny 2013	0	0	1
Danielson 2009	0	1	0
Danielson 2009	1	1	0
Austin 2020	0	1	0
Austin 2020	1	0	0

Sexual violence & STIs, excluding HIV

	Unadjusted for age	Partner/former partner perpetrated violence
Chowdhary 2008	0	1
Allsworth 2009	0	0

Weiss 2008	1	1
Larsen 2016	1	0

Table S26. Bias covariates for psychological GBV

Study	Covariates			
Psychological GBV & Maternal abortion and miscarriage				
	Unadjusted	Unadjusted for age	Restricted period of exposure recall	Alternate outcome definition
Leung 2002	1	1	0	1
Hailu 2023	0	0	1	0
Johri 2011	0	0	1	0
Bourassa 2007	1	1	0	1
Nelson 2003	0	0	0	1
Romito 2009	0	1	1	0
Romito 2009	0	1	1	0
Psychological GBV & Drug use disorders				
	Effect size includes men			
Gilbert 2012	0			
Exner-Cortens 2013	0			
Exner-Cortens 2013	1			
Ahmadabadi 2019	0			
Ahmadabadi 2019	1			
Ahmadabadi 2019	0			

Section 8: Primary analysis funnel plots for GBV exposures and outcomes

Section 8.1: Primary analysis funnel plots for sexual violence and outcomes

Figure S23. Primary analysis funnel plot for sexual violence and maternal abortion and miscarriage

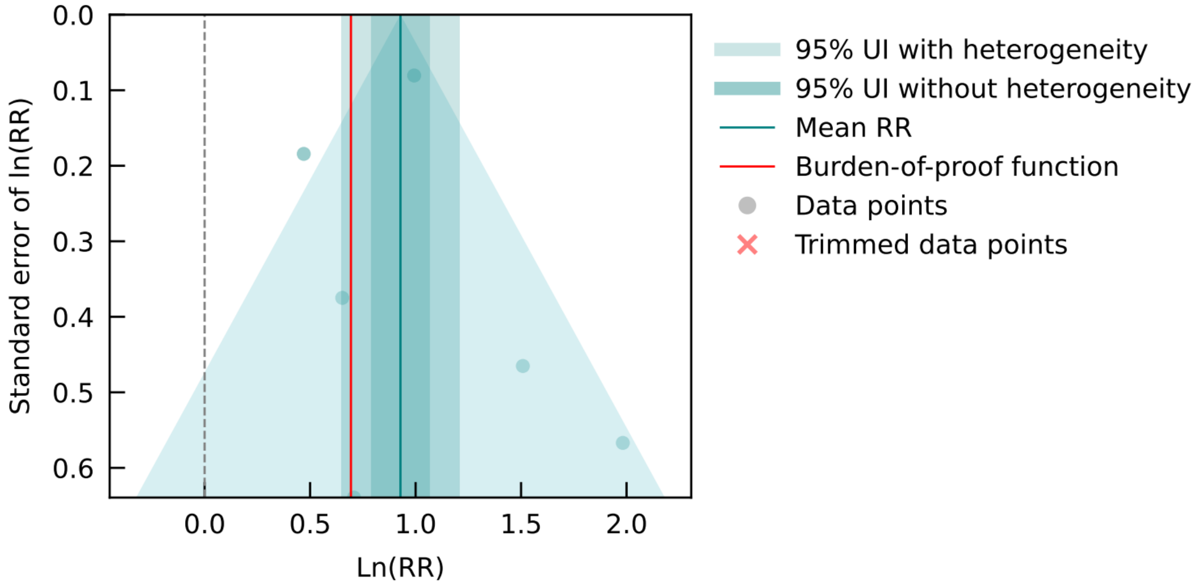

Figure S24. Primary analysis funnel plot for sexual violence and anxiety disorders

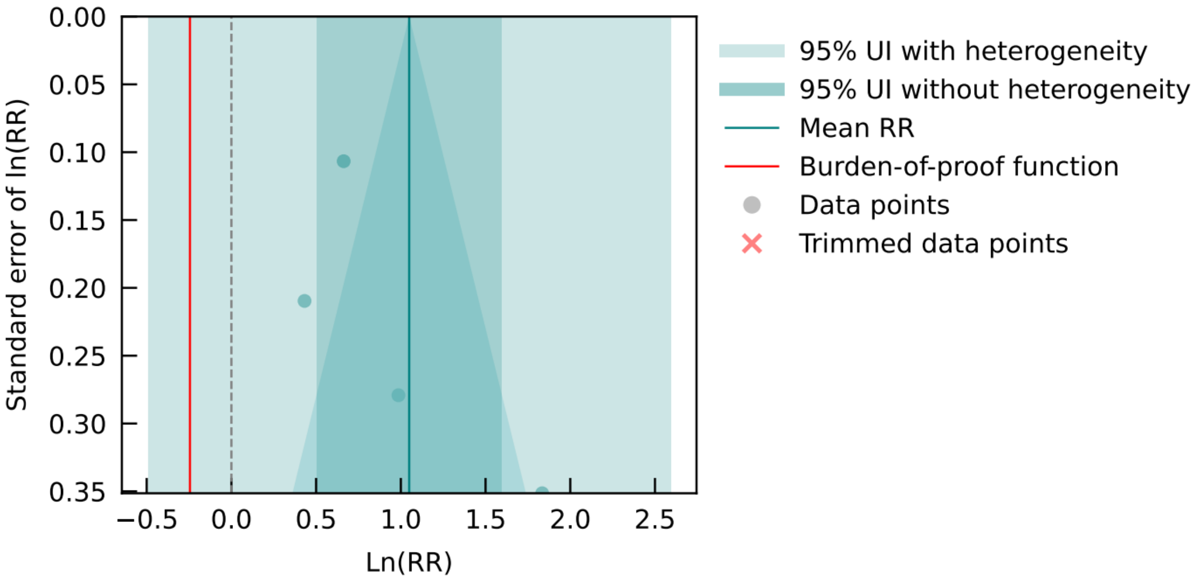

Figure S25. Primary analysis funnel plot for sexual violence and major depressive disorder

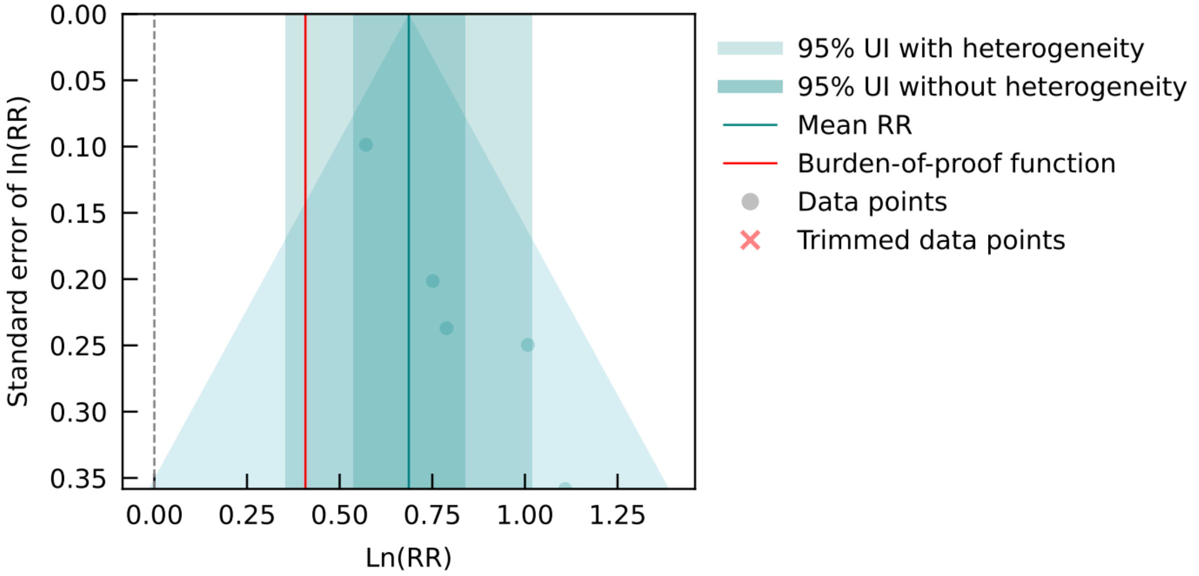

Figure S26. Primary analysis funnel plot for sexual violence and drug use disorders

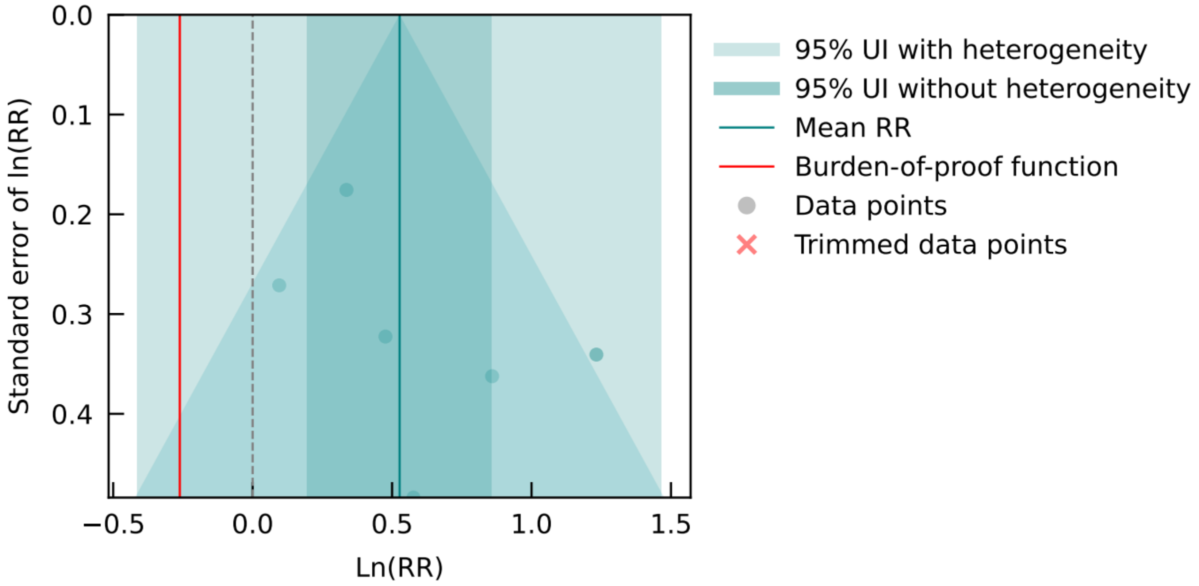

Figure S27. Primary analysis funnel plot for sexual violence and HIV/AIDS

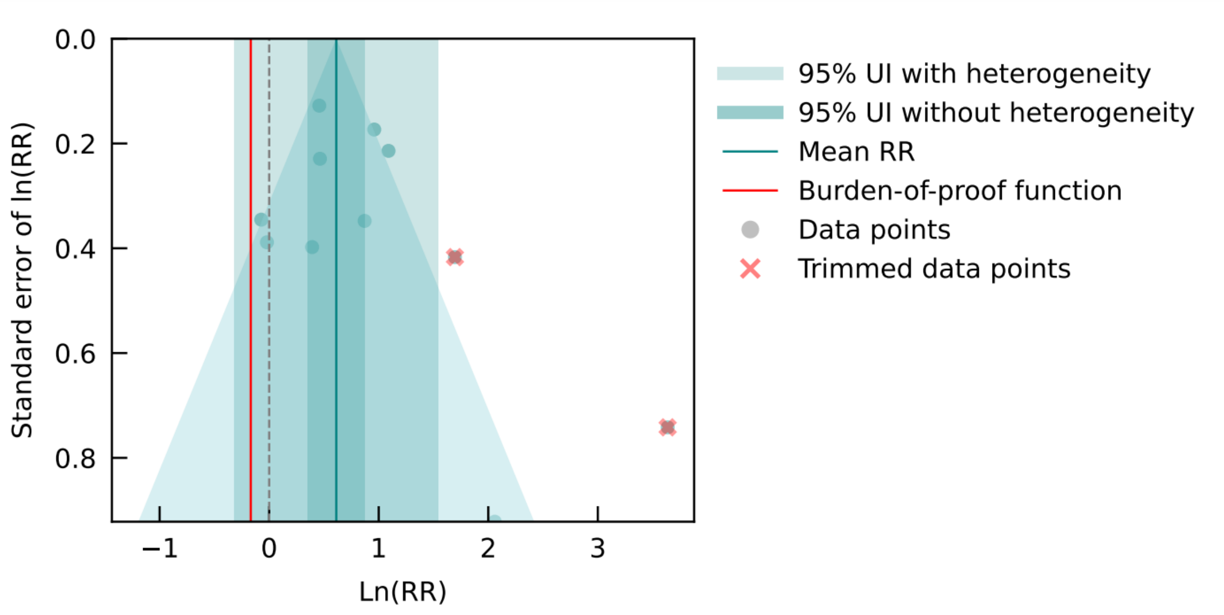

Figure S28. Primary analysis funnel plot for sexual violence and sexually transmitted infections (excluding HIV)

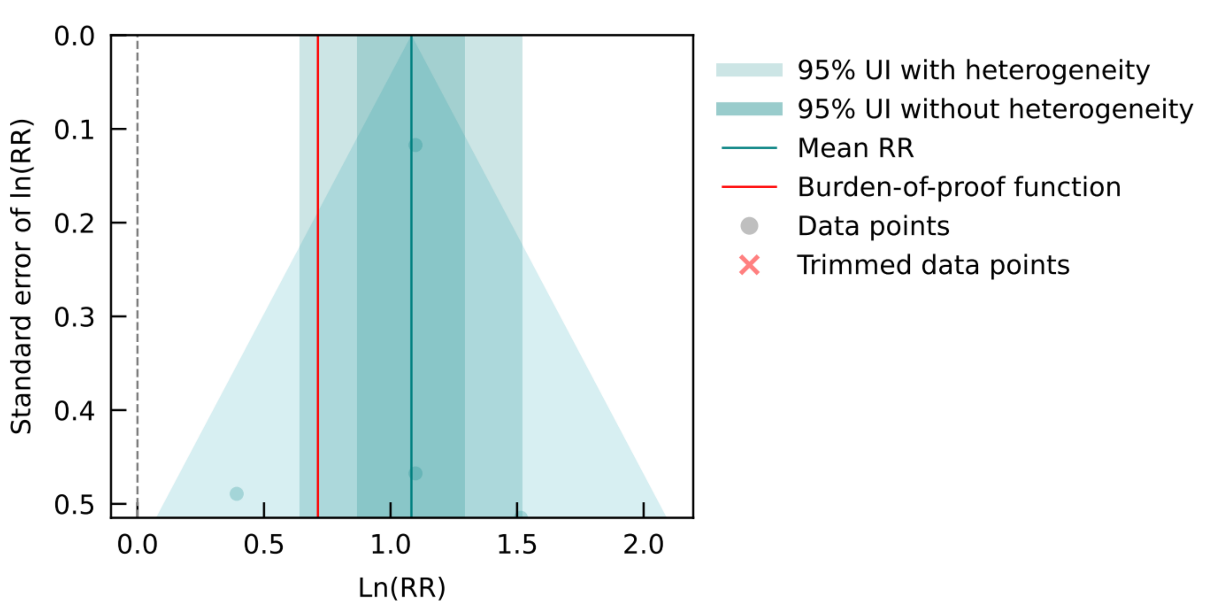

Section 8.2: Primary analysis funnel plots for physical GBV and outcomes

Figure S29. Primary analysis funnel plot for physical GBV and maternal abortion and miscarriage

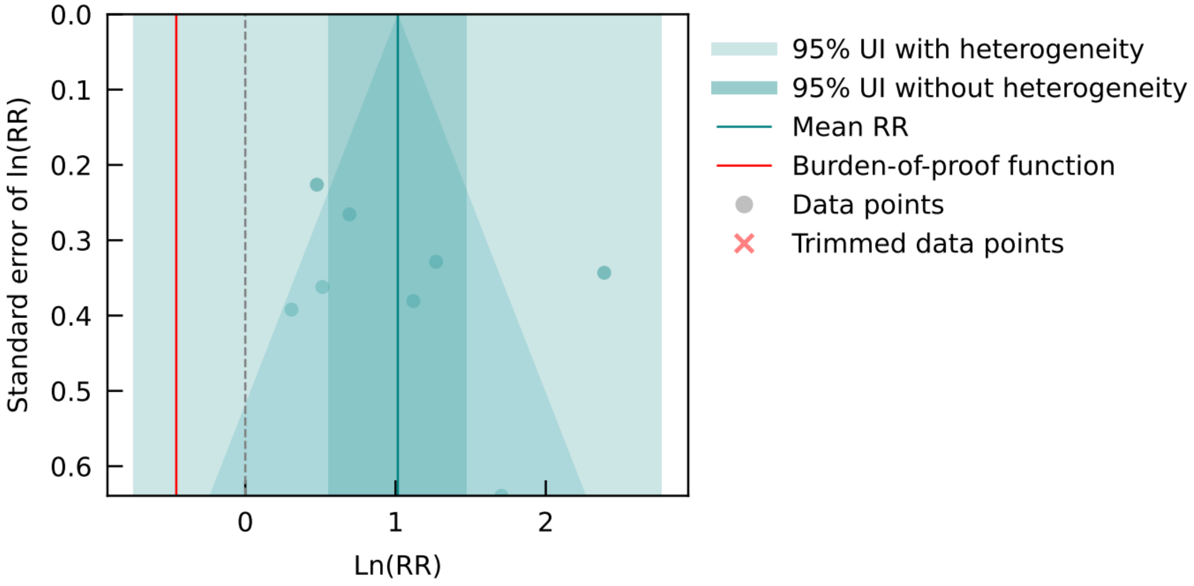

Figure S30. Primary analysis funnel plot for physical GBV and alcohol use disorders

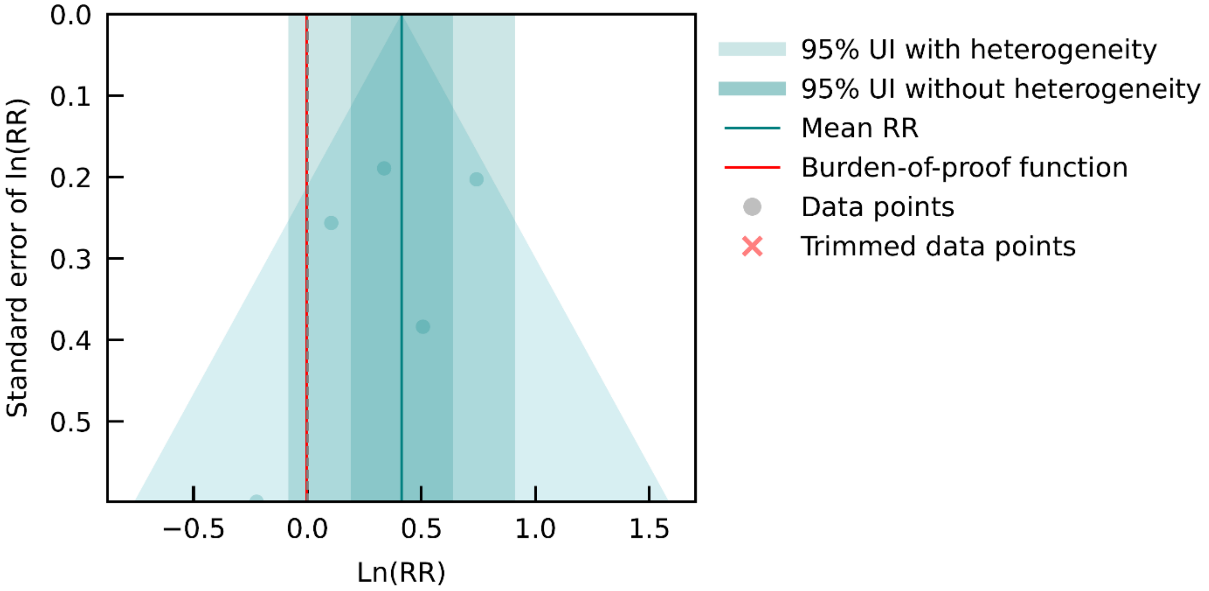

Figure S31. Primary analysis funnel plot for physical GBV and anxiety disorders

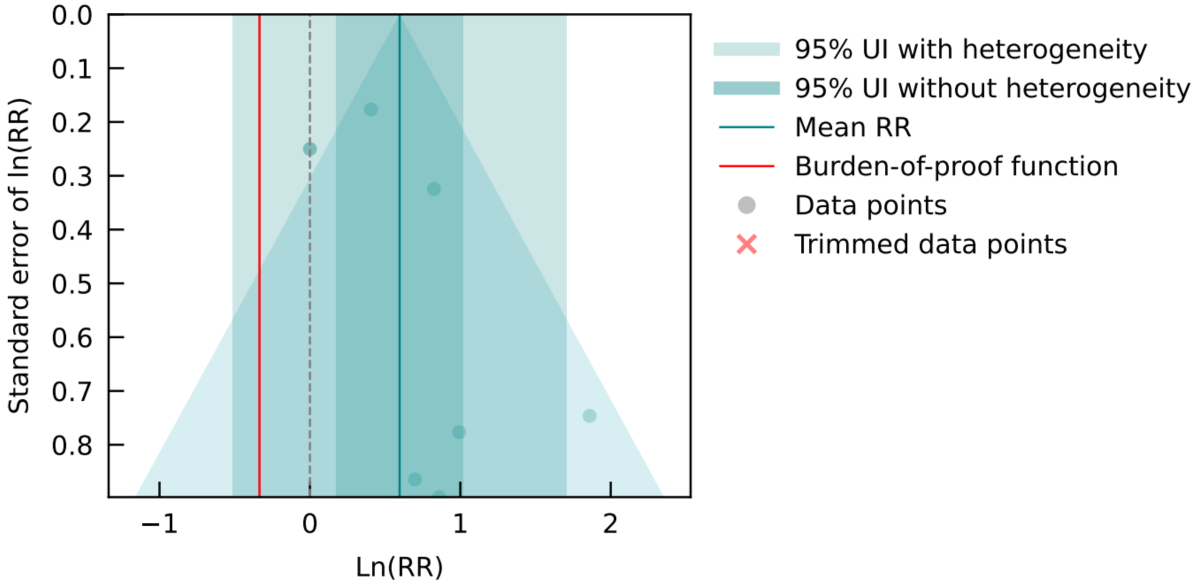

Figure S32. Primary analysis funnel plot for physical GBV and major depressive disorders

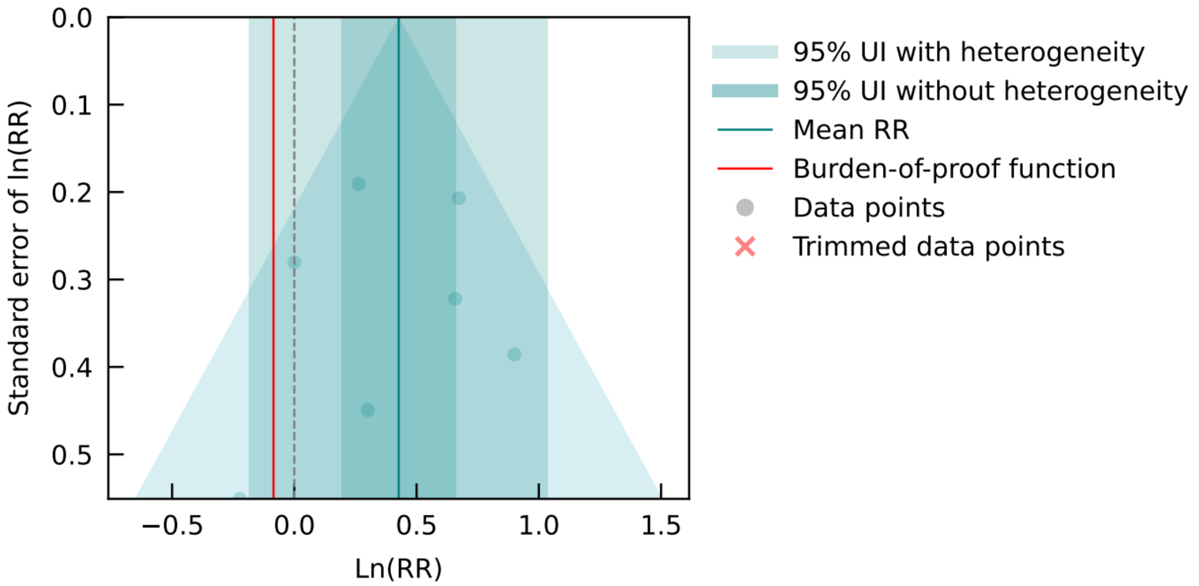

Figure S33. Primary analysis funnel plot for physical GBV and drug use disorders

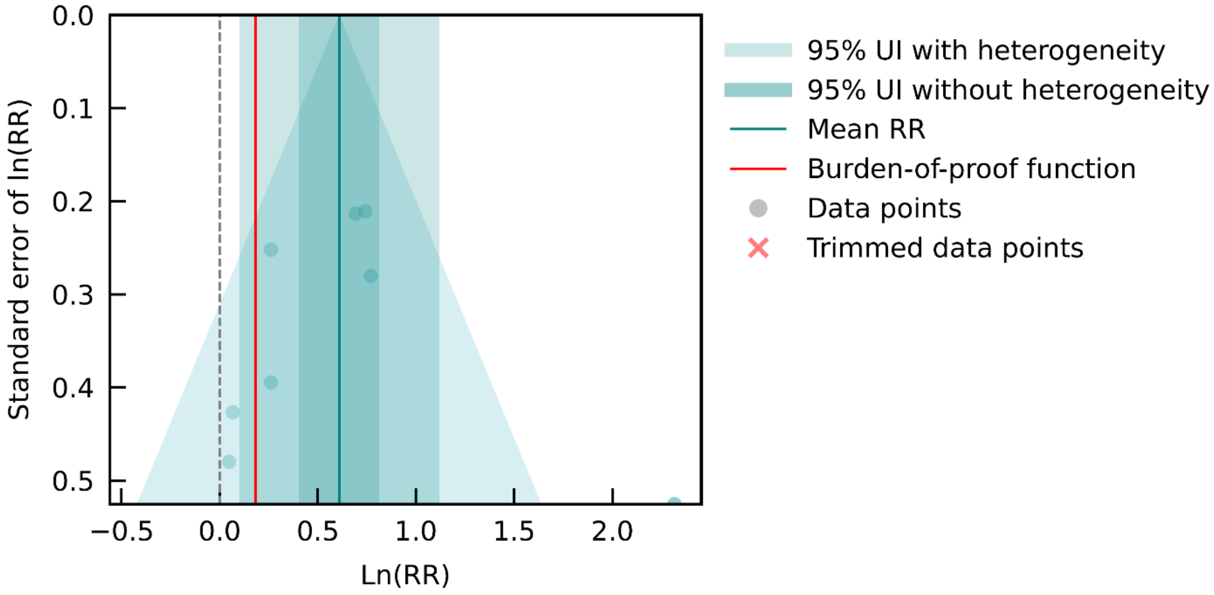

Figure S34. Primary analysis funnel plot for physical GBV and HIV/AIDS

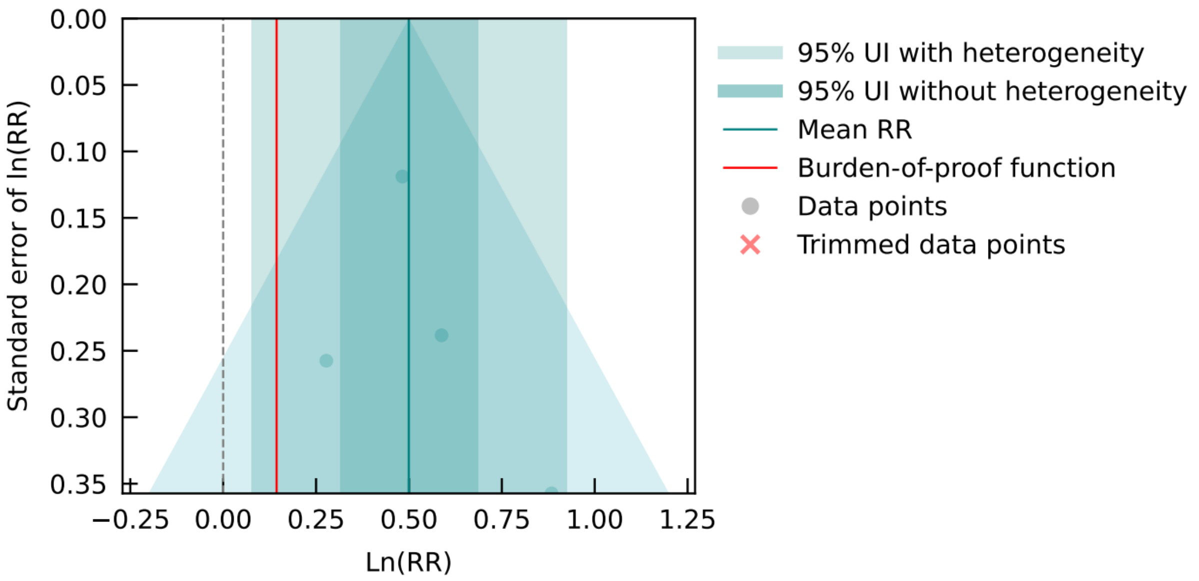

Figure S35. Primary analysis funnel plot for physical GBV and self-harm

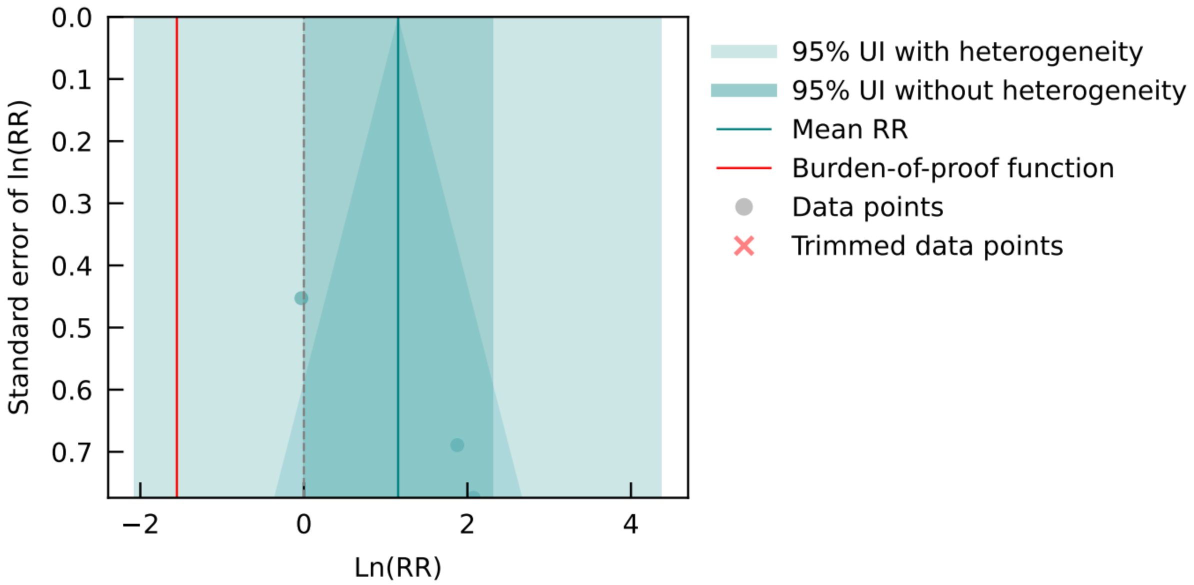

Figure S36. Primary analysis funnel plot for physical GBV and sexually transmitted infections (excluding HIV)

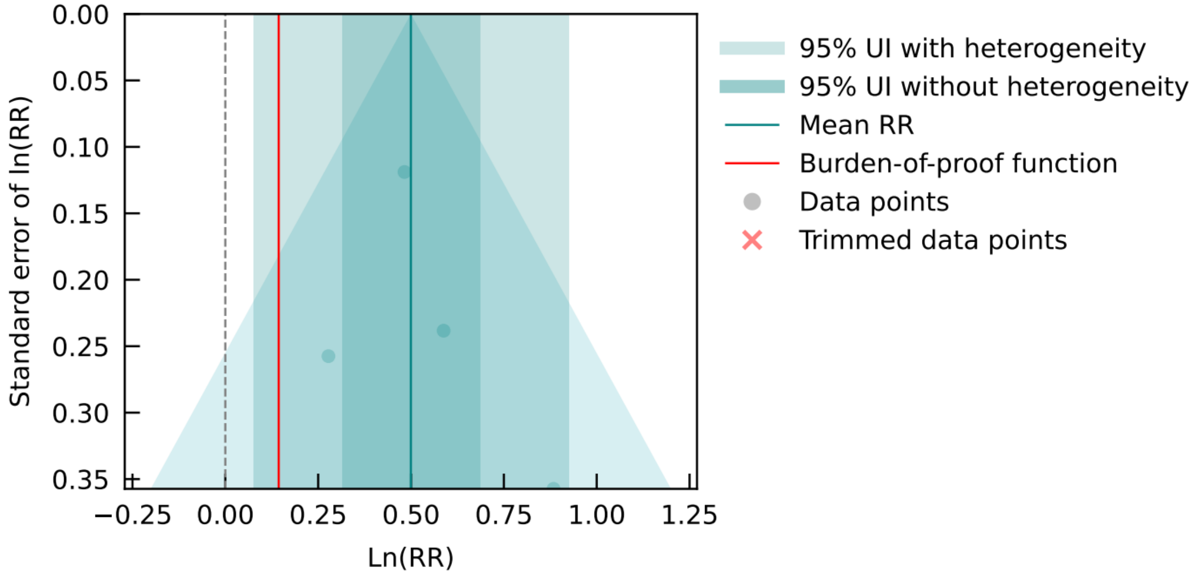

Section 8.3: Primary analysis funnel plots for psychological GBV and outcomes

Figure S37. Primary analysis funnel plot for psychological GBV and maternal abortion and miscarriage

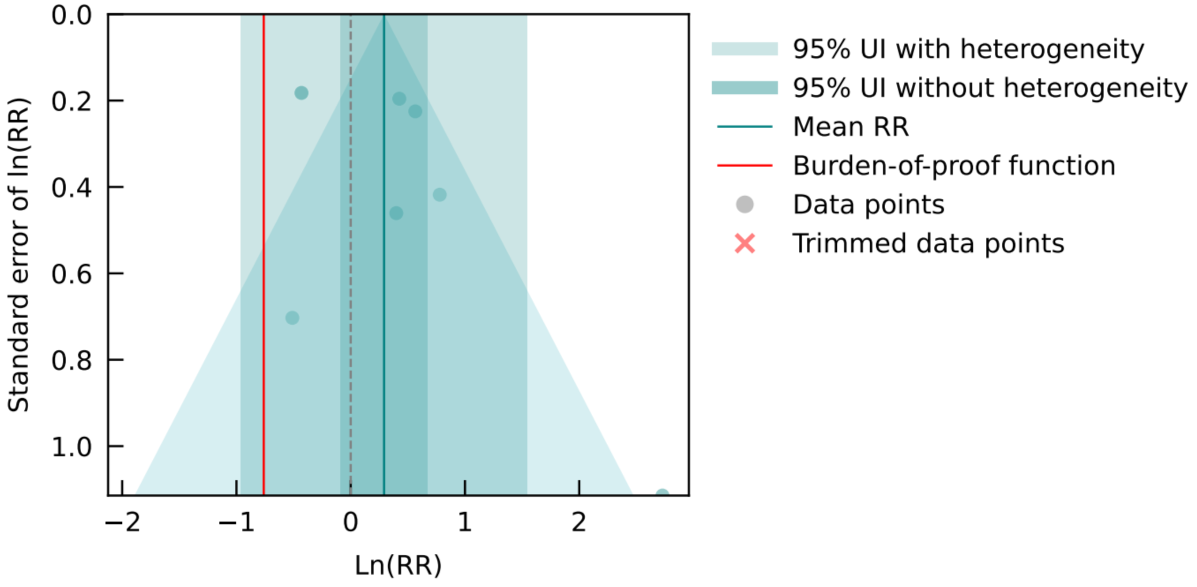

Figure S38. Primary analysis funnel plot for psychological GBV and major depressive disorder

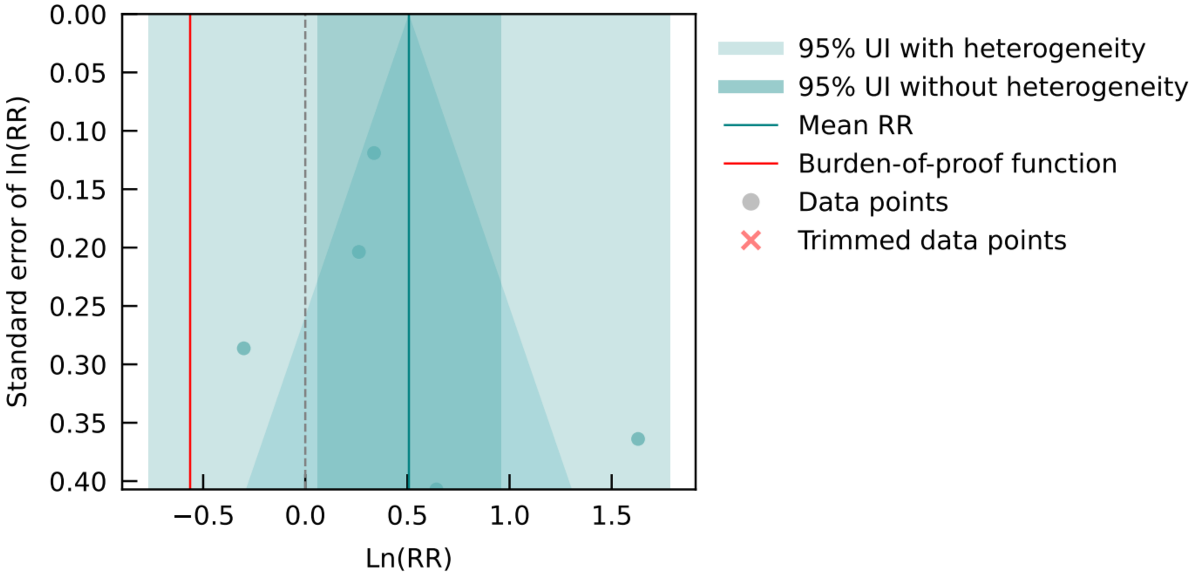

Figure S39. Primary analysis funnel plot for psychological GBV and drug use disorders

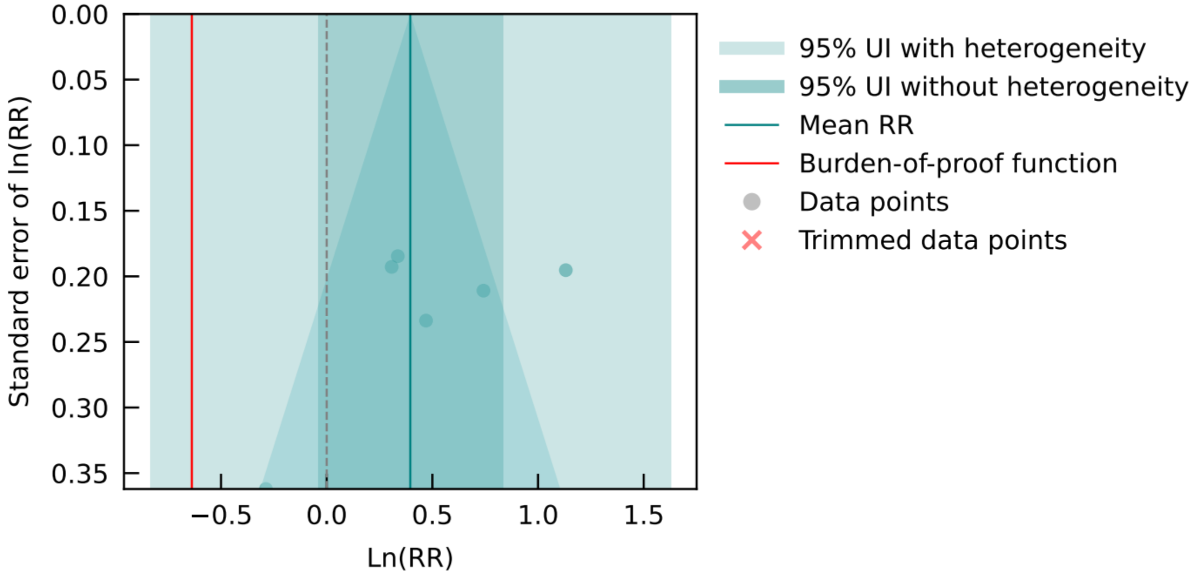

Figure S40. Primary analysis funnel plot for psychological GBV and self-harm

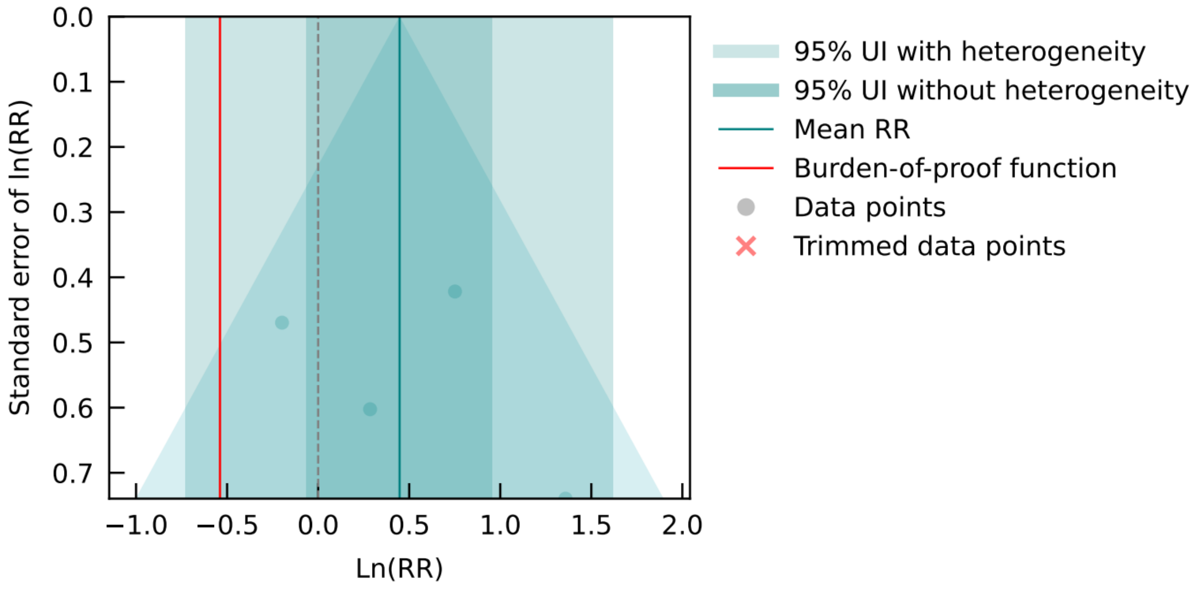

Section 9: Model Characteristics

Each risk factor and health outcome pair models were run when there were three or more unique studies that met our inclusion criteria. We applied 10% trimming for models that

included more than ten observations. For all models, exposure types were dichotomous and there were no pre-selected covariates.

Table S27. Model characteristics of GBV and corresponding health outcomes

Exposure	Outcome	Exposure Type	Pre-selected covariates	Trimming Percentage
Sexual Violence	STIs, excluding HIV/AIDS	Dichotomous	None	None
	Maternal abortion and miscarriage	Dichotomous	None	None
	Major depressive disorder	Dichotomous	None	None
	HIV/AIDS	Dichotomous	None	10% trimming
	Drug use disorders	Dichotomous	None	None
	Anxiety disorders	Dichotomous	None	None
Physical GBV	Drug use disorders	Dichotomous	None	None
	HIV/AIDS	Dichotomous	None	None
	Alcohol use disorders	Dichotomous	None	None
	Major depressive disorder	Dichotomous	None	None
	STIs, excluding HIV/AIDS	Dichotomous	None	None
	Anxiety disorders	Dichotomous	None	None
	Maternal abortion and miscarriage	Dichotomous	None	None
	Self-harm	Dichotomous	None	None
Psychological GBV	Self-harm	Dichotomous	None	None
	Maternal abortion and miscarriage	Dichotomous	None	None
	Drug use disorder	Dichotomous	None	None
	Major depressive disorder	Dichotomous	None	None

Section 10: Data for the Primary Analyses

In this section, we provide the data inputs used in the primary analyses following data cleaning and adjusting for overlapping observations.

Table S28. Data inputs included in the primary analyses

Study and Year	ln(RR)	Standard error of ln(RR)
Physical GBV & Maternal abortion and miscarriage		
Leung 2002	1.704748	0.639264

Ibrahim 2015	2.388763	0.343185
Hailu 2023	1.118415	0.380824
Abdollahi 2015	0.307485	0.392102
Catak 2016	0.693147	0.265677
Johri 2011	0.476234	0.226207
Bourassa 2007	1.269761	0.328581
Nelson 2003	0.512824	0.362061
Physical GBV & Alcohol use disorders		
Ehrensaft 2006	-0.22314	0.598379
Ehrensaft 2006	0.506818	0.383693
Danielson 2009	0.104360	0.256325
Ahmadabadi 2019	0.336472	0.18927
Ahmadabadi 2019	0.741937	0.202788
Physical GBV & Anxiety disorders		
Hedtke 2008	0.824175	0.324652
Ehrensaft 2006	0.993252	0.776653
Ehrensaft 2006	0.698135	0.864548
Ehrensaft 2006	0.858662	0.89736
Ehrensaft 2006	1.859418	0.74622
Ahmadabadi 2020	0	0.250212
Ahmadabadi 2020	0.405465	0.176823
Physical GBV & Major depressive disorder		
Hedtke 2008	0.65752	0.322047
Ehrensaft 2006	0.900161	0.385641
Ehrensaft 2006	0.300105	0.449374
Han 2019	0.672944	0.207086
Han 2019	-0.22314	0.550889
Ahmadabadi 2020	0.262364	0.190616
Ahmadabadi 2020	0	0.280258
Physical GBV & Drug use disorders		
Martino 2005	0.770108	0.280258
Gilbert 2012	0.067659	0.426688
Nowotny 2013	0.262364	0.252197
Ehrensaft 2006	2.316488	0.525399
Ehrensaft 2006	0.04879	0.479882
Danielson 2009	0.262364	0.394786
Ahmadabadi 2019	0.693147	0.213329
Ahmadabadi 2019	0.741937	0.210887
Physical GBV & HIV/AIDS		
Fonck 2005	0.587787	0.238344
Deyessa 2018	0.277632	0.257495
Maman 2002	0.883768	0.35734
Kouyoumdjian 2013	0.482426	0.1189
Physical GBV & Self-harm		
Kaslow 2000	-0.03046	0.452794
Bella 2012	1.876407	0.689315

Chowdhary 2008	2.075684	0.774012
Physical GBV & STIs excluding HIV		
Allsworth 2009	0.57098	0.274413
Weiss 2008	0.336472	0.371247
Chowdhary 2008	0.524729	0.463868
Psychological GBV & Maternal abortion and miscarriage		
Leung 2002	0.779325	0.417905
Hailu 2023	0.398776	0.4606
Johri 2011	0.425268	0.195728
Bourassa 2007	0.565314	0.225021
Nelson 2003	-0.43078	0.18231
Romito 2009	2.728506	1.114392
Romito 2009	-0.51083	0.703006
Psychological GBV & Major depressive disorder		
Fitzpatrick 2023	0.641854	0.407115
Fitzpatrick 2023	1.629241	0.363894
Han 2019	0.336472	0.119036
Han 2019	-0.30111	0.286261
Ahmadabadi 2020	0.262364	0.203701
Psychological GBV & Drug use disorders		
Gilbert 2012	-0.28768	0.362122
Exner-Cortens 2013	0.336472	0.184593
Exner-Cortens 2013	0.307485	0.192772
Ahmadabadi 2019	1.131402	0.195272
Ahmadabadi 2019	0.470004	0.233748
Ahmadabadi 2019	0.741937	0.210887
Psychological GBV & Self-harm		
Kaslow 2000	-0.19845	0.469651
Chowdhary 2008	1.358409	0.739665
Exner-Cortens 2013	0.285179	0.602493
Exner-Cortens 2013	0.751416	0.421839
Sexual violence & Maternal abortion and miscarriage		
Leung 2002	1.508512	0.46513
Hailu 2023	0.708036	0.639344
Johri 2011	0.652325	0.374917
Bourassa 2007	1.98238	0.566957
Larsen 2016	0.993252	0.080503
Larsen 2016	0.470004	0.18429
Sexual violence & Anxiety disorders		
Hedtke 2008	0.985817	0.279159
Elklit 2013	1.83418	0.351403
Austin 2020	0.662688	0.106644
Austin 2020	0.431782	0.209634
Sexual violence & Major depressive disorder		
Ali 2009	1.108563	0.358281
Hedtke 2008	1.007958	0.249641

Benjet 2020	0.751416	0.201461
Austin 2020	0.57098	0.098733
Austin 2020	0.788457	0.23703
Sexual violence & Drug use disorders		
Gilbert 2012	0.858662	0.362295
Nowotny 2013	0.09531	0.271216
Danielson 2009	1.23256	0.340638
Danielson 2009	0.576613	0.483959
Austin 2020	0.336472	0.175557
Austin 2020	0.476234	0.322566
Sexual violence & HIV/AIDS		
Maman 2002	0.871293	0.347782
Quigley 2000	2.059239	0.921846
Wyatt 2002	1.089532	0.214071
Jewkes 2010	-0.0202	0.388576
Abrahams 2021	0.463734	0.22916
Larsen 2016	3.637586	0.74176
Kouyoumdjian 2013	0.457425	0.127812
Birdthistle 2013	-0.07257	0.345372
BurgueÃ±o 2017	0.95935	0.173371
Deyessa 2018	0.392042	0.397709
Deyessa 2018	1.695616	0.416864
Sexual violence & STIs excluding HIV		
Chowdhary 2008	1.515127	0.514824
Allsworth 2009	0.392042	0.489274
Weiss 2008	1.098612	0.467495
Larsen 2016	1.098612	0.117228
