# Supplementary material for: The health effects associated with physical, sexual and psychological gender-based violence against men and women: a Burden of Proof study
Source: Nat Hum Behav. 2025 Apr 10;9(6):1201–16. doi: 10.1038/s41562-025-02144-2 (PMC12185316; doi:10.1038/s41562-025-02144-2)
Supplement: Supplementary file 2 — Reporting Summary [file 41562_2025_2144_MOESM2_ESM.pdf]

nature portfolio

Corresponding author(s): Emmanuela Gakidou, MSc, PhD

Last updated by author(s): Jan 24, 2025

Reporting Summary

Nature Portfolio wishes to improve the reproducibility of the work that we publish. This form provides structure for consistency and transparency in reporting. For further information on Nature Portfolio policies, see our Editorial Policies and the Editorial Policy Checklist.

Statistics

For all statistical analyses, confirm that the following items are present in the figure legend, table legend, main text, or Methods section.

n/a	Confirmed	
☐	☒	The exact sample size (*n*) for each experimental group/condition, given as a discrete number and unit of measurement
☐	☒	A statement on whether measurements were taken from distinct samples or whether the same sample was measured repeatedly
☐	☒	The statistical test(s) used AND whether they are one- or two-sided<br>*Only common tests should be described solely by name; describe more complex techniques in the Methods section.*
☐	☒	A description of all covariates tested
☐	☒	A description of any assumptions or corrections, such as tests of normality and adjustment for multiple comparisons
☐	☒	A full description of the statistical parameters including central tendency (e.g. means) or other basic estimates (e.g. regression coefficient) AND variation (e.g. standard deviation) or associated estimates of uncertainty (e.g. confidence intervals)
☐	☒	For null hypothesis testing, the test statistic (e.g. *F*, *t*, *r*) with confidence intervals, effect sizes, degrees of freedom and *P* value noted<br>*Give P values as exact values whenever suitable.*
☐	☒	For Bayesian analysis, information on the choice of priors and Markov chain Monte Carlo settings
☒	☐	For hierarchical and complex designs, identification of the appropriate level for tests and full reporting of outcomes
☐	☒	Estimates of effect sizes (e.g. Cohen's *d*, Pearson's *r*), indicating how they were calculated

Our web collection on statistics for biologists contains articles on many of the points above.

Software and code

Policy information about availability of computer code

Data collection	No primary data collection was carried out for this analysis.
Data analysis	All code used for these analyses is publicly available online (https://github.com/ihmeuw-msca/burden-of-proof). Analyses were carried out using R version 4.0.5 and Python version 3.10.9. The MR-BRT (meta-regression—Bayesian, regularized, trimmed) tool was used to generate the Burden of Proof Risk Function.

For manuscripts utilizing custom algorithms or software that are central to the research but not yet described in published literature, software must be made available to editors and reviewers. We strongly encourage code deposition in a community repository (e.g. GitHub). See the Nature Portfolio guidelines for submitting code & software for further information.

Data

Policy information about availability of data

All manuscripts must include a data availability statement. This statement should provide the following information, where applicable:

- Accession codes, unique identifiers, or web links for publicly available datasets
- A description of any restrictions on data availability
- For clinical datasets or third party data, please ensure that the statement adheres to our policy

The findings from this study are supported by data extracted from published literature databases (PubMed, Embase, CINAHL, PsycINFO, Global Index Medicus, Cochrane, and Web of Science Core Collection). Citations for all input data are provided as part of this manuscript. Study characteristics and all included data points

are provided in the Supplementary Information (Supplementary Table S1, S28). Details on data sources can also be found on the GHDx website (https://ghdx.healthdata.org/record/ihme-data/gbv-health-effects-bop-risk-outcome-scores).

Research involving human participants, their data, or biological material

Policy information about studies with human participants or human data. See also policy information about sex, gender (identity/presentation), and sexual orientation and race, ethnicity and racism.

Reporting on sex and gender	No primary data collection was carried out for this analysis, so the study does not involve human research participants. As stated in the methods overview, our estimates reflect men and women, drawing upon all available data regardless of how or if the input study collected and reported data by sex or gender. When we had available data, we performed sensitivity analysis by gender.
Reporting on race, ethnicity, or other socially relevant groupings	*Please specify the socially constructed or socially relevant categorization variable(s) used in your manuscript and explain why they were used. Please note that such variables should not be used as proxies for other socially constructed/relevant variables (for example, race or ethnicity should not be used as a proxy for socioeconomic status).*<br>*Provide clear definitions of the relevant terms used, how they were provided (by the participants/respondents, the researchers, or third parties), and the method(s) used to classify people into the different categories (e.g. self-report, census or administrative data, social media data, etc.)*<br>*Please provide details about how you controlled for confounding variables in your analyses.*
Population characteristics	No primary data collection was carried out for this analysis, so the study does not involve human research participants. The analysis evaluated the effect of physical, sexual, and psychological violence against women, and intimate partner violence and sexual violence against men on selected disease endpoints. We accepted studies regardless of the target population and exposure to violence that occurred ever, in the last three years, the past year, the past six months, the past three months, and during pregnancy in a few instances. For physical, sexual, and psychological violence, study samples represented women populations. For intimate partner violence and sexual violence, study samples represented men populations.
Recruitment	No primary data collection was carried out for this analysis, so we did not recruit participants.
Ethics oversight	This study was approved by the University of Washington IRB Committee (study #9060).

Note that full information on the approval of the study protocol must also be provided in the manuscript.

Field-specific reporting

Please select the one below that is the best fit for your research. If you are not sure, read the appropriate sections before making your selection.

☒ Life sciences ☐ Behavioural & social sciences ☐ Ecological, evolutionary & environmental sciences

For a reference copy of the document with all sections, see nature.com/documents/nr-reporting-summary-flat.pdf

Life sciences study design

All studies must disclose on these points even when the disclosure is negative.

Sample size	This investigation relied on existing published data. No statistical method was used to predetermine sample sizes. The number of studies included was determined through a systematic literature review that included title/abstract screening, full-text screening, and citation searching to identify relevant articles and extract data points used as input to models. Details surrounding the sample size of each included study can be found in Supplementary Table SI and the number of included studies per risk-outcome pair is reported in Table 2.
Data exclusions	As described in Supplementary Information Section 4.2, reports were excluded based on the following exclusion criteria:<br>Study design: Cross-sectional, ecological, case series or case studies.<br>Participants: Studies conducted in subgroups identified only by convenience sampling or subgroups identified via a shared characteristic that is likely related to risk of exposure to violence or the reported health outcome (e.g., domestic violence shelter residents).<br>Exposure measurement: Studies that report only an aggregate measure of exposure combining exposure to a form of violence with other, non-eligible exposures (e.g., reports a composite ACE score only) will be excluded. For these studies, we are unable to disentangle the effect of violence exposure from the effects of other hardships or exposure types, preventing their inclusion in our review.<br>Does not meet minimum reporting criteria: Studies missing essential data, that is, those that do not report effect sizes and uncertainty information (confidence intervals, sample sizes) or the data needed to impute an effect size with uncertainty information.
Replication	This is a meta-analysis of existing studies with many years of cohort and other data. The code and data used are publicly available, and the analyses could theoretically be replicated.
Randomization	This analysis is a meta-analysis of existing studies and thus, there were no experimental groups.
Blinding	N/A. This study was a meta-analyses using existing data and did not the collection of primary data nor experimental/control groups. As such, blinding was not relevant.

Reporting for specific materials, systems and methods

We require information from authors about some types of materials, experimental systems and methods used in many studies. Here, indicate whether each material, system or method listed is relevant to your study. If you are not sure if a list item applies to your research, read the appropriate section before selecting a response.

Materials & experimental systems

n/a	Involved in the study
☒	☐ Antibodies
☒	☐ Eukaryotic cell lines
☒	☐ Palaeontology and archaeology
☒	☐ Animals and other organisms
☒	☐ Clinical data
☒	☐ Dual use research of concern
☒	☐ Plants

Methods

n/a	Involved in the study
☒	☐ ChIP-seq
☒	☐ Flow cytometry
☒	☐ MRI-based neuroimaging

Plants

Seed stocks	*Report on the source of all seed stocks or other plant material used. If applicable, state the seed stock centre and catalogue number. If plant specimens were collected from the field, describe the collection location, date and sampling procedures.*
Novel plant genotypes	*Describe the methods by which all novel plant genotypes were produced. This includes those generated by transgenic approaches, gene editing, chemical/radiation-based mutagenesis and hybridization. For transgenic lines, describe the transformation method, the number of independent lines analyzed and the generation upon which experiments were performed. For gene-edited lines, describe the editor used, the endogenous sequence targeted for editing, the targeting guide RNA sequence (if applicable) and how the editor was applied.*
Authentication	*Describe any authentication procedures for each seed stock used or novel genotype generated. Describe any experiments used to assess the effect of a mutation and, where applicable, how potential secondary effects (e.g. second site T-DNA insertions, mosiacism, off-target gene editing) were examined.*
